# Migration of the intertropical convergence zone driven by ocean circulation changes

**DOI:** 10.1038/s41467-026-73200-2

**Published:** 2026-05-16

**Authors:** Yaru Guo, Aixue Hu, Gerald A. Meehl, Maria J. Molina, Hui Li, Katinka Bellomo, Nan Rosenbloom

**Affiliations:** 1 National Center for Atmospheric Research, Boulder, CO USA; 2 Key Laboratory of Ocean Observation and Forecasting and Key Laboratory of Ocean Circulation and Waves, Institute of Oceanology, Chinese Academy of Sciences, Qingdao, China; 3 University of Maryland, College Park, MD USA; 4 Department of Geosciences, University of Padova, Padova, Italy

**Keywords:** Climate and Earth system modelling, Projection and prediction, Atmospheric dynamics, Ocean sciences

## Abstract

The Intertropical Convergence Zone (ITCZ) is essential to global precipitation patterns and has major socioeconomic and environmental impacts. Yet its future position remains uncertain. Here, using reanalysis data, Earth system model simulations, and targeted sensitivity experiments, we assess risks of the ITCZ crossing any thresholds and find only about one-third of CMIP6 models with a more northern ITCZ mean position-closer to the observed climatological state-simulate a southward ITCZ shift consistent with recent observations. This shift requires a substantial weakening of the Atlantic Meridional Overturning Circulation (AMOC), while the warmer base state alone is insufficient to push it farther north. Furthermore, a collapsed AMOC pushes the ITCZ into the Southern Hemisphere without a warmer base state, and toward the equator when combined with a warmer base state—both cases leading to significant changes in global rainfall patterns. These findings imply that the observed southward ITCZ migration over recent decades is consistent with a weakened AMOC, within the context of coupled atmosphere-ocean interactions.

## Abstract (web summary)

This study shows that only about one-third of CMIP6 models, those with a more northern ITCZ mean position closer to the observed climatology, simulate a southward ITCZ shift consistent with the observations, which requires a substantial AMOC weakening along with a warmer base state.

## Introduction

The Intertropical Convergence Zone (ITCZ) produces nearly one-third of global precipitation^1^ and primarily resides in the warmer Northern Hemisphere. It is closely linked to Hadley Cells (HCs) with the intense ascending motion over the ITCZ and the descending motion near the subtropical dry zones^2^. Even a modest shift in the ITCZ position can profoundly affect the distribution and frequency of droughts and floods, influence tropical forest ecosystems, and impact the livelihoods of surrounding communities^1,3^. However, the mechanisms governing ITCZ migration under ongoing warming remain poorly understood, and the potential risk of the ITCZ crossing critical thresholds is largely unknown.

Within the atmospheric energy-flux theory, the ITCZ aligns near the energy flux equator^4–6^, whose position is determined by cross-equatorial atmospheric heat transport (AHT_eq_) and net energy input into the atmosphere at the equator (Qnet_eq_)^1,4,7,8^. AHT_eq_ is mainly controlled by the interhemispheric asymmetry of the HC and the inter-tropical surface temperature (TS) contrast in association with the extratropical forcing and feedback^9^. The HC contrast is strongly governed by the interhemispheric sea surface temperature (SST) gradient. Any extratropical forcing or asymmetric climate background that alters this SST contrast leads to a shift in the ITCZ toward the warmer hemisphere, driven by the adjustment of HC intensity and the AHT_eq_ anomaly to balance the global energy budget^7,10,11^. In contrast, Qnet_eq_ is influenced by tropical forcing, such as El Niño^1,10,12^. Moreover, AHT_eq_ has been identified as the predominant driver in displacing the energy flux equator and ITCZ under a quasi-equilibrium condition^4,6^. The AHT_eq_ is aligned with the direction of flow in the upper branch of the HC at the equator and is primarily controlled by the intensity contrast between its Southern and Northern Hemisphere branches. Therefore, the annual mean Southern Hemisphere HC is stronger and extends across the equator into the Northern Hemisphere, which leads to a southward flow and AHT_eq_, and the ITCZ is positioned in the Northern Hemisphere. Changes in HC intensity occur predominantly in the Northern Hemisphere rather than the Southern Hemisphere in a warmer base state^13^. Several mechanisms have been proposed to explain variations in Northern Hemisphere HC strength, including changes in moisture and thermodynamic balances^14^, the direct radiative effects of CO_2_ on temperature^15,16^, and modifications of meridional temperature gradients^12,17,18^.

Based on this theory, changes in AHT_eq_ and oceanic meridional heat transport across the equator (OHT_eq_) together determine the position of the energy flux equator and thus the ITCZ. While AHT_eq_ is the dominant driver under quasi-equilibrium atmospheric conditions, OHT_eq_ can also act as an active forcing—for example, the Atlantic Meridional Overturning Circulation (AMOC) weakening reduces northward OHT_eq_, requiring a compensating reduction in southward AHT_eq_ and thus a southward ITCZ displacement^7,8^. Under equilibrium conditions, OHT_eq_ and AHT_eq_ covary^19,20^ and are mechanically coupled over low latitudes^21–24^. Under transient climate conditions, OHT_eq_ and AHT_eq_ can operate semi-independently. A major driver influencing both OHT_eq_ and AHT_eq_ is the AMOC. A much weakened or collapsed AMOC—such as that simulated in freshwater hosing only experiments and in the paleo proxy records^25,26^—alters OHT, reduces or reverses interhemispheric TS and HC contrasts, and results in a southward ITCZ shift^8,27–31^.

In a continuous warming world, models project a consistent and robust AMOC slowdown, largely driven by surface warming^32^ and increased melted water from the Arctic^33^ and ice sheets^34^. This weakening reduces northward OHT^35^, suppresses warming over the subpolar North Atlantic^32,36,37^, and tends to move the ITCZ southward. Meanwhile, the concurrently intensified interhemispheric HC and TS contrasts act to move ITCZ northward^38–40^. Thus, the net effect of coupled ocean–atmosphere changes on the ITCZ position remains uncertain^41^ and requires further investigation.

Here we examine the relative contributions of atmospheric and oceanic circulation changes to the annual and zonal mean ITCZ position (hereafter we simply use “ITCZ” to represent the annual and zonal mean ITCZ) and explore the processes driving the ITCZ across potential tipping point by analyzing the fifth generation European Center for Medium-Range Weather Forecasts (ECMWF) reanalysis (ERA5)^42^, the Japanese 55-year Reanalysis (JRA-55)^43^, the National Centers for Environmental Prediction (NCEP)^44^, the ECMWF Twentieth Century Reanalysis (ERA-20C)^45^, model experiments with a warmer climate (represented here by the idealized 1% CO_2_ experiments (hereafter simply 1%CO_2_) from the Coupled Model Intercomparison Project Phase 6 (CMIP6)), and a suite of specially designed sensitivity experiments using the Community Earth System Model version 2 (CESM2; “Methods”)^46^ and the EC-Earth3 model^47^. Throughout this study, we use the CMIP6 1%CO_2_ experiment to approximate the climate response under a CO_2_ quadrupling condition by using the mean state over years 131-150 of the 1%CO_2_ simulation when atmospheric CO_2_ reaches approximately four times its preindustrial level. ERA5, JRA-55, NCEP, and ERA-20C provide insight into observed ITCZ responses to external forcing and provide a benchmark to evaluate the processes associated with model projections. Notably, our analysis shows that models projecting a southward ITCZ migration align more closely with reanalysis data. In these models and reanalysis, the mean ITCZ position is more northern, and its southward shift is driven by a pronounced AMOC slowdown. Under this framework, the observed southward ITCZ migration in recent decades is consistent with, but does not uniquely imply an ongoing weakening of the AMOC. In contrast, in models with a more southern mean ITCZ position, warming tends to shift the ITCZ northward. However, when the ITCZ is already in a more northern position, further warming appears insufficient to drive it farther north. The remainder of the paper is organized as follows. We first present the observed and simulated ITCZ responses to increasing CO_2_ forcing. We then examine the underlying dynamical mechanisms driving the ITCZ shifts, with a focus on the relative roles of atmospheric processes and AMOC changes and assess the conditions under which a possible tipping point may emerge. Finally, we place these results in the context of associated AMOC variations and discuss their broader implications.

## Results

### Observed and projected ITCZ migration

There are multiple ways to define the ITCZ—precipitation-based methods (e.g., the latitude of maximum precipitation^48^, the precipitation centroid^49^, the brightness temperature^50^), and the atmospheric circulation-based methods (e.g., vertical pressure velocity^51^, and the boundary between northern and southern HCs^41^). Although the choice of ITCZ definition can affect the absolute ITCZ position and its response to external forcing (Supplementary Fig. 1), it may not affect the interpretation of the underlying physical processes. Because we will use the atmospheric energy constraint theory to explain the physical processes, we therefore adopt an atmospheric circulation-based ITCZ definition in this study^41^ as this energy constraint theory provides a close relationship between HCs and AHT_eq_.

**Fig. 1:**
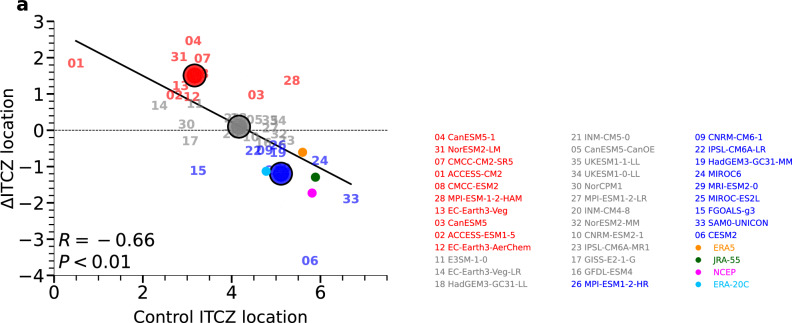
Relationships in the Intertropical Convergence Zone (ITCZ) location and ITCZ migration. **a** Relationships across the Coupled Model Intercomparison Project Phase 6 (CMIP6) models between climatological mean ITCZ position and ITCZ migration from the preindustrial control (hereafter “Control”) to the late-stage response of the CMIP6 1%CO_2_ experiment, defined as the mean over years 131–150, when atmospheric CO_2_ reaches approximately four times its preindustrial level. The historical ITCZ location based on the fifth generation European Center for Medium-Range Weather Forecasts (ECMWF) reanalysis (ERA5; orange dot), the Japanese 55-year Reanalysis (JRA-55; green dot), the National Centers for Environmental Prediction (NCEP; magenta dot), and the ECMWF Twentieth Century Reanalysis (ERA-20C; cyan dot) are approximately 5.50°N over 1940–2024, 5.89°N over 1958–2023, 5.81°N over 1948–2023, and 4.78°N over 1940–2010. The ITCZ migration is calculated as the ITCZ difference from the early period (1940–1969, 1958–1987, 1948–1977, and 1940–1969) to the recent period (1995–2024, 1994–2023, 1994–2023, and 1981–2010). The Northward, Southward, and Neutral Group ensembles are shown separately.

To explore the ITCZ changes in ERA5, JRA-55, NCEP, and ERA-20C reanalysis, we define the difference between the ITCZ location in the first and last 30-year mean in each of the reanalysis as the ITCZ migration in that reanalysis. All of the reanalyses show a clear southward ITCZ migration by 0.61° (ERA5), 1.29° (JRA55), 1.73° (NCEP), and 1.13° (ERA20C) from the early period (6.06°N over 1940–1969 (ERA5), 6.54°N over 1958–1987 (JRA55), 6.83°N over 1948–1977 (NCEP), and 5.39°N over 1940–1969 (ERA20C)) to recent period (5.45°N over 1995–2024, 5.24°N over 1994–2023, 5.10°N over 1994–2023, and 4.25°N over 1981–2010), respectively, while CMIP6 models project inconsistent ITCZ migrations at approximately CO_2_ quadrupling in the 1%CO_2_ experiment (mean over years 131–150; Fig. 1). Among the 35 CMIP6 models analyzed (Supplementary Table 1), approximately one-third simulate a substantial northward shift (1.51° ± 0.53°), another third shows a pronounced southward shift (–1.20° ± 0.95°), and the remnant indicates little change (0.10° ± 0.34°; Fig. 1). Thus, we classify the models into three groups based on the direction of ITCZ shifts: Northward (red numbers), Southward (blue numbers), and Neutral (gray numbers) Groups where the big solid circles represent the ensemble mean of each group (Fig. 1). The ensemble-mean ITCZ location is closest to reanalysis (ensemble mean 5.52° ± 0.51°N) for Southward Group (5.18° ± 0.88°N), much less for Northward Group (3.17° ± 1.20°N) with Neutral Group in the middle (4.18° ± 0.86°N). Moreover, the temporal standard deviation of the ITCZ position is much larger for the Northward Group (1.60 ± 0.49°) than for other groups (1.14 ± 0.38° for Neutral Group and 0.82 ± 0.23° for Southward Group), and this temporal standard deviation is 0.86° for ERA5, 0.79° for JRA-55, 1.03° for NCEP, and 0.87° for ERA-20C. Overall, the Southward Group best captures the observed ITCZ mean state and variability. The southward ITCZ migration observed in reanalysis is also consistent with the southward response of the Southward Group in response to CO_2_ quadrupling, although the drivers of the historical ITCZ southward migration may differ from those driving the CO_2_-forced response.

### ITCZ position and the governing processes

**Fig. 2:**
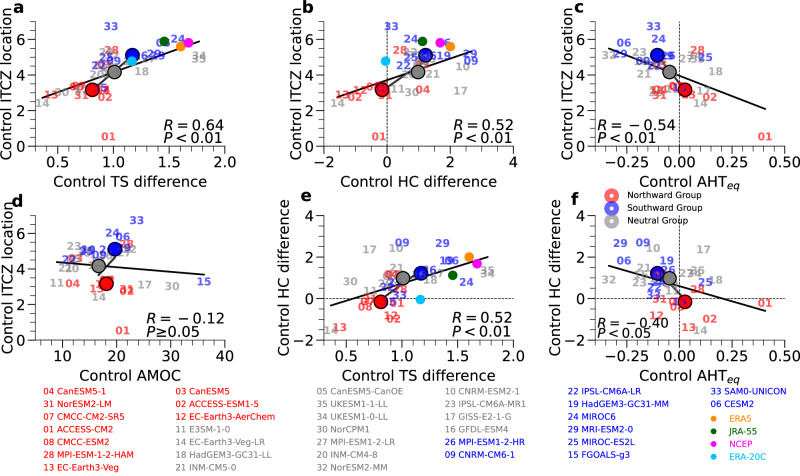
Relationships in preindustrial control simulations. **a–d** Relationships across CMIP6 models between the mean ITCZ position and: the tropical surface temperature (TS) difference between 0°–30°N and 0°–30°S (**a**); hemispheric Hadley Cell (HC) intensity difference (**b**); the northward cross-equatorial atmospheric heat transport (AHT_eq_) (**c**); and the Atlantic Meridional Overturning Circulation (AMOC) strength (**d**). **e**,** f** Relationships between HC intensity difference and: the TS difference (**e**), and the northward AHT_eq_ (**f**). ERA5 (orange dot), JRA-55 (green dot), NCEP (magenta dot), and ERA-20C (cyan dot) reanalysis data represent the average over 1940–2024, 1958–2023, 1948–2023, and 1940–2010, respectively. The correlation (**R**) and *P*-value of a linear fit (black solid line) are also shown. The Northward, Southward, and Neutral Group ensembles are shown.

We first analyze the equilibrium-climatological mean ITCZ location in preindustrial control (hereafter “Control”; Fig. 2). In general, ITCZ location in CMIP6 models is linearly related to tropical interhemispheric TS contrast (*R* = 0.64; Fig. 2a), interhemispheric HC contrast (*R* = 0.52; Fig. 2b) and southward AHT_eq_ (*R* = -0.54; Fig. 2c), agreeing with the atmospheric energy-flux theory^1,8,41,52^. As such, a more northern ITCZ position corresponds to a stronger southward AHT_eq_ along with greater interhemispheric TS and HC intensity contrasts (Fig. 2a, b), highlighting the dynamical coupling of the HC with TS contrast (*R* = 0.52; Fig. 2e) or AHT_eq_ (*R* = -0.40; Fig. 2f). Most Southward Group models are consistent with reanalysis, where a positive interhemispheric HC contrast aligns with a southward AHT_eq_ (Fig. 2f). However, the opposite changes are shown in most Northward Group models, such as a stronger northern HC than southern HC and a northward AHT_eq_, contradicting an ITCZ located in the Northern Hemisphere. This implies some considerably biased physical processes in this group of models in representing ITCZ-related processes. Additionally, the ITCZ mean position shows no clear correlation with AMOC mean strength (Fig. 2d). But if only the Southward Group is considered, a stronger AMOC is related to a more northward ITCZ (Supplementary Fig. 2b), consistent with the notion that a stronger AMOC transports more heat from the Southern to the Northern Hemisphere, which increases the interhemispheric temperature contrast. On the other hand, in the Northward Group, a stronger AMOC is related to a more southward ITCZ location, although this relationship is not statistically significant (Supplementary Fig. 2a). Therefore, this analysis further suggests that the southward group can represent the ITCZ and its associated processes better.

**Fig. 3:**
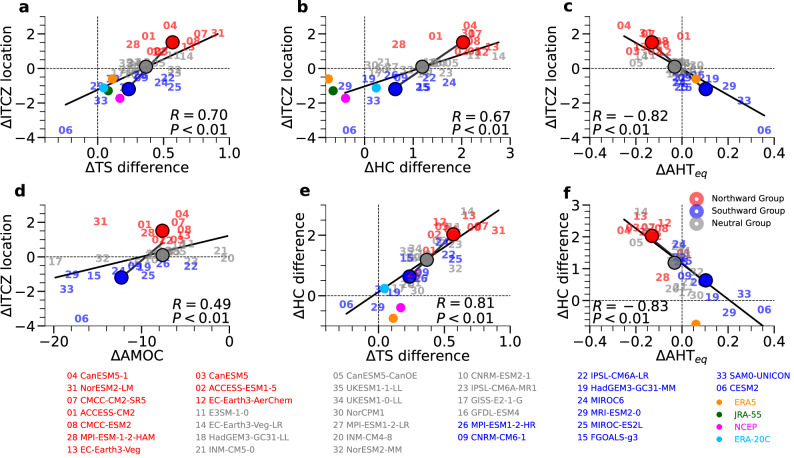
Relationships in changes between CO_2_ quadrupling (mean over years 131–150 of 1%CO_2_) and the Control simulation. **a**–**d** Relationships across CMIP6 models between the mean ITCZ position and: the tropical TS difference between 0°–30°N and 0°–30°S (**a**); hemispheric HC intensity difference (**b**); the northward AHT_eq_ (**c**); and AMOC strength (**d**). **e**, **f** Relationships between HC intensity difference and: the TS difference (**e**), and the northward AHT_eq_ (**f**). Changes in ERA5 (orange dot), JRA-55 (green dot), NCEP (magenta dot), and ERA-20C (cyan dot) reanalyses represent differences between 1995–2024 and 1940–1969, 1994–2023 and 1958–1987, 1994–2023 and 1948–1977, and 1981–2010 and 1940–1969, respectively. The correlation (**R**) and *P*-value of a linear fit (black solid line) are also shown. The Northward, Southward, and Neutral Group ensembles are shown.

When CO_2_ quadruples, the previously noted linear relationships weaken considerably (Supplementary Fig. 3), indicating a lessened influence of the atmospheric processes. However, the correlations between ITCZ movement and changes in interhemispheric TS contrast, HC intensity contrast and AHT_eq_ in response to CO_2_ quadrupling in the 1%CO_2_ experiment become substantially stronger ($\left|{\mbox{R}}\right|\ge$0.67; Fig. 3a–c) compared with Control simulations, as do the linkages between changes in HC intensity and TS contrasts or AHT_eq_ ($\left|{\mbox{R}}\right|\ge$0.81; Fig. 3e, f). Further analysis shows that enhanced interhemispheric TS and HC intensity contrasts with elevated CO_2_ cannot always lead to strengthened southward AHT_eq_ and a northward ITCZ shift—particularly those in the Southward Group (Fig. 3a–c, e, f), although both HC and TS contrasts increase, the ITCZ moves southward instead of northward in this group. This reinforces the idea that changes in ocean circulation, such as a weakening AMOC, may overpower the atmospheric drivers of ITCZ migration, as evidenced by the significant correlation between AMOC changes and ITCZ migration (*R* = 0.49; Fig. 3d) in a warmer climate. This means that a larger weakening of the AMOC is related to a more southward ITCZ movement. However, Supplementary Fig. 2d and e show that this relationship is true only for the Southward Group, with an opposite relationship for the Northward Group—larger AMOC weakening is associated with a more northward ITCZ movement, which suggests that changes in AMOC play a very minor role in determining the ITCZ movement in response to the greenhouse gas forcing, and the atmospheric processes play a more dominant role. Therefore, this result demonstrates that the relationship between AMOC and ITCZ location does not depend on the background mean states. For models with a stronger AMOC-ITCZ relationship in their preindustrial mean state, AMOC also plays an important role in determining the ITCZ’s transient response. For models with weak or no AMOC-ITCZ relationships in their preindustrial mean state, AMOC plays only a minor role in the ITCZ’s transient behavior.

### The physical mechanisms

To investigate the physical processes governing ITCZ migration under CO₂ quadrupling, we examine changes in both atmospheric and oceanic circulations. In the Northward Group, there is a consistent increase in interhemispheric TS contrast (+0.57 ± 0.20 °C; Fig. 3a), HC intensity contrast (+2.03 ± 0.55 × 10^10^ kg s^−1^; Fig. 3b), and southward AHT_eq_ (–0.13 ± 0.07 PW; Fig. 3c), which support a northward ITCZ migration. Although the global northward OHT_eq_ reduces by 74.01% (or -0.34 ± 0.09 PW; Supplementary Fig. 4a), which tends to shift the ITCZ southward, the overall northward ITCZ movement in this model group indicates that the oceanic circulation only partially offsets the dominant atmospheric contribution to the northward ITCZ migration in the Northward Group. In contrast, in the Southward Group, the increases in interhemispheric TS (+0.24 ± 0.26 °C; Fig. 3a) and HC intensity contrasts (+0.63 ± 0.74 × 10^10^ kg s^−1^; Fig. 3b) are more moderate (less than 50% of the changes in the Northward Group), which tends to move ITCZ northward. However, the southward AHT_eq_ reduces by +0.10 ± 0.13 PW (Fig. 3c), which favors a southward ITCZ movement. The inconsistency between the TS/HC contrast and the southward AHT_eq_ indicates that the southward AHT_eq_ is controlled not only by the changes in atmospheric circulation and temperature, but also by the moisture changes. Moreover, the substantially weakened global northward OHT_eq_ (by 75.96% or –0.25 ± 0.06 PW; Supplementary Fig. 4b) acts to shift the ITCZ southward. Since changes in OHT_eq_ are much larger than AHT_eq_, OHT_eq_ dominates the ITCZ movement southward in the Southward Group. The enhancements in HC and TS contrasts are a result of a stronger AMOC decline, as illustrated next.

**Fig. 4:**
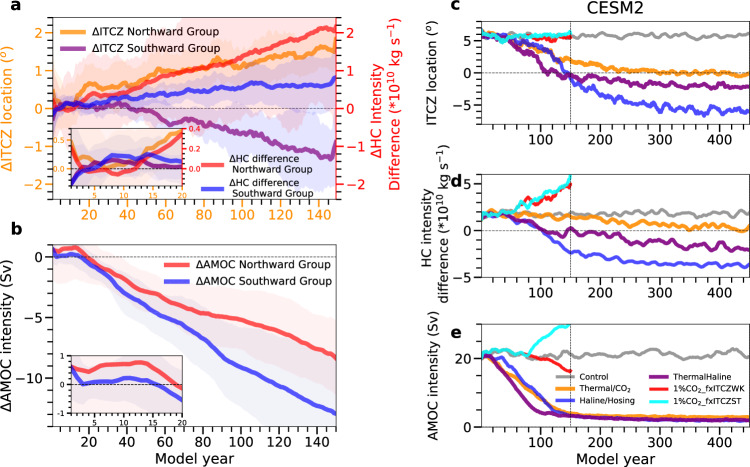
ITCZ, HC intensity difference, and AMOC intensity. **a** (left axis) Transient evolution of ensemble-mean changes in the 11-year running mean ITCZ location and (right axis) interhemispheric HC intensity contrast from the 150-year 1%CO_2_ experiment, relative to the respective Control simulations for the Northward and Southward Groups. Shading indicates one standard deviation for each group. During the first 20 years of the 1%CO_2_ experiment, the ITCZ shifts northward by +0.27 ± 0.49° and +0.09 ± 0.21° in the Northward and Southward Groups, respectively. **b** Same as **a**, but for changes in AMOC strength. **c–e** The 11-year running mean ITCZ location (**c**), HC intensity difference (**d**), AMOC intensity (**e**) from the Community Earth System Model version 2 (CESM2) Control (gray), Thermal/CO_2_ (orange), Haline/Hosing (blue), ThermalHaline (purple), 1%CO₂_fxITCZWK (red), and 1%CO₂_fxITCZST (cyan) simulations.

Further analysis reveals that for the Southward Group, the reduction in total northward OHT_eq_ is primarily driven by a much larger decrease in the Atlantic, combined with a small reduction in southward OHT_eq_ in the Indo-Pacific basin (Supplementary Fig. 4b). This pattern reflects stronger AMOC weakening and highlights AMOC’s dominant role in controlling the OHT_eq_ in both basins^36^ (Fig. 4b). In contrast, a moderate reduction in the Atlantic OHT_eq_ for the Northward Group is accompanied by enhanced southward OHT_eq_ in the Indo-Pacific, leading to a larger overall OHT_eq_ reduction (Supplementary Fig. 4a). The Atlantic OHT_eq_ reduction is associated with more moderate AMOC weakening, while the Indo-Pacific enhancement is driven by more pronounced warming in the northern tropical oceans.

In Control, the ensemble mean AHT_eq_ is near zero in the Northward Group but southward in the Southward Group—though with smaller amplitude than observed in ERA5 (Supplementary Fig. 4c, d). Despite this, mean AHT_eq_ can be used to explain the ITCZ location in both the Southward Group and ERA5, consistent with the atmospheric energy-flux theory, though less so for the Northward Group. In 1%CO_2_, AHT_eq_ changes in the Southward Group closely resemble the ERA5 change from 1940–1969 to 1995–2024, while the Northward Group exhibits changes of the opposite sign (Supplementary Fig. 4c, d). Overall, the absolute magnitude of AHT_eq_ is closely related to the mean ITCZ position in Control, and AHT_eq_ changes in a warmer climate are linked to ITCZ migration across all models—with the direction of ITCZ movement closely tied to its mean state.

Next, we examine the time evolution of processes influencing ITCZ migration. During the first 20 years of the 150-year 1%CO₂ experiment, both the Northward and Southward Groups simulate a slight northward ITCZ displacement (Fig. 4a), following previous findings^4^, though this initial shift is not statistically significant in either group. The relatively larger northward migration in the Northward Group is predominantly attributed to the strengthened AMOC and TS and HC intensity contrasts. The AMOC enhancement may be linked to increased evaporation in the subtropical North Atlantic, leading to enhanced deep convection in the subpolar North Atlantic. However, the Southward Group shows relatively smaller northward ITCZ shift, along with smaller changes in OHT_eq_, TS and HC intensity contrasts. This discrepancy may be linked to differences in the mean ITCZ position between the two groups.

After year 20, the ITCZ continues to migrate northward in the Northward Group, with sustained increases in southward AHT_eq_ and amplification of TS and HC intensity contrasts (Fig. 4a, Supplementary Fig. 5a, c). As AMOC weakens moderately, its effect on reducing northward Atlantic OHT_eq_ is reinforced by a modest southward OHT_eq_ strengthening in the Indo-Pacific basin, resulting in a substantial total northward OHT_eq_ reduction (Supplementary Fig. 4a). Nevertheless, the impact of the significantly stronger warming in the Northern Hemisphere outweighs the opposing contribution from northward OHT_eq_ decline, ultimately driving the ITCZ northward (Fig. 4a).

In contrast, the Southward Group exhibits a delayed southward ITCZ migration, beginning after year 50—once AMOC weakening becomes large enough to overcome the moderate increases in the TS and HC intensity contrasts (Fig. 4b, d, Supplementary Fig. 5b, d). Resulting from this interaction, the interhemispheric TS contrast across different latitude bands in the Southward Group is roughly 50% as large as that in the Northward Group (Supplementary Fig. 5c, d). These findings suggest that greater AMOC weakening plays a critical role in suppressing the Northern Hemisphere warming, making oceanic changes the dominant driver of ITCZ migration. Conversely, in the Northward Group, initial AMOC strengthening facilitates more rapid Arctic warming amplification (Supplementary Fig. 6), resulting in the dominance of atmospheric processes.

Since the Southward Group, including CESM2, better represents ITCZ migration observed in reanalyses, we use CESM2 as a representative model to explore the complex interplay between a warmer climate and AMOC weakening on ITCZ migration and the potential ITCZ tipping point. In addition to the Control simulation, we conducted five additional experiments, including Thermal/CO₂ with 150-year 1%CO₂ and 300-year extension at fixed CO_2_ quadrupling, Haline/Hosing with 150-year ramping up hosing and 300-year extension with fixed hosing, ThermalHaline representing a combination of both, as well as 1%CO₂_fxITCZWK and 1%CO₂_fxITCZST with 150-year 1%CO₂ under artificially constrained relatively weak and strong AMOC states, respectively (“Methods”).

In the Control, the ITCZ position and interhemispheric TS and HC intensity contrasts agree well with reanalysis (Fig. 4c, d, Supplementary Fig. 7b). However, the AHT_eq_ is only about one-third of that in ERA5. Despite being roughly one-fifth of some observed estimates^53,54^, Control OHT_eq_ aligns well with the Ocean Reanalysis System 5 (ORAS5; Supplementary Fig. 4), underscoring the considerable uncertainty from limited observations in determining OHT_eq_. The southward ITCZ migration in CESM2 occurs only after significant AMOC weakening. The more rapid ITCZ shift in ThermalHaline compared to Haline/Hosing before year 150 is mainly due to a faster AMOC decline due to the combined freshwater and greenhouse gas forcings (Fig. 4e). As AMOC collapses after roughly year 130 in Thermal/CO₂, ThermalHaline and Haline/Hosing experiments, the southward ITCZ movement gradually settles in different latitudes with a southmost location in Haline/Hosing experiment and northmost in Thermal/CO_2_ experiment with a location between these two experiments in ThermalHaline. Since AMOC collapses in all these experiments, the reduced southward ITCZ migration in the Thermal/CO₂ and ThermalHaline experiments compared to Haline/Hosing is attributed to CO₂-induced warming. The 1%CO₂_fxITCZWK and 1%CO₂_fxITCZST are designed to test whether moderate AMOC weakening or strengthening can influence ITCZ migration. Results show that the ITCZ remains largely stable in both experiments (Fig. 4c, Supplementary Fig. 7). Although the stronger AMOC enhances interhemispheric TS and HC contrasts, the increase in the southward AHT_eq_ is much moderate with a nearly unchanged ITCZ which may imply a possible existence of a northernmost ITCZ location (such as around 6°N) which puts a limit on the response of ITCZ to an AMOC stronger than the preindustrial control level.

The above southward ITCZ migration is closely associated with the weakening—and in some cases, reversal—of AHT_eq_ and interhemispheric HC and tropical TS contrasts. Across all sensitivity experiments, tropical TS contrast closely tracks interhemispheric HC intensity contrast, while mid-to-high latitude TS contrast is broadly linked to sea ice loss. For example, the amplified TS contrast in the Thermal/CO₂ and ThermalHaline experiments after around year 200 is largely attributable to an almost complete (~100%) loss in Arctic sea ice (Supplementary Figs. 7, 8). In the 1%CO₂_fxITCZWK and 1%CO₂_fxITCZST simulations, a more rapid sea ice decline due to the sustained AMOC in the first half of the experiments, with moderate weakening or strengthening in the second half of the experiments, drives much stronger TS contrast across all latitude bands and a much stronger polar amplification. Notably, a sign change in AHT_eq_ (e.g., from southward to northward) does not automatically induce an ITCZ shift from Northern to Southern Hemisphere immediately, as illustrated in Haline/Hosing and ThermalHaline experiments, and ITCZ never crosses the equator in Thermal/CO₂ (Supplementary Fig. 9). Therefore, while the atmospheric energy-flux theory may effectively explain the climatological mean ITCZ, it may not fully capture ITCZ migration under transient warming.

For OHT_eq_, it remains nearly unchanged in Haline/Hosing owing to the almost completely compensated changes between the Atlantic and Indo-Pacific. OHT_eq_ in Thermal/CO₂ and ThermalHaline becomes negative due to a more pronounced decline in northward Atlantic OHT_eq._ In the 1%CO₂_fxITCZWK and 1%CO₂_fxITCZST, the northward OHT_eq_ reduces due to an increase in southward OHT_eq_ in the Indo-Pacific basin. The effect of this OHT_eq_ reduction is largely balanced by the strengthening of TS and HC intensity contrasts, leading to minimal ITCZ change. These contrasting behaviors highlight the distinct roles of forcing mechanisms: Haline/Hosing isolates the impact of AMOC weakening alone, 1%CO₂_fxITCZWK/1%CO₂_fxITCZST reflects the influence of anthropogenic CO₂ forcing only, while Thermal/CO₂ and ThermalHaline demonstrate their combined effects.

For completeness, we also examine a suite of sensitivity experiments—including fixAMOC, Hosing, and Abrupt-4 × CO_2_ (“Methods”)—using EC-Earth3, one of the models in the Northward Group, to isolate the impacts of different forcing mechanisms on ITCZ shifts. In the preindustrial control simulation, EC-Earth3 features a mean ITCZ position at 3.3°N (Supplementary Fig. 10a), consistent with the characteristics of the Northward Group. Under Abrupt-4 × CO_2_ forcing, EC-Earth3 exhibits a northward ITCZ migration (to ~4°N, primarily associated with a strengthening of the interhemispheric HC intensity contrast between the southern and northern branches (Supplementary Fig. 10b), while concurrent AMOC weakening partially offsets the northward displacement (Supplementary Fig. 10c). When the AMOC strength is constrained in the fixAMOC experiment, the ITCZ migrates farther northward (to ~6°N), indicating that the enhanced interhemispheric HC contrast exerts a dominant control on the ITCZ position in the absence of AMOC-induced compensation. In contrast, the Hosing experiment produces a southward ITCZ shift, driven mainly by pronounced AMOC weakening, with the associated reduction in interhemispheric HC intensity contrast further reinforcing the southward displacement. Together, these sensitivity experiments show that generally weakening of the AMOC induces a southward ITCZ movement and the greenhouse gas-induced warming leads to a northward ITCZ movement, a conclusion consistent with those obtained from CESM2 sensitivity experiments. The most noticeable difference between CESM2 and EC-Earth3 is the relative contribution by these two forcings—AMOC changes dominate the ITCZ movement in CESM2, and greenhouse gas forcing dominates the ITCZ movement in EC-Earth3. As a result, with elevated greenhouse gas and the resulting AMOC weakening, the ITCZ moves northward in EC-Earth3 (also other models in the Northward Group), but southward in CESM2 (also models in the Southward Group). Although our analysis cannot clearly demonstrate the exact underlying physical mechanism leading to this different ITCZ response, our speculation is that the different mean ITCZ locations in these two groups of models could be the fundamental cause. An ITCZ much closer to the equator or further away from the equator may make it more or less sensitive to the atmospheric (or oceanic) circulation changes, resulting in a different ITCZ movement.

### Possible ITCZ tipping point

Following ref. ^21^., “tipping point” refers to the ITCZ crossing a critical threshold from one stable state to another stable state under gradually increasing forcing and the present state doesn’t return to the initial state even if the drivers of the change are abated. Figure 4c shows that the ITCZ shifts further southward by approximately 2° in the Thermal/CO₂ and ThermalHaline after CO₂ stabilizes at a quadrupled level (after year 150), but by about 6° in the Haline/Hosing experiment once the freshwater forcing stabilizes. Since there is a 300-year period (from year 151 to 450) with constant forcing in all these CESM2 simulations, the ITCZ movement can be considered at a quasi-equilibrium state with the external forcing, for instance, the ITCZ location is nearly unchanged in the last 150 years (from year 300 to 450) of the Hosing/Haline, Thermal/CO₂ and ThermalHaline experiments. Based on this, the results shown in Fig. 4c suggest the existence of three quasi-stable ITCZ positions in CESM2 after year 400 under the three specific forcing scenarios examined in this study: around 6°N under preindustrial conditions with an active AMOC, near the equator when the AMOC collapses under strong greenhouse gas forcing, and around 6°S when the AMOC collapses due to freshwater forcing alone. It is worth noting that other quasi-stable ITCZ positions might exist under different forcing conditions and different AMOC stabilization levels associated with the respective forcing conditions. Moreover, the exact quasi-equilibrium position may also be model dependent, which remains to be explored in future work.

There are a few unanswered questions regarding the potential ITCZ tipping behavior from the current CESM2 sensitivity experiments. First, whether the above-mentioned ITCZ quasi-equilibrium states are true equilibrium states is still not definite, and we cannot totally rule out the possibility that what is shown here could still be a transient behavior of the ITCZ. Second, since the changes of AMOC play a crucial role in determining the ITCZ response to external forcing changes in these experiments, if the ITCZ would return to its preindustrial location after the reversal of the external forcings will depend on whether or not AMOC will recover, and if AMOC recovers, whether this AMOC recovery is linear or nonlinear will determine the linearity of the ITCZ changes in response to the external forcing changes.

Accompanying these ITCZ shifts, precipitation decreases in the Northern Hemisphere and increases in the Southern Hemisphere if the collapsed AMOC is forced by freshwater alone (Supplementary Fig. 11)^28^. However, in scenarios with both elevated CO₂ and a collapsed AMOC, precipitation increases across most latitudes except between ~5° and 30°N. The consistent drying in this band (5^o^ ~ 30°N) across three CESM2 simulations with a collapsed AMOC suggests it is primarily driven by the AMOC weakening. In contrast, the large precipitation increases near the equator, evident in all elevated CO₂ experiments regardless of AMOC states, indicate a greenhouse gas-driven signal.

Compared to the Northward Group, the global zonal-mean precipitation, especially a notable precipitation reduction near 9°N in CESM2, and the Southward Group more closely resemble ERA5. This precipitation decrease is consistent with CESM2 sensitivity experiments with a substantial weakening of the AMOC. These results suggest that models capturing this reduction near 9°N may better represent the coupled response of the hydrological cycle to AMOC variations.

### The ongoing AMOC variations

**Fig. 5:**
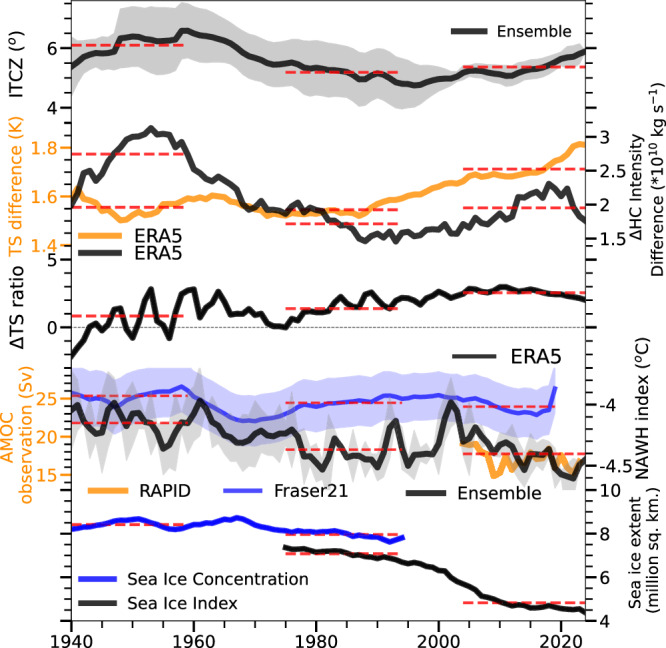
ITCZ, TS and HC intensity differences, Arctic amplification ratio, AMOC observation and proxy, the North Atlantic warming hole (NAWH) index and sea ice extent. 11-year running mean of ITCZ location ensemble derived from ERA5, JRA-55, NCEP, and ERA-20C with shading indicating one standard deviation (the first line from top to bottom), TS and HC intensity differences (the second lines), and Arctic amplification ratio (the third line) from ERA5 data. The AMOC observation at 26.5°N, AMOC proxy (Fraser21) from ref. ^61^, and the ensemble of the 11-year running mean NAWH indices from the Extended Reconstructed Sea Surface Temperature (ERSSTv5) and Hadley Center Sea Ice and Sea Surface Temperature dataset (HadISST; the fourth lines), with shading indicating one standard deviation. The 11-year running mean sea ice extent derived from sea ice concentration data (the fifth line; orange) and satellite-based observation (the fifth line; black). The red lines represent the averages over the periods 1940–1959, 1975–1994, and 2005–2024.

Because direct observations of the AMOC are limited to the post-2004 period, the long-term variability of AMOC beyond 20 years relies mostly on AMOC proxies, such as the SST contrast between the subpolar North Atlantic and the global mean^37^, sea surface salinity^55^, surface heat flux^56^, etc. These proxies are subject to substantial uncertainty and may reflect multiple physical processes beyond changes in overturning strength alone^30,56–58^. Given that AMOC weakening is one of several contributing mechanisms to the southward ITCZ movement, does the observed ITCZ shift southward imply an overall AMOC decline over the last several decades? Fig. 5 shows that all the reanalysis products suggest a southward ITCZ movement of more than 1° latitude from the 1940–60s period to the 1990s and a northward movement of about half a degree latitude in the recent 20 years (2005–2024). Since we have applied for an 11-year running filter, the interannual ITCZ variations are smoothed out. This low-frequency ITCZ variation can be induced by decadal timescale internal variability alone or a combination of internal variability and external forcing. During this period, the atmospheric CO_2_ concentration increases by about 41% (from ~300 to 422 ppmv) but is still significantly less than the CO_2_ increase in the period analyzed here (around 4 times the preindustrial level) in the 1%CO_2_ experiments. This suggests that although the trend of observed ITCZ movement could be induced by rising greenhouse gas forcing, the observed interdecadal ITCZ variations must be a combination of the external forcings and the internal variability. However, our current analysis cannot disentangle the exact percentage contribution of the different external forcings and internal variability on the observed ITCZ variability and trend in the recent 80+ years, remain uncertain, which warrants further investigation. For example, aerosol forcing is confirmed to be an important driver for the southward migration of the tropical precipitation in the late 20^th^ century^59,60^, which probably corresponds to ITCZ change from the 1940–60s period to the 1990s (Fig. 5). Nevertheless, despite the uncertainty in separating forced response from internal variability during the historical period, the observed ITCZ variations under moderate CO_2_ rise may still offer a qualitative context for understanding potential ITCZ responses under stronger future CO_2_ forcing.

Figure 5 also shows that the HC intensity contrast experiences similar changes as the ITCZ, but the TS contrast was nearly unchanged from the 1940s to 1990s and increased in the recent 20+ years, which aligns with the polar amplification measured by the ratio of polar to global mean temperatures (ΔTS ratio). This imperfect relationship between historical interdecadal ITCZ variations and TS contrast changes may reflect the complicated interactions among different external forcings and the internal variabilities in controlling this relationship. One of these processes, which includes both externally forced response and internal variability, is AMOC as suggested below.

A key indicator of AMOC variation is the North Atlantic warming hole (NAWH)^30,37^. A more negative NAWH index (“Methods”)^62^ corresponds to a cooler subpolar North Atlantic and a weaker AMOC^30,37,63^. The NAWH index undergoes an overall declining trend—trending downward over 1940s–1980s, a brief upward trend to the early 2000s, and a further downward trend afterward. Although multiple drivers could influence NAWH variations^64^, AMOC remains the most significant driver^30,31,33,38,65^. Thus, the above changes in the NAWH index suggest an overall weakening of AMOC in the last 80 years, with a brief strengthening in the early 2000s. Consistent with this interpretation, an independent AMOC proxy from ref. ^61^ also indicates a slight decline in recent decades. As we have shown in the model experiments, significant AMOC weakening leads to a southward ITCZ movement in both CESM2 and EC-Earth3. This long-term overall ITCZ southward movement in the recent 80+ years derived from multiple reanalysis products is consistent with an overall weakening of the AMOC in this period, although aerosol forcing has also been identified as a significant contributor to the southward tropical precipitation shift during the late 20^th^ century^59,60^, and the relative contributions of AMOC weakening, aerosol forcing, and internal variability cannot be definitively separated from the historical record alone.

Changes in AMOC strength can modulate meridional oceanic heat transport into the Arctic, thus influencing sea ice extent there. A stronger (weaker) AMOC generally induces a retreat (advance) of Arctic sea ice by transporting more (less) heat into the Arctic (Fig. 4, Supplementary Fig. 8)^66^. Observations and reconstructions show that Arctic sea ice experienced a modest decline from the 1950s, an accelerated loss during the 1990s–2010s, and a slower rate of decline in the most recent decade (Fig. 5). While the overall decrease in sea ice extent is commonly attributed to polar-amplified warming in association to the rising atmospheric greenhouse gas concentration, this amplification has been enhanced over the last 60+ years based on reanalysis products which cannot explain the slowdown of the Arctic sea ice loss in recent years. The slowdown of the AMOC can be one of the reasons leading to this slowdown. In addition, although the mechanism governing the formation of the NAWH is still not well understood, the continued slowdown of AMOC is one of the reasonable mechanisms leading to the enhancement of this warming hole in recent years (Fig. 5)^67,68^. Combining both reduction in sea ice loss and enhancement in the NAWH, they both point towards a weakening of AMOC at least in recent a couple of decades. Thus, the overall southward ITCZ movement shown in the reanalysis products is broadly consistent with a slowdown of the AMOC in the last few decades, though we acknowledge that aerosol forcing and internal variability likely also contribute, and a definitive attribution remains beyond the scope of this study.

## Discussion

In this study, we examine the mechanisms governing the observed and simulated shifts of the annual and zonal-mean ITCZ in response to elevated atmospheric CO₂, using ERA5, JRA-55, NCEP, ERA-20C, CMIP6 idealized 1%CO₂ experiments and idealized sensitivity experiments using CESM2 and EC-Earth3. The model responses are notably diverse, with approximately equal numbers of models projecting either a northward or southward displacement of the ITCZ. In contrast, reanalysis data indicate a pronounced southward ITCZ migration with sustained warming over 1940–2024. Sensitivity experiments using CESM2 and EC-Earth3 show that the southward ITCZ movement is dominated by AMOC weakening, and the northward ITCZ movement is dominated by greenhouse gas-induced atmospheric circulation changes. Therefore, we conclude that the ITCZ movement in response to greenhouse gas forcing is primarily due to changes in atmospheric circulation for the Northward Group and due to AMOC changes for the Southward Group. Moreover, CESM2 sensitivity experiments suggest the likely existence of three quasi-equilibrium ITCZ positions—near 6°N, the equator, and 6°S—depending on the background state and forcing scenario.

More detailed analysis reveals that when the ITCZ mean position is further away from the equator, a more pronounced AMOC weakening tends to shift it southward, consistent with the reduction in southward AHTₑ_q_, but opposite the milder enhancement in HC and TS contrast. Conversely, for an ITCZ mean position closer to the equator, an enhanced southward AHTₑ_q_ tends to drive the ITCZ northward—despite contradicting observational data—likely due to HC adjustments to Arctic amplification, while a concurrently weakened northward OHTₑ_q_ serves as a damping effect. CO_2_-induced warming without significant AMOC weakening is unable to shift the ITCZ further north in CESM2 but can in EC-Earth3. This different ITCZ response in these two models can be attributed to the control mean ITCZ location--~6°N for CESM2 (representing the Southward Group models) and ~3.5°N for EC-Earth3 (representing Northward Group models).

At the regional scale, the contributors to the global ITCZ migration differ markedly between the two groups. For the Southward Group, the global ITCZ shift is dominated by coherent southward displacements over the Pacific and Atlantic Oceans with a northward displacement in the Indian Ocean, whereas for the Northward Group, the global ITCZ migration primarily arises from changes over the Indian Ocean with minor contributions from the Atlantic and Pacific basins (Supplementary Fig. 12). For the Southward Group, AMOC weakening directly reduces the northward Atlantic OHT_eq_, thereby exerting a first-order control on the ITCZ position over the Atlantic basin and the adjacent eastern Pacific. In this regime, the AHT_eq_ associated with CO_2_ forcing plays a damping role. Consequently, the combined response of OHT_eq_ and AHT_eq_ favors a southward displacement of the regional ITCZ, and OHT_eq_ is a dominant driver of ITCZ at the global scale and within the Atlantic and Pacific basins. By contrast, owing to global ocean connectivity, anomalies in Atlantic OHT_eq_ are dynamically linked to the Indo-Pacific circulation, leading to compensatory adjustments in the meridional OHT_eq_ over the Indo-Pacific basin (Supplementary Fig. 4a, b). These oceanic adjustments interact with the interhemispheric TS contrast and induce corresponding changes in AHT_eq_. The coupled responses of OHT_eq_ and AHT_eq_ together regulate the global zonal-mean ITCZ position and promote a northward migration for the Northward Group. Thus, the zonal-mean ITCZ metric should be interpreted as an integrated indicator of hemispheric-scale energy balance rather than as evidence of uniform behavior across all longitudes. Consequently, the “tipping points” behavior identified in the zonal-mean ITCZ primarily reflects a large-scale response, while regional ITCZ adjustments may exhibit distinct magnitudes, directions, or degrees of stability across individual basins. Recognizing this distinction is essential for interpreting both the physical mechanisms and the broader implications of ITCZ shifts under climate forcing.

While our results suggest a physical linkage between AMOC weakening and southward ITCZ migration under specific forcing conditions, the relationship between AMOC strength and ITCZ position is not sufficiently consistent across different forcing scenarios and time periods to serve as a reliable proxy. The concurrent southward ITCZ migration, slowdown in Arctic sea ice decline, and intensification of the NAWH are nonetheless consistent with ongoing AMOC weakening on decadal timescales. Given the ongoing AMOC weakening and CMIP6 projections^69^ since 2005, it is plausible that the ITCZ will continue to shift further southward in the coming decades. If the AMOC were to weaken more dramatically, the ITCZ could jump to the equator and greatly alter global precipitation patterns by reorganizing the atmospheric and oceanic circulations, resulting in far-reaching impacts on the Earth’s climate.

Despite the well-known double-ITCZ biases in CMIP models, our conclusions regarding the large-scale ITCZ migration remain robust. In a comparison with the observational-based precipitation product, the Global Precipitation Climatology Project (GPCP)^70^, the Northward Group exhibits a stronger Southern Hemisphere ITCZ bias than that in the Southward Group (Supplementary Fig. 13). To quantify this difference, we decompose the zonal-mean precipitation bias into antisymmetric and symmetric components following ref. ^71^. The antisymmetric bias-which directly reflects the interhemispheric precipitation asymmetry and is most relevant to double-ITCZ bias-has an root mean square error (RMSE) of 0.293 mm day^−1^ for the Northward Group, approximately three times larger than that of the Southward Group (0.097 mm day^−1^). In contrast, the symmetric bias RMSE is nearly identical between the two groups (0.705 mm day^−1^ for the Northward Group vs. 0.697 mm day^−1^ for the Southward Group), confirming that the key difference lies in the antisymmetric (double-ITCZ) component rather than the overall precipitation magnitude. The stronger antisymmetric bias in the Northward Group, particularly over 5°S–15°S, where the mean bias reaches −1.34 mm day^−1^ compared to −0.96 mm day^−1^ in the Southward Group, could contribute to a more equatorward mean ITCZ position^72^, which may in turn influence the ITCZ response to rising greenhouse gas forcing. Nevertheless, since both groups share a similar symmetric bias structure, the contribution of the double-ITCZ bias to the sign of ITCZ migration is likely minor. Thus, the ITCZ migration is primarily controlled by the coupled AHT_eq_ and OHT_eq_ changes. Although separating the ITCZ into Northern and Southern Hemisphere branches can modify the amplitude of regional precipitation responses, it does not change the sign of the hemispheric ITCZ displacement. The ITCZ branches in the Northern and Southern Hemispheres respond coherently to changes in interhemispheric energy imbalance, reflecting a unified large-scale adjustment rather than independent regional shifts. Consequently, the zonal-mean ITCZ framework adopted here is appropriate for diagnosing the dominant mechanisms linking AMOC changes and CO_2_ forcing to global ITCZ migration, even in the presence of double-ITCZ biases.

## Methods

### Observations

ERA5 over 1940–2024^42^, JRA-55 over 1958–2023^43^, NCEP over 1948–2023^44^, and ERA-20C over 1940–2010^45^ reanalysis data are utilized as the observed atmospheric state, and both the ERSSTv5^73^ and HadISST^74^ over 1940–2024 as the observed SST. The observed Arctic sea ice extent is derived from an archive of the National Snow and Ice Data Center (NSIDC). The sea ice extent since November 1978 is based on satellite observations, but before 1978 is based on reconstruction^75^. The sea ice extent is a summation of the area where ice concentration is above 15% for satellite-based observations, but above 20% for the reconstructed data, since the increment for the latter is 10%. Here, we primarily focus on the sea ice extent trend in September, following a previous study^76^. The observed oceanic meridional heat transport is derived from the ORAS5 dataset, covering 1958–2024. The GPCP over 1979–2024^70^ is used to analyze double ITCZ biases in the climate models.

### Model simulations

To study the response of ITCZ to greenhouse gas-induced warming, we analyze the idealized 1%CO_2_ experiment from CMIP6 (see Supplementary Table 1 for the models included here). In this experiment, the CO_2_ concentration increases by 1% per year compound with a CO_2_ doubling around year 70 and quadrupling around year 140. The last 20-year mean of the idealized 1%CO_2_ experiments is used to represent the transient response of the ITCZ for a quadrupling of the CO_2_ concentration. The control run mean state is calculated as an average of a 300-year period from the preindustrial control.

To isolate the effect of AMOC weakening on ITCZ movements, we carried out a set of idealized experiments using CESM2. This set of experiments includes the standard CESM2 preindustrial control, and the idealized Thermal, Haline, ThermalHaline, 1%CO₂_fxITCZWK, and 1%CO₂_fxITCZST experiments. In the Thermal experiment, the CO_2_ concentration increases 1% per year compound for 150 years, and then the CO_2_ is kept at the level of year 150 for another 300 years in order for the surface climate to be equilibrated with the external forcing. In the Haline (or Hosing) experiment, additional freshwater is added into the subpolar North Atlantic between 50°N and 70°N with an annual increment of 0.0015 Sv (10^6^ m^3^ s^−1^) for 150 years (to 0.225 Sv), then this freshwater forcing is kept at 0.225 Sv level for another 300 years. The ThermalHaline experiment is a combination of the Thermal and Haline experiments. The last 300-year mean in these idealized experiments represents the quasi-equilibrium response of the ITCZ to the quadrupling of CO_2_ and/or AMOC collapse. In the 1%CO₂_fxITCZWK experiment, the freshwater is removed from the subpolar North Atlantic with an annual increment of −0.0035 Sv for 150 years and uniformly redistributed over 50°N and 70°N in the North Atlantic. In the 1%CO₂_fxITCZST experiment, freshwater is similarly removed at a constant annual rate of -0.0035 Sv for the first 69 years, followed by a linearly increasing removal rate—from -0.0035 Sv in year 70 to −0.0115 Sv in year 150, increasing by −0.0001 Sv per year. Compared to the Control simulation, the AMOC begins to weaken and strengthen in these two experiments around year 80, providing a basis for examining the ITCZ response. All idealized experiments were branched from the model year 500 of the CESM2 preindustrial control. For CESM2, Arctic sea ice extent is calculated based on the threshold of above 15% from the available sea ice concentration data.

To examine the dependence of ITCZ migration on its mean position and to provide a comparison with the representative model from the Southward Group, EC-Earth3 is included in this study. This choice is motivated by the fact that three closely related CMIP6 models—EC-Earth3-Veg, EC-Earth3-Veg-LR, and EC-Earth3-Aerchem—exhibit a northward ITCZ migration under CO_2_ quadrupling. EC-Earth3 was initially not included due to temporary data unavailability. In addition to the 300-year preindustrial control (Control) simulation, three sensitivity experiments are performed: fixAMOC, Hosing, and Abrupt-4 × CO_2_ using the r3i1p1f1 and r8i1p1f1 ensemble members. In the fixAMOC experiment, a salinity anomaly of 0.6 Sv is imposed north of 50°N over the Arctic and North Atlantic from 1850 to 2102 under Abrupt-4 × CO_2_ forcing to maintain a nearly constant AMOC strength. In the Hosing experiment, a uniform freshwater forcing of 0.3 Sv is applied to the polar region from 50°N to the Bering Strait during 1850–1991. In the Abrupt-4 × CO_2_ experiment, atmospheric CO_2_ is instantaneously increased to four times its preindustrial level.

### ITCZ and HC

The global and regional ITCZ location is defined as the latitude where the vertically integrated atmospheric mass streamfunction (*ψ*) over 300–700 hPa is zero^41^. The global *ψ* is defined as follows:

1 $$\psi (\,p,y)=\frac{2\pi a\cos (\,y)}{{\mbox{g}}}{\int }_{\!\!0}^{p}\bar{\nu }d{p}^{{\prime} }$$

where *a* represents the Earth radius and the $\bar{v}$ is the annual and zonal-mean meridional wind. Other symbols have their usual meaning. The strength of HC is defined as the maximum absolute value of *ψ* within each branch, with the maxima generally located in both hemispheres.

The regional *ψ* is defined as follows:

2 $$\psi (\,p,y)=\frac{2\pi a\cos (\,y)}{g}{\int }_{\!\!0}^{p}{\bar{v}}_{D}{\mbox{d}}{p}^{{\prime} }$$

Here ${\bar{v}}_{D}$ is the divergent component of the annual and zonal-mean meridional wind over the Indian Ocean (20°E–70°E), Pacific Ocean (70°E–120°W) and Atlantic Ocean (120°W–20°E) sections, following the previous regional division^77^.

### AMOC

AMOC intensity is defined as the maximum of the Atlantic meridional streamfunction below 500 m depth between 40°N and 60°N^78^.

### TS difference

For ERA5 data and CMIP6 models, the TS difference is calculated as the area-weighted temperature contrast between northern tropics (0°–30°N) and southern tropics (0°–30°S). For these reanalysis data, the change in TS difference is quantified as the difference between the last and the first 30-year periods, such as ERA5 (1995–2024 and 1940–1969), JRA-55 (1994–2023 and 1958–1987), NCEP (1994–2023 and 1948–1977), and ERA-20C (1981–2010 and 1940–1969). For the CMIP6 models, the change is defined as the difference between the last 20-year mean of the 1%CO_2_ experiments and the Control simulation.

### AHT and OHT

The meridional global AHT is calculated by integrating the net heat flux into the atmosphere northward at a given latitude (*θ*) from the south pole as follows^79^:

3 $${\mbox{AHT}}(\theta )=2\pi {a}^{2}{\int }_{\!\!-\frac{\pi }{2}}^{\theta }({Q}_{{TOA}}-{Q}_{S})\cos {\theta }^{{\prime} }{\mbox{d}}{\theta }^{{\prime} }$$

where *Q*_*TOA*_ and *Q*_*S*_ represent the net incoming radiation at the top of the atmosphere (TOA) and the net heat flux (radiation plus turbulent) at the surface, respectively. *a* is the radius of the Earth. The method used to calculate AHT changes from ERA5 reanalysis and CMIP6 climate models is similar to that used for the interhemispheric TS contrast.

The historical meridional OHT is calculated using annual mean ocean temperature (***T***(*x*,*y*,*z*,*t*)) and meridional velocity (***v***(*x*,*y*,*z*,*t*)) fields from the ORAS5 dataset, covering 1958–2024. The calculation is as follows:

4 $${\mbox{OHT}}(y,t)=\iint \widetilde{{{{\boldsymbol{v}}}}}\,{{{\boldsymbol{T}}}}{\mbox{d}}x{\mbox{d}}z\times {\mbox{cp}}\times {\rho }_{0}$$

where $\widetilde{{{{\boldsymbol{v}}}}}$ = ***v***-v*, and the section-averaged velocity of each latitude section v* is calculated as ${{{{\boldsymbol{v}}}}}^{*}=\iint {{{\boldsymbol{v}}}}(x,y,z,t){\mbox{d}}x{\mbox{d}}z/A$, in which *A* stands for the area of each section at specific latitude. The specific heat capacity cp and the mean density of sea water ${\rho }_{0}$ are set to 4000 J kg^−1^ K^−1^ and 1026 kg m^−3^, respectively. Meridional OHT is computed for the global, Atlantic, and Indo-Pacific basins. The Atlantic section spans 75°W–20°E, while the Indo-Pacific section covers 20°E–75°W. Following ref. ^80^, the northernmost latitude for OHT is capped at 65°N, and the southernmost latitude for regional OHT is 34°S. For CMIP6 models, some OHT fields are available directly from the variable “hfbasin” in the preindustrial and 1%CO_2_ simulations. For other models, the OHT is calculated based on their temperature and velocity outputs (Supplementary Table 1). The OHT change from ORAS5 is defined as the difference between the last and first 30 years of the 1958-2024 period, while the change in the climate models follows the same approach for the TS contrast.

### Arctic amplification index

Arctic amplification refers to the amplified Arctic warming compared to global warming, reflecting a feature of climate change. The Arctic amplification index is defined as the area-weighted ratio of surface temperature change in the Arctic (60°–90°N) to that of the global mean. ERA5 data from 1940 to 2024 is used for this calculation.

### AMOC observation

The RAPID/Meridional Overturning Circulation and Heatflux Array/Western Boundary Time Series moored array (RAPID)^81^ is used to obtain the observed time series of the AMOC at around 26.5°N from April 2004 to February 2023.

### NAWH index

NAWH index is defined as the difference of the average SST between the subpolar North Atlantic (45°–65°N, 50°–20°W) and the broader extratropical North Atlantic (30°–65°N, 70°–10°W) using both ERSSTv5 and HadISST. This has been proven to be able to capture the local SST deviation relative to the North Atlantic^62^.

## Supplementary information

Supplementary Information

Transparent Peer Review file

## Data Availability

ERA5 data are available at https://cds.climate.copernicus.eu/datasets/reanalysis-era5-pressure-levels-monthly-means?tab=download. ERSSTv5 data are available at https://www.ncei.noaa.gov/pub/data/cmb/ersst/v5/netcdf/. HadISST data are available at https://www.metoffice.gov.uk/hadobs/hadisst/data/download.html. NSIDC data are available at https://nsidc.org/data/g02135/versions/3 and https://nsidc.org/data/g00799/versions/1. ORAS5 data are available at https://cds.climate.copernicus.eu/datasets/reanalysis-oras5?tab=download. RAPID observations are available at https://rapid.ac.uk/data/data-download. CMIP6 model data are available at https://esg-dn1.nsc.liu.se/search/cmip6-liu/. The minimum CESM2 and EC-Earth3 data generated in this study have been deposited in the Zenodo repository under accession code DOI: 10.5281/zenodo.19906006 and are publicly available at https://zenodo.org/records/19906006.

## Code availability

The Python code for generating Figs. 1–5 in this study has been deposited in the Zenodo repository under accession code DOI: 10.5281/zenodo.19906173 and are publicly available at https://zenodo.org/records/19906173.
