# Supplementary material for: Migration of the intertropical convergence zone driven by ocean circulation changes
Source: Nat Commun. 2026 May 16;17:6501. doi: 10.1038/s41467-026-73200-2 (PMC13377125; doi:10.1038/s41467-026-73200-2)
Supplement: Supplementary file 1 — Supplementary Information [file 41467_2026_73200_MOESM1_ESM.pdf]

Supplementary Materials for

Migration of the Intertropical Convergence Zone Driven by Ocean Circulation Changes

Yaru Guo[1,2], Aixue Hu[1*], Gerald A. Meehl[1], Maria J. Molina[1,3], Hui Li[1], Katinka Bellomo[4], and Nan Rosenbloom[1]

[1]National Center for Atmospheric Research, Boulder, CO, USA

[2]Key Laboratory of Ocean Observation and Forecasting and Key Laboratory of Ocean Circulation and Waves, Institute of Oceanology, Chinese Academy of Sciences, Qingdao 266071, China

[3]University of Maryland, College Park, MD, USA

[4]Department of Geosciences, University of Padova, Padova, Italy

*Corresponding author: ahu@ucar.edu

This PDF file includes:

Supplementary Table 1

Supplementary Figure 1 to Figure 13

Supplementary Table 1. The Coupled Model Intercomparison Project Phase 6 (CMIP6) models used in this study. The meridional ocean heat transport for each model is either directly obtained from the variable '*hfbasin*' or calculated using meridional velocity (*V*) and ocean temperature (*T*).

**Model code**	**Model name**	**Institute**	**Member**	**OHT source**
M01	ACCESS-CM2	CSIRO	r1i1p1f1	*V*/*T*
M02	ACCESS-ESM1-5	CSIRO	r1i1p1f1	*V*/*T*
M03	CanESM5	CCCma	r1i1p1f1	*hfbasin*
M04	CanESM5-1	CCCma	r1i1p1f1	*hfbasin*
M05	CanESM5-CanOE	CCCma	r1i1p2f1	*hfbasin*
M06	CESM2	NCAR	r1i1p1f1	*hfbasin*
M07	CMCC-CM2-SR5	CMCC	r1i1p1f1	*hfbasin*
M08	CMCC-ESM2	CMCC	r1i1p1f1	*hfbasin*
M09	CNRM-CM6-1	CNRM-CERFACS	r1i1p1f2	*V*/*T*
M10	CNRM-ESM2-1	CNRM-CERFACS	r1i1p1f2	*V*/*T*
M11	E3SM-1-0	E3SM-Project	r1i1p1f1	*V*/*T*
M12	EC-Earth3-AerChem	EC-Earth-Consortium	r1i1p1f1	*hfbasin*
M13	EC-Earth3-Veg	EC-Earth-Consortium	r1i1p1f1	*hfbasin*
M14	EC-Earth3-Veg-LR	EC-Earth-Consortium	r1i1p1f1	*hfbasin*
M15	FGOALS-g3	CAS	r1i1p1f1	*hfbasin*
M16	GFDL-ESM4	NOAA-GFDL	r1i1p1f1	*hfbasin*
M17	GISS-E2-1-G	NASA-GISS	r1i1p1f1	*hfbasin*
M18	HadGEM3-GC31-LL	MOHC	r1i1p1f3	*hfbasin*
M19	HadGEM3-GC31-MM	MOHC	r1i1p1f3	*hfbasin*
M20	INM-CM4-8	INM	r1i1p1f1	*V*/*T*
M21	INM-CM5-0	INM	r1i1p1f1	*V*/*T*
M22	IPSL-CM6A-LR	IPSL	r1i1p1f1	*V*/*T*
M23	IPSL-CM6A-MR1	IPSL	r1i1p1f1	*hfbasin*
M24	MIROC6	MIROC	r1i1p1f1	*V*/*T*
M25	MIROC-ES2L	MIROC	r1i1p1f2	*V*/*T*
M26	MPI-ESM1-2-HR	MPI-M	r1i1p1f1	*hfbasin*
M27	MPI-ESM1-2-LR	MPI-M	r1i1p1f1	*hfbasin*
M28	MPI-ESM-1-2-HAM	HAMMOZ-Consortium	r1i1p1f1	*hfbasin*
M29	MRI-ESM2-0	MRI	r1i1p1f1	*V*/*T*
M30	NorCPM1	NCC	r1i1p1f1	*hfbasin*
M31	NorESM2-LM	NCC	r1i1p1f1	*hfbasin*
M32	NorESM2-MM	NCC	r1i1p1f1	*hfbasin*
M33	SAM0-UNICON	SNU	r1i1p1f1	*V*/*T*
M34	UKESM1-0-LL	MOHC	r1i1p1f2	*hfbasin*
M35	UKESM1-1-LL	MOHC	r1i1p1f2	*hfbasin*

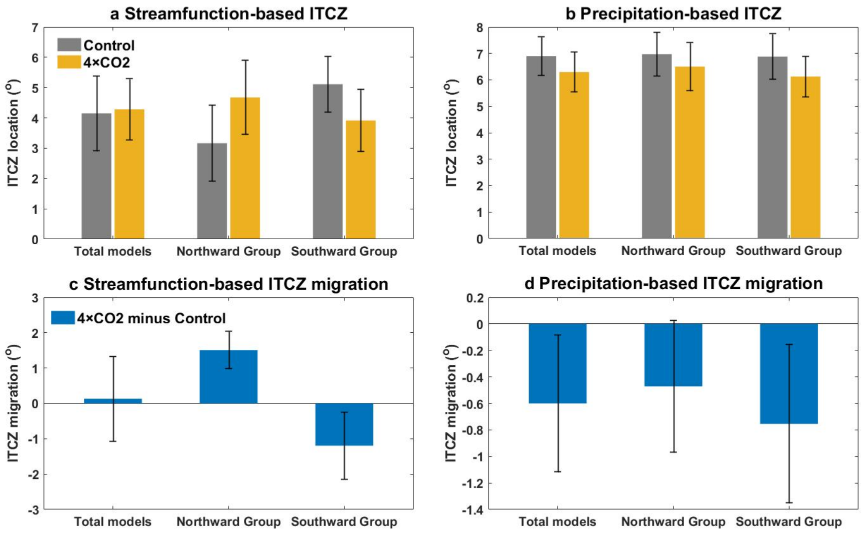

Supplementary Figure 1 | The Intertropical Convergence Zone (ITCZ) position and migration based on streamfunction- and precipitation-based definitions. **a**, Ensemble-mean global zonal-mean ITCZ position from all models, as well as from the Northward and Southward Groups. Error bars indicate one standard deviation for each model group. The ITCZ position is defined as the latitude where the vertically integrated atmospheric mass streamfunction (ψ) over 300-700 hPa is zero[41]. **b** Same as **a**, but with the ITCZ defined by the latitude of maximum precipitation[48]. **c-d**, Same as **a-b**, but showing ITCZ migration from the preindustrial control (hereafter "Control") to the CO_2 quadrupling, defined as the mean over years 131-150, when atmospheric CO_2 reaches approximately four times its preindustrial level.

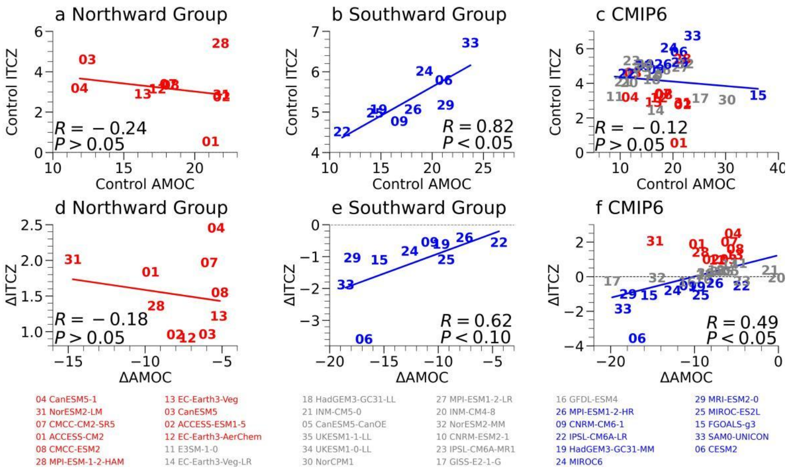

Supplementary Figure 2 | Relationships between the Atlantic Meridional Overturning Circulation (AMOC) and the ITCZ across CMIP6 models. a-c, Intermodel relationships between the climatological mean AMOC strength and ITCZ position for the Northward Group (**a**), Southward Group (**b**), and all CMIP6 models (**c**). **d-f**, Same as **a-c**, but showing the relationship between AMOC weakening and ITCZ migration between CO_2 quadrupling (mean over years 131-150 of the 1%CO_2 experiment) and the preindustrial control simulation. For each panel, the Pearson correlation coefficient (**R**) and the **P** value from a linear regression (black solid line) are shown. The FGOALS-g3 model (label 15) is excluded as an outlier in panels **b-c** (climatological mean AMOC and ITCZ position), but is included in panels **e-f** (AMOC weakening and ITCZ migration).

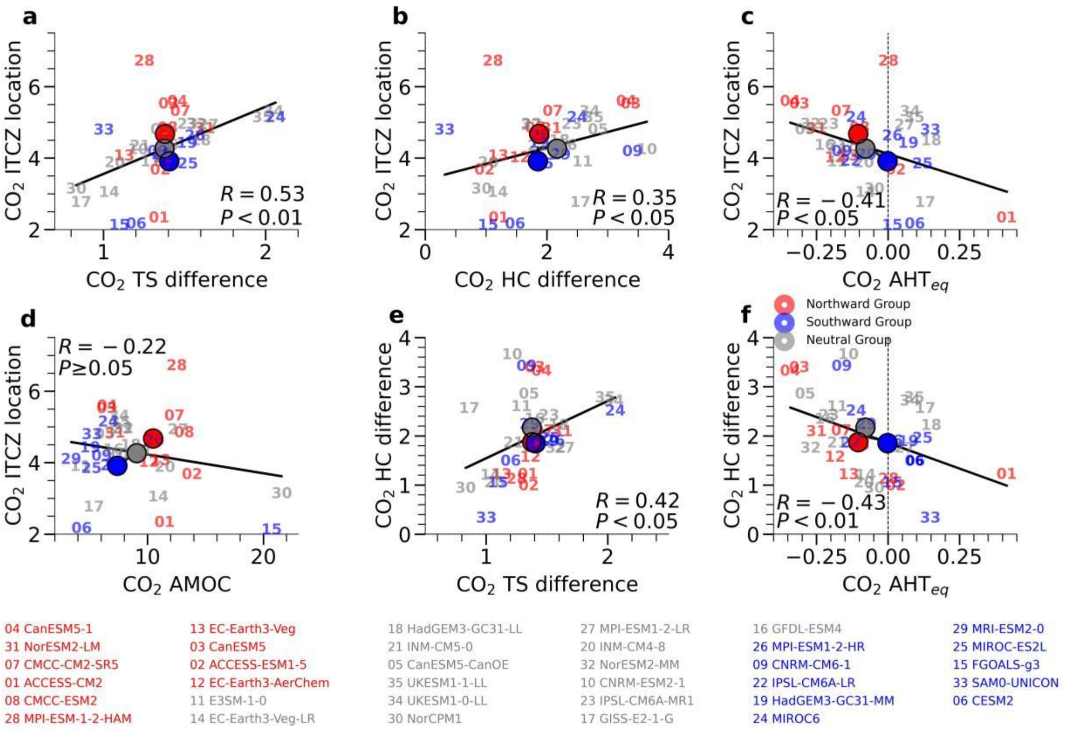

Supplementary Figure 3 | Relationships under CO_2 quadrupling conditions (mean over years 131-150). a-d, Relationships across CMIP6 models between ITCZ location and: the tropical surface temperature (TS) difference between 0°–30°N and 0°–30°S (**a**), hemispheric Hadley Cell (HC) intensity difference (**b**), the northward cross-equatorial atmospheric heat transport (AHT_{eq}) (**c**), and AMOC strength (**d**). **e, f,** Relationships between HC intensity difference and: TS difference (**e**) and northward AHT_{eq} (**f**). The correlation coefficient (**R**) and **P** value for a linear fit (black solid line) are shown. The Northward, Southward, and Neutral Group ensembles are shown.

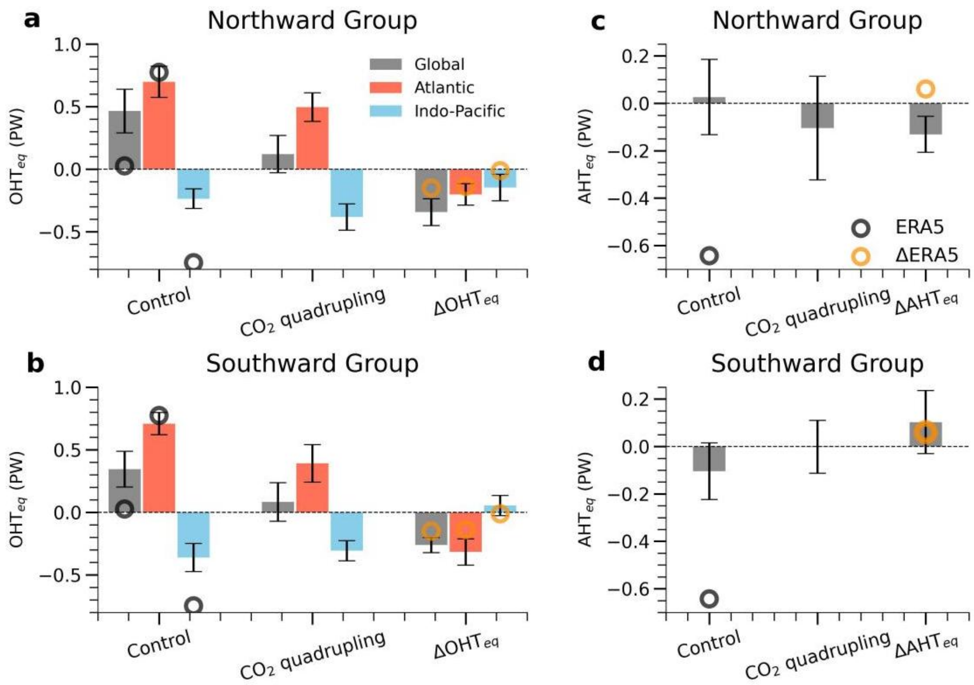

Supplementary Figure 4 | Northward cross-equatorial oceanic heat transport (OHT_{eq}) and AHT_{eq}. **a,** Northward OHT_{eq} for the global (gray), Atlantic (orange), and Indo-Pacific (blue) basins in the Control simulation, CO_2 quadrupling, and their changes (CO_2 quadrupling minus Control), for the Northward Group. **b,** Same as **a,** but for Southward Group. **c, d,** Same as **a, b,** but for global AHT_{eq}. Error bars indicate one standard deviation for each model group. The black circle represents the observed mean: OHT_{eq} from Ocean Reanalysis System 5 (ORAS5;1958–2024) or AHT_{eq} from the fifth generation European Centre for Medium-Range Weather Forecasts (ECMWF) reanalysis (ERA5; 1940–2024). The orange circle shows observed changes, defined as the difference between the last and first 30 years of each respective period. Changes in global northward OHT_{eq} are the -0.34 ± 0.09 PW for the Northward Group and –0.25 ± 0.06 PW for the Southward Group. Global northward OHT_{eq} in the ORAS5 is 0.03 PW over 1958-2024, 0.11 PW over 1958-1987, and -0.04 PW over 1994-2024. Global northward AHT_{eq} in the ERA5 is –0.64 PW over 1940-2024, -0.67 PW over 1940-1969, and -0.61 PW over 1994-2024. Changes in global AHT_{eq} are -0.13±0.08 PW for the Northward Group and +0.10±0.13 PW for the Southward Group.

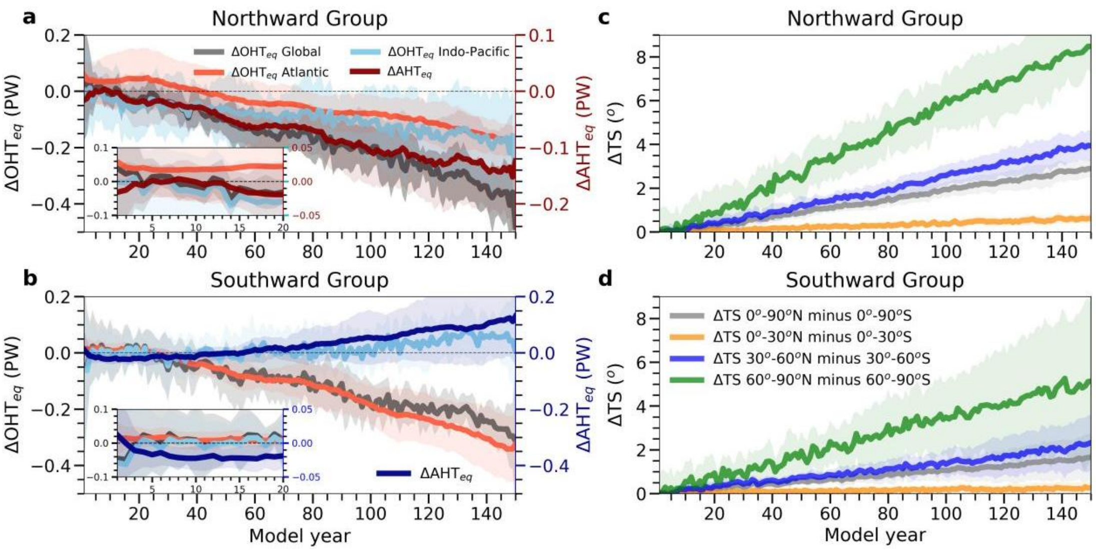

Supplementary Figure 5 | Changes in OHT_{eq}, AHT_{eq}, and TS. a, (left axis) Ensemble changes in the 11-year running mean northward OHT_{eq} in the global (gray), Atlantic (orange), and Indo-Pacific (blue) basins from the 1%CO_2 experiment of the climate models in the Northward Group, relative to their respective Control simulations. **a**, (right axis) Same as left axis, but for global AHT_{eq}. **b,** Same as **a,** but for the Southward Group. **c,** Ensemble changes in the interhemispheric TS difference across latitude bands—global (0°-90°, gray), low-latitude (0°-30°, orange), mid-latitude (30°-60°, blue), and high-latitude (60°-90°, green), derived from the 1%CO_2 experiment of the climate models in the Northward Group, relative to the Control simulations. **d,** Same as **c,** but for the Southward Group. Shading indicates one standard deviation for each group.

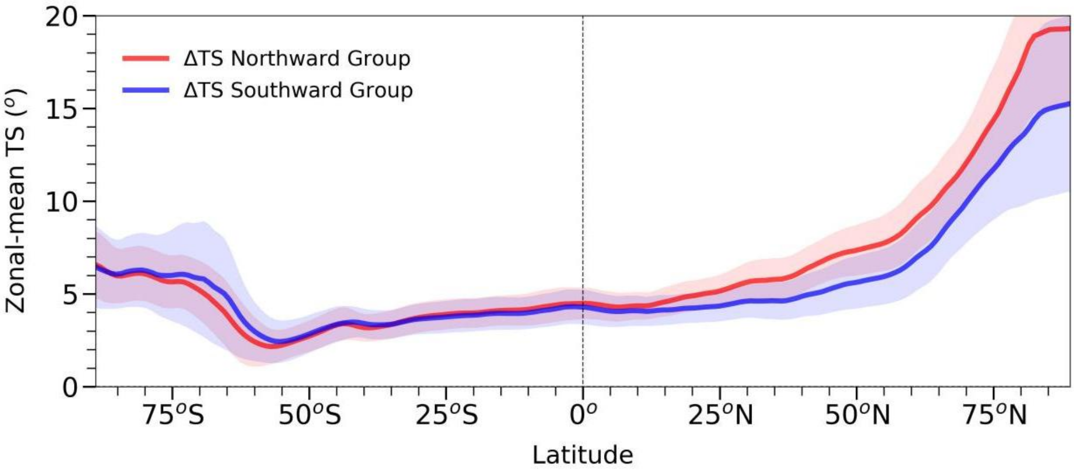

Supplementary Figure 6 | Zonal-mean TS change. a, Ensemble zonal-mean TS changes from the 1%CO_2 experiment relative to their respective Control for the Northward (red) and Southward (blue) Groups. Shading indicates one standard deviation for each group.

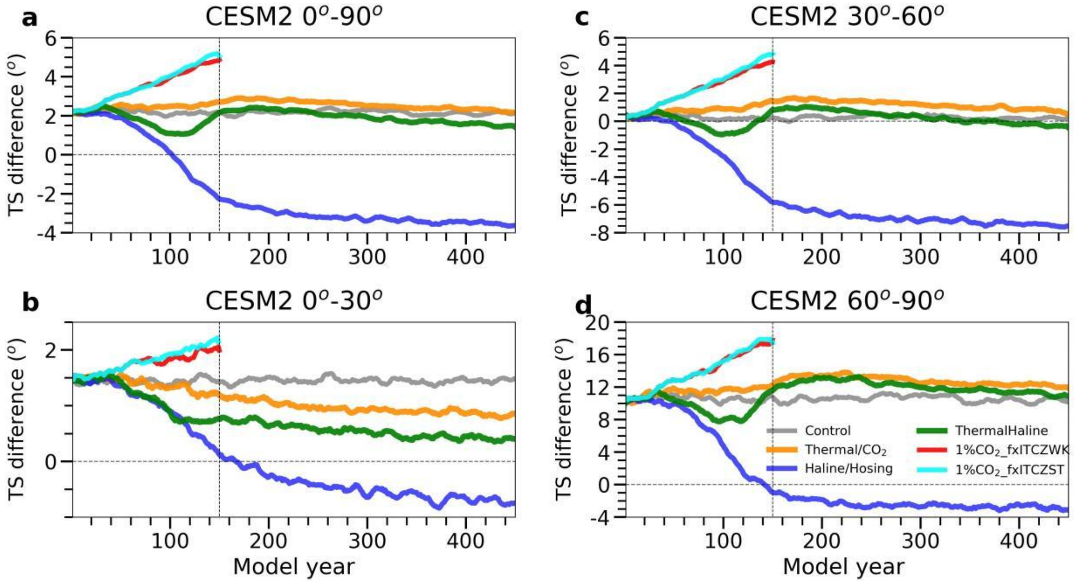

Supplementary Figure 7 | Hemispheric TS difference in the Community Earth System Model version 2 (CESM2) simulations. TS difference between the Northern and Southern Hemispheres across **a,** hemispheric (0°-90°), **b,** low-latitude (0°-30°), **c,** mid-latitude (30°-60°), and **d,** high-latitude (60°-90°) bands, derived from CESM2 Control (gray), Thermal/CO_2 (orange), Haline/Hosing (blue), ThermalHaline (green), 1%CO_2_fxITCZWK (red), and 1%CO_2_fxITCZST (cyan) simulations.

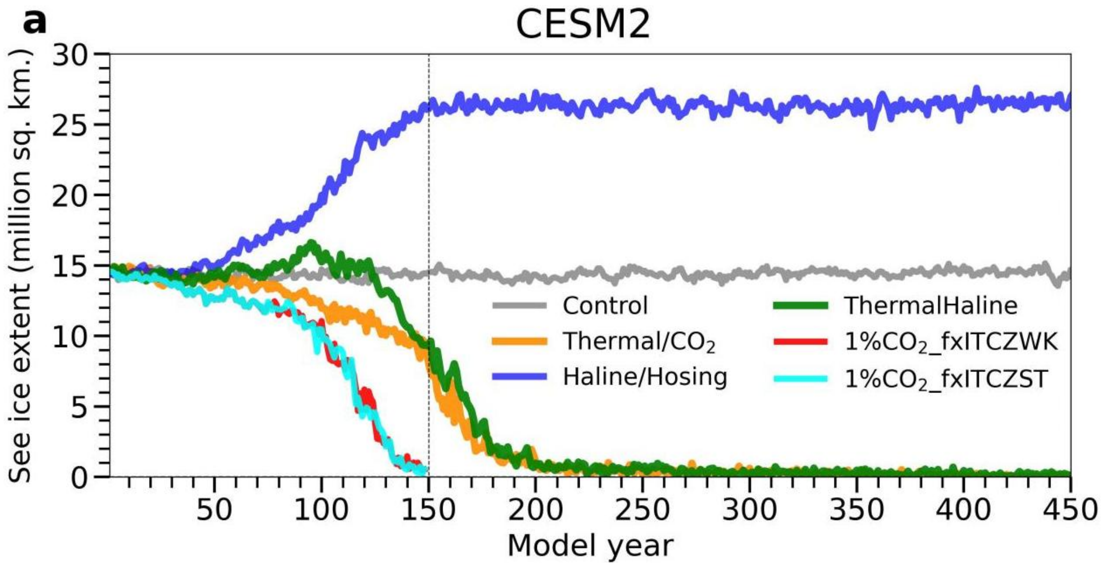

Supplementary Figure 8 | Arctic sea ice extent in CESM2 simulations. Arctic sea ice extent from CESM2 Control (gray), Thermal/CO_2 (orange), Haline/Hosing (blue), ThermalHaline (green), 1%CO_2_fxITCZWK (red), and 1%CO_2_fxITCZST (cyan) simulations.

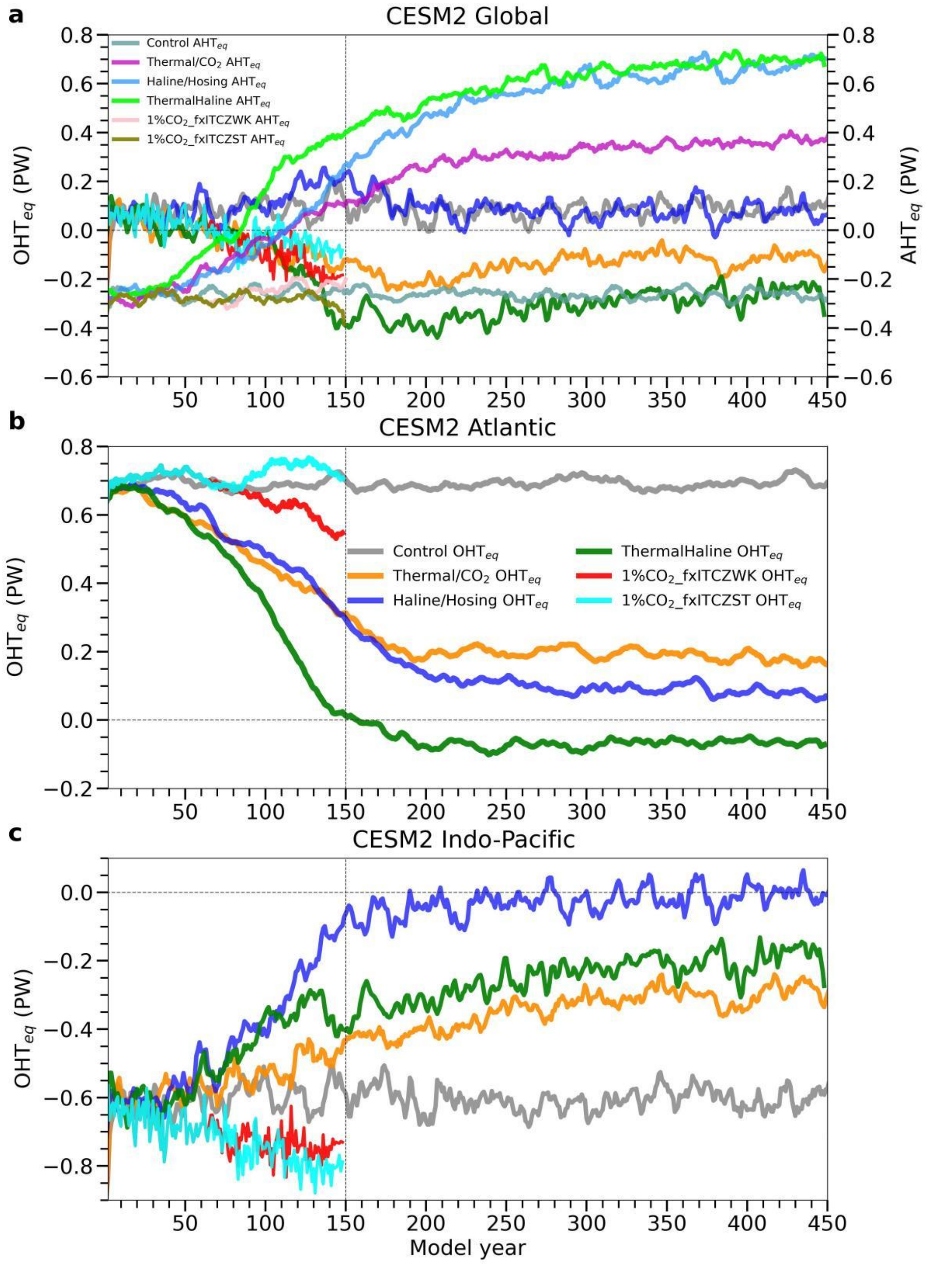

Supplementary Figure 9 | Northward OTH_{eq} and AHT_{eq} in CESM2 simulations. a, (left axis) The 11-year running mean of global northward OHT_{eq} from CESM2 Control (gray), Thermal/CO_2 (orange), Haline/Hosing (blue), ThermalHaline (green), and 1%CO_2_fxITCZWK (red), and 1%CO_2_fxITCZST (cyan) simulations. (right axis) Same as left axis, but for global northward AHT_{eq}. **b, c,** Same as **a** (left axis)**,** but for the Atlantic (**b**) and Indo-Pacific (**c**) basins.

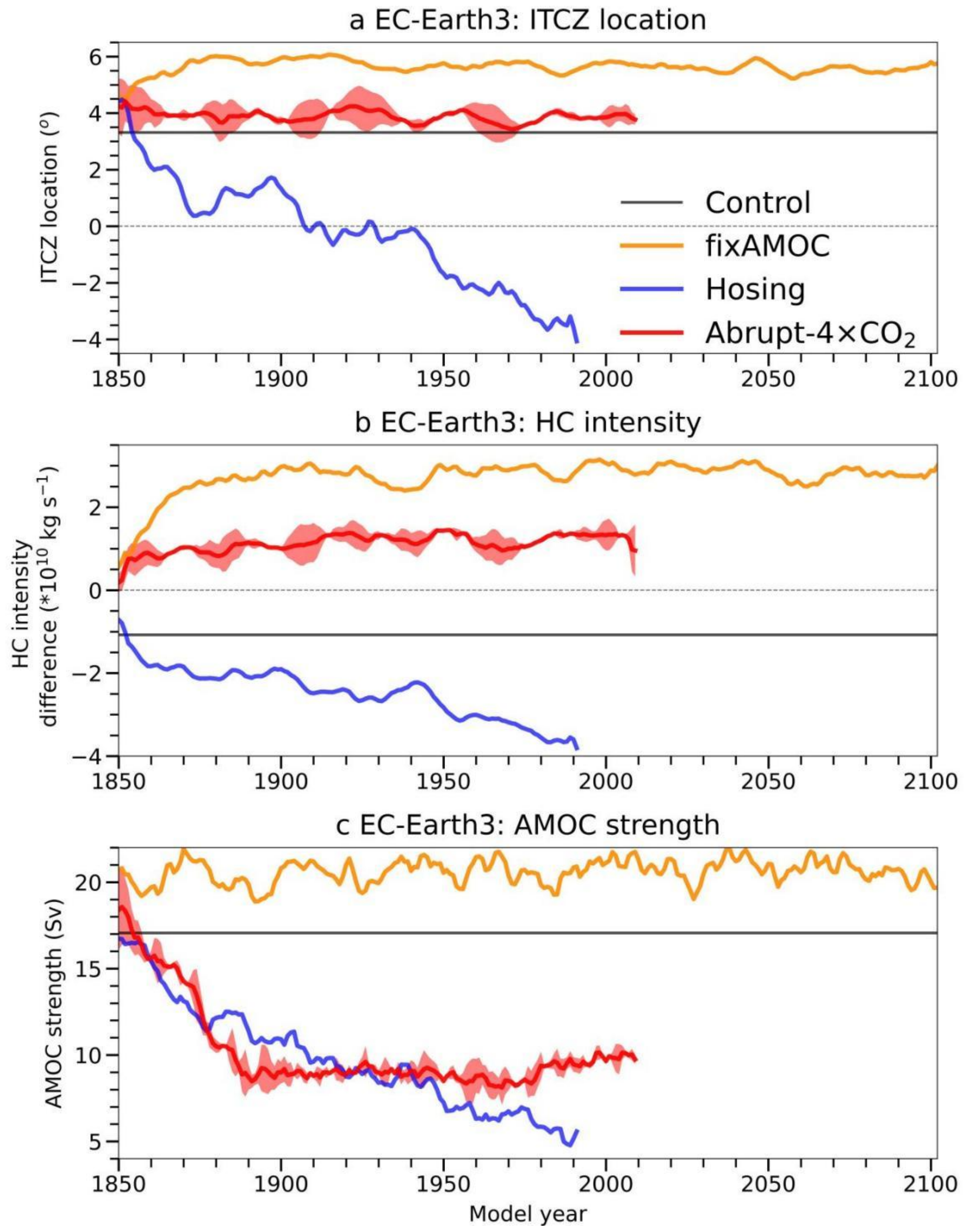

Supplementary Figure 10 | ITCZ, HC intensity difference, and AMOC intensity. a, The 11-year running mean ITCZ location (**a**), HC intensity difference (**b**), AMOC intensity (**c**) from the EC-Earth3 simulations, including the 300-year preindustrial control mean (gray), fixAMOC (orange), Hosing (blue), and Abrupt-4×CO_2 experiments. The Abrupt-4×CO_2 curve shows the ensemble mean of the r3i1p1f1 and r8i1p1f1 members, with shading indicating one standard deviation.

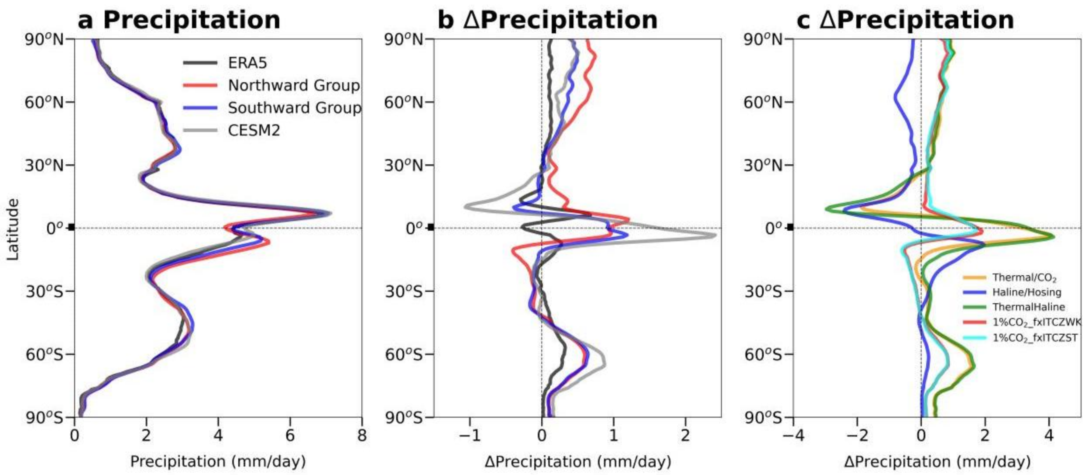

Supplementary Figure 11 | Zonal mean precipitation and anomalies. a Climatological zonal-mean precipitation for 1940-2024 from ERA5 (black line), and from the preindustrial control simulations of the Northward Group (red line), Southward Group (blue line), and CESM2 Control (gray line). **b** Same as **a**, but for the precipitation anomalies: the difference between the last and first 30 years in ERA5, and the difference between the CO_2 quadrupling and Control simulations for the Northward Group, Southward Group, and CESM2. **c** Precipitation anomalies from CESM2 sensitivity experiments relative to its Control simulation.

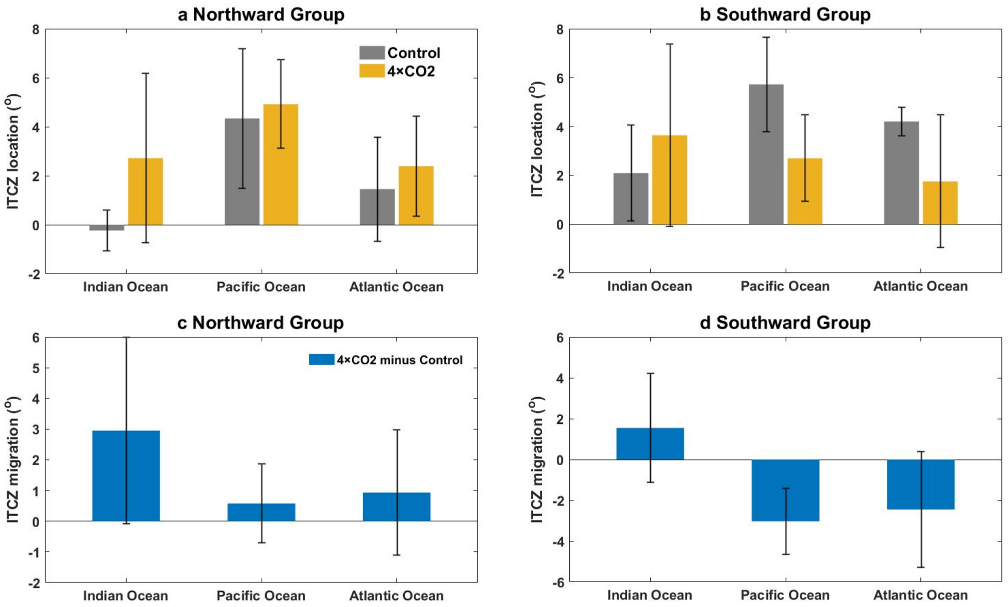

Supplementary Figure 12 | ITCZ location and migration across ocean basins. a, Ensemble-mean climatological ITCZ position in the preindustrial control (gray) and during years 131-150 of the CMIP6 1%CO_2 experiment (yellow), shown separately for the Indian Ocean (20°E-105°E), Pacific Ocean (105°E-85°W) and Atlantic Ocean (85°W-20°E) sections for the Northward Group. Error bars denote one standard deviation across models within each group. **b** Same as **a**, but for the Southward Group. **c-d** Same as **a-b**, but showing ITCZ migration, defined as the change in ITCZ position from the preindustrial climatology to the CO_2 quadrupled state.

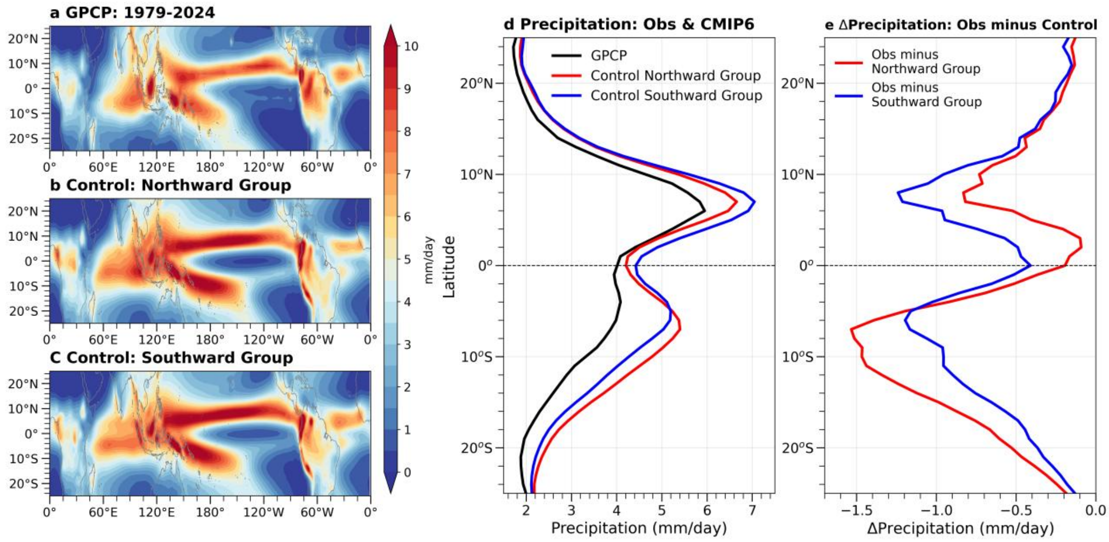

Supplementary Figure 13 | Climatological precipitation distribution and zonal-mean structure. a-c, Climatological precipitation from the Global Precipitation Climatology Project (GPCP) over 1979-2024 (**a**), and the ensemble-mean Control precipitation from the Northward (**b**) and Southward (**c**) Groups. **d**, Global zonal-mean precipitation from GPCP and the Control simulations of the Northward and Southward Groups. **e**, Zonal-mean precipitation biases, defined as the difference between the GPCP climatology and the Control simulations of the Northward and Southward Groups.
