# Supplementary material for: Migration of the intertropical convergence zone driven by ocean circulation changes
Source: Nat Commun. 2026 May 16;17:6501. doi: 10.1038/s41467-026-73200-2 (PMC13377125; doi:10.1038/s41467-026-73200-2)
Supplement: Supplementary file 2 — Transparent Peer Review file [file 41467_2026_73200_MOESM2_ESM.pdf]

nature portfolio

Peer Review File

Migration of the Intertropical Convergence Zone Driven by Ocean Circulation Changes

Corresponding Author: Dr Aixue Hu

This file contains all reviewer reports in order by version, followed by all author rebuttals in order by version.

Attachments originally included by the reviewers as part of their assessment can be found at the end of this file.

Version 0:

Reviewer comments:

Reviewer #1

(Remarks to the Author)
Review of "Intertropical Convergence Zone at Risk of Crossing a Tipping Point Driven by Ocean Circulation Changes in a Warming Climate"

By Guo et al.,

Overview:
This manuscript investigates the mechanisms governing past and future migrations of the Intertropical Convergence Zone (ITCZ) using ERA5 reanalysis, CMIP6 1%CO_2 simulations, and a suite of CESM2 sensitivity experiments designed to disentangle the roles of atmospheric and oceanic processes—particularly the Atlantic Meridional Overturning Circulation (AMOC). The authors argue that (1) only CMIP6 models with a more northern mean ITCZ position reproduce the observed southward ITCZ shift, (2) this southward migration is primarily driven by substantial AMOC weakening rather than greenhouse-gas-driven warming alone, and (3) the ITCZ exhibits multiple quasi-stable equilibria (near 6°N, the equator, and 6°S) depending on AMOC state. The authors further propose that recent observed ITCZ shifts reflect a long-term decline in AMOC.
The manuscript presents an ambitious and potentially impactful contribution to climate dynamics. The analyses draw on multiple coordinated modelling experiments and a large suite of observational products, and the results address long-standing uncertainties about the coupled ocean–atmosphere control of the ITCZ position. However, several key issues regarding robustness, interpretation, and presentation must be addressed.
Here are my major comments on the manuscript:
1. Global ITCZ versus regional ITCZ: Is the calculation of ITCZ position and intensity in the present study representing the global ITCZ rather than the regional ITCZ? I am wondering whether the Pacific ITCZ or the Atlantic ITCZ dominates the change in global ITCZ. It is straightforward to understand how the changes in AMOC could influence the Atlantic ITCZ. But how the changes in AMOC could influence the Pacific ITCZ requires a physical explanation.
2. The double ITCZ biases in CMIP models: the CMIP models exhibit strong double ITCZ biases. How do the model biases influence the robustness of the conclusions in the present study? Especially, the ITCZ calculations do not separate the Northern Hemisphere ITCZ and the Southern Hemisphere ITCZ. Does the "North Group" show stronger ITCZ biases in the Southern Hemisphere? In the other way around, does the "South Group" show less SH ITCZ biases? If the calculations in the present study are separated into SH ITCZ and NH ITCZ, how will this influence the conclusions?
3. Lines 22-24 and 62-63, the major conclusion of the study, as "These findings imply that the observed southward ITCZ migration over recent decades may signal a marked weakening of the AMOC, with far-reaching climate implications." I am not convinced by these conclusions, and I do not see strong evidence to support such conclusions. As most of the analysis in the study, we can see that even with strong atmospheric CO2 forcing, the main drivers of ITCZ are surface temperature difference, Hadley circulation, and atmospheric heat transport. The ocean heat transport and the change in AMOC are not major drivers of the global ITCZ. Within the historical period, the external forcing is much weaker than the CO2 quadrupling. I do not find any evidence to support such a strong statement.
4. Lines 127-131: Where can I see all the results? Which figures or analyses are showing the results here? When you say "more moderate increases", what do you mean by that? Compared to what?
5. Lines 132-134: Does that mean we should see more pronounced ITCZ changes in the Atlantic than the Pacific ITCZ? If

the OHT is a dominant driver of ITCZ. Please explain how your results support your description here.
6. The AMOC change, as shown in Fig. 3 in the South Group, is in line with the No Group. This raises the question of whether AMOC could be a robust driver of ITCZ.
7. Lines 189-191 about the tipping point, discuss thereafter: what is your definition of tipping point? Is it an abrupt change, or should it reflect transitions of two stable states? If it is the latter one, are your simulations sufficient to demonstrate stable states, or is what is shown in the present study just transient behavior?
8. Lines 284 -285 and lines 286-295: see my review point 3. I do not agree with the statement to use observed southward ITCZ movement as an AMOC change proxy. At least, I don't think the authors show any evidence of that in the current analysis. And in lines 293-295, the Fig. 5 shows a decline in AMOC in 2010. Where do you see AMOC strengthening? The discussions in this paragraph contradict each other. I don't see how you can link AMOC weakening or strengthening to the Arctic sea ice loss.

Reviewer #2

(Remarks to the Author)
Review of the paper: Intertropical Convergence Zone at Risk of Crossing a Tipping Point Driven by Ocean Circulation Changes in a Warming Climate (Guo et al.)

Summary

This manuscript is a strong and timely contribution to the literature on tropical climate dynamics. It advances understanding in three key ways.
(1) It demonstrates that AMOC weakening is the essential mechanism needed to reconcile observed (ERA5) ITCZ behaviour with CMIP6 model responses. Only models with a more northern ITCZ mean position and a strong AMOC decline reproduce the observed southward migration; warming alone cannot displace the ITCZ farther north once it already resides in a northern state.
(2) It introduces a compelling ITCZ tipping-point framework, showing that the ITCZ can occupy multiple quasi-stable equilibrium positions depending on the state and forcing of the AMOC. This highlights that ITCZ shifts are not indefinitely gradual but may become locked into distinct dynamical regimes.
(3) It proposes observed ITCZ migration as a new independent proxy for long-term AMOC variability, a valuable insight given that direct AMOC observations span only about two decades.
Collectively, these contributions clarify model–observation inconsistencies, deepen theoretical understanding, and point to practical early-warning indicators for large-scale ocean–atmosphere reorganisations.

Major Issues

While the manuscript is strong, several important caveats should be more explicitly acknowledged and addressed:
(1) Dependence on ERA5 for small ITCZ shifts. The central linkage between ITCZ migration and AMOC weakening relies heavily on ERA5's ability to accurately resolve subtle latitudinal displacements. Since these variations are small and reanalysis products contain structural uncertainty, the robustness of the observational constraint has not been fully quantified.
(2) Ambiguity in attributing multidecadal AMOC variability. Although the observed ITCZ behaviour is consistent with AMOC weakening, attribution remains plausible but not definitive. AMOC variations prior to 2004 are poorly constrained, and proxy-based reconstructions are influenced by multiple drivers. The manuscript would benefit from clarifying the level of confidence associated with this inference.
(3) Use of a zonal-mean ITCZ metric. By diagnosing the ITCZ primarily through its zonal-mean latitude, the study may obscure substantial longitudinal asymmetries and regionally important rainfall responses. This limitation is particularly relevant when discussing "tipping points" and quasi-stable states, which may manifest differently across individual ocean basins.

Minor Comments

(1) The phrase "southward ITCZ migration of –0.61°" is redundant; southward migration already implies a negative sign. Consider rephrasing for clarity.
(2) You may wish to cite the following paper to highlight known challenges in determining ITCZ position across different diagnostics: Ramotubei, T.S., Landman, W.A., Mateyisi, M.J., Nangombe, S., Beraki, A.F. (2025). Response of the global ITCZ to different ENSO phases and how the ITCZ determined from the maximum precipitation compares with the surface tropical winds convergence. Environmental Development, 55, 101210. This work underscores the sensitivity of ITCZ identification to the chosen metric.

Reviewer #3

(Remarks to the Author)
See attached file

[Editorial Note: See end of file]

Version 1:

Reviewer comments:

Reviewer #1

(Remarks to the Author)
The authors' extensive new analyses satisfactorily address my prior concerns and provide compelling evidence for the robustness of the main conclusions. The manuscript has been substantially strengthened through revision. I have no further comments and recommend acceptance in its current form.

Reviewer #2

(Remarks to the Author)
The authors have made a substantial effort to address the reviewer comments, and the manuscript has improved significantly in clarity, structure, and physical interpretation.

Importantly, my primary concern regarding the strength of the AMOC-related conclusions has been adequately addressed. The revised manuscript now presents a more balanced and appropriately cautious interpretation, clearly distinguishing between consistency and causality.

The additional analyses on basin-scale ITCZ behaviour and the expanded discussion of mechanisms further strengthen the study.

Some minor concerns remain regarding the treatment of model biases (e.g., double-ITCZ) and the extent to which their influence has been fully quantified. However, these do not undermine the main conclusions.

Overall, I consider the manuscript suitable for publication after minor revision.

Reviewer #3

(Remarks to the Author)
See attached file

[Editorial Note: See end of file]

Open Access This Peer Review File is licensed under a Creative Commons Attribution 4.0 International License, which permits use, sharing, adaptation, distribution and reproduction in any medium or format, as long as you give appropriate

credit to the original author(s) and the source, provide a link to the Creative Commons license, and indicate if changes were made.
In cases where reviewers are anonymous, credit should be given to 'Anonymous Referee' and the source.
The images or other third party material in this Peer Review File are included in the article's Creative Commons license, unless indicated otherwise in a credit line to the material. If material is not included in the article's Creative Commons license and your intended use is not permitted by statutory regulation or exceeds the permitted use, you will need to obtain permission directly from the copyright holder.
To view a copy of this license, visit https://creativecommons.org/licenses/by/4.0/

● Reviewer #1

We sincerely thank the reviewers for their thorough, constructive, and insightful comments, which have helped us to significantly improve the manuscript. In response to these suggestions, we have carefully revised both the analysis and the presentation of the results.

A brief summary of the major revisions is provided below.

Specifically, we have undertaken a comprehensive revision of the text, figures, and discussion to (i) explicitly distinguish between global zonal-mean and regional ITCZ behavior through basin-scale diagnostics; (ii) assess the influence of double-ITCZ biases on the robustness of our conclusions using observational comparisons; and (iii) substantially revise the interpretation linking observed ITCZ migration to AMOC variability, framing such connections as physically consistent but not definitive. In addition, we have expanded the physical discussion of how coupled oceanic and atmospheric heat transport adjustments regulate ITCZ migration, clarified the conditional role of AMOC weakening relative to atmospheric circulation responses and the background ITCZ state, and carefully refined the definition and usage of the term "tipping point." Several new analyses and Extended Data figures have been added, and corresponding revisions have been incorporated throughout the manuscript.

We hope that the reviewers will agree with us that these revisions have adequately addressed the reviewers' concerns and the robustness and physical consistency of this work have been substantially strengthened. A detailed, point-by-point response is provided below.

Reviewer #1 (Remarks to the Author):
Review of "Intertropical Convergence Zone at Risk of Crossing a Tipping Point Driven by Ocean Circulation Changes in a Warming Climate"
By Guo et al.,
Overview:
This manuscript investigates the mechanisms governing past and future migrations of the Intertropical Convergence Zone (ITCZ) using ERA5 reanalysis, CMIP6 1%CO_2 simulations, and a suite of CESM2 sensitivity experiments designed to disentangle the roles of atmospheric and oceanic processes—particularly the Atlantic Meridional Overturning Circulation (AMOC). The authors argue that (1) only CMIP6 models with a more northern mean ITCZ position reproduce the observed southward ITCZ shift, (2) this southward migration is primarily driven by substantial AMOC weakening rather than greenhouse-gas-driven warming alone, and (3) the ITCZ exhibits multiple quasi-stable equilibria (near 6°N, the equator, and 6°S) depending on AMOC state. The authors further propose that recent observed ITCZ shifts reflect a long-term decline in AMOC.
The manuscript presents an ambitious and potentially impactful contribution to climate dynamics. The analyses draw on multiple coordinated modelling experiments and a large suite of observational products, and the results address long-standing uncertainties about the coupled ocean–atmosphere control of the ITCZ position. However, several key issues

regarding robustness, interpretation, and presentation must be addressed.
Here are my major comments on the manuscript:
1. Global ITCZ versus regional ITCZ: Is the calculation of ITCZ position and intensity in the present study representing the global ITCZ rather than the regional ITCZ? I am wondering whether the Pacific ITCZ or the Atlantic ITCZ dominates the change in global ITCZ. It is straightforward to understand how the changes in AMOC could influence the Atlantic ITCZ. But how the changes in AMOC could influence the Pacific ITCZ requires a physical explanation.

Response: Thank you for this insightful comment. We clarify that the ITCZ position and intensity analyzed in the main text represent the global zonal-mean ITCZ, rather than basin-specific ITCZs. For a more comprehensive review of the ITCZ, we have done additional diagnoses on regional ITCZ migrations over the Indian, Pacific, and Atlantic Oceans using the same streamfunction-based metric (Extended Data Fig. 12).
The regional decomposition reveals that for the Southward Group, the global ITCZ migration is dominated by coherent southward shifts over the Pacific and Atlantic Oceans. In contrast, for the Northward Group, the global ITCZ shift primarily results from changes over the Indian Ocean, while the Pacific and Atlantic contributions are comparatively small. These results demonstrate that both the Pacific and Atlantic ITCZs can exert substantial control on the global zonal-mean ITCZ only in the Southward Group.
As noted by the reviewer, the influence of AMOC changes on the Atlantic ITCZ is relatively straightforward: AMOC weakening reduces northward Atlantic ocean heat transport (OHT), alters the interhemispheric energy balance, and shifts the ITCZ southward. However, the AMOC impact is not confined to the Atlantic basin. Due to global ocean connectivity, changes in Atlantic OHT are accompanied by compensating adjustments in the meridional OHT over the Indo-Pacific basin, as shown in Extended Data Fig. 4a, b. The coupled responses of OHT_{eq} and AHT_{eq} together regulate the global ITCZ position, thereby influencing the Pacific ITCZ indirectly through dynamically consistent ocean-atmosphere coupling. In particular, under CO_2 forcing, variations in AHT_{eq} play a more prominent role in controlling ITCZ changes for the Northward Group, while OHT_{eq} exerts a stronger influence through the Atlantic and Pacific ITCZs for the Southward Group. We have added a detailed discussion in the revised manuscript to explicitly clarify how AMOC weakening can affect the Pacific ITCZ through these indirect, yet physically consistent, global energy-transport adjustments (Lines 477-494).

"At the regional scale, the contributors to the global ITCZ migration differ markedly between the two groups. For the Southward Group, the global ITCZ shift is dominated by coherent southward displacements over the Pacific and Atlantic Oceans with a northward displacement in the Indian Ocean, whereas for the Northward Group, the global ITCZ migration primarily arises from changes over the Indian Ocean with minor contributions from the Atlantic and Pacific basins (Extended Data Fig. 12). For the Southward Group, AMOC weakening directly reduces the northward Atlantic OHTeq, thereby exerting a first-order control on the ITCZ position over the Atlantic basin and the adjacent eastern Pacific. In this regime, the AHT_{eq} associated with CO_2 forcing plays a damping role. Consequently, the combined response of OHT_{eq} and AHT_{eq} favors a southward displacement of the regional ITCZ,

and OHT_{eq} is a dominant driver of ITCZ at the global scale and within the Atlantic and Pacific basins. By contrast, owing to global ocean connectivity, anomalies in Atlantic OHT_{eq} are dynamically linked to the Indo-Pacific circulation, leading to compensatory adjustments in the meridional OHT_{eq} over the Indo-Pacific basin (Extended Data Fig. 4a, b). These oceanic adjustments interact with the interhemispheric TS contrast and induce corresponding changes in AHT_{eq}. The coupled responses of OHT_{eq} and AHT_{eq} together regulate the global zonal-mean ITCZ position and promote a northward migration for the Northward Group. Thus, the zonal-mean ITCZ metric should be interpreted as an integrated indicator of hemispheric-scale energy balance rather than as evidence of uniform behavior across all longitudes."

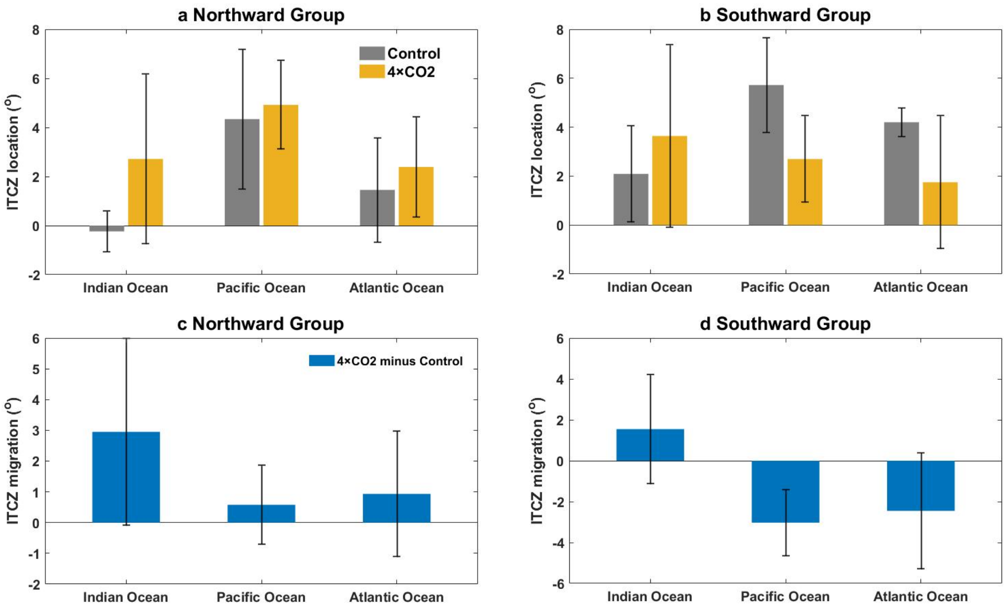

Extended Data Fig. 12 | ITCZ location and migration across ocean basins. a, Ensemble-mean climatological ITCZ position in the preindustrial control (gray) and during years 131-150 of the CMIP6 1%CO_2 experiment (yellow), shown separately for the Indian Ocean (20°E-105°E), Pacific Ocean (105°E-85°W) and Atlantic Ocean (85°W-20°E) sections for the Northward Group. Error bars denote one standard deviation across models within each group. **b** Same as **a**, but for the Southward Group. **c-d** Same as **a-b**, but showing ITCZ migration, defined as the change in ITCZ position from the preindustrial climatology to the CO_2 quadrupled state.

2. The double ITCZ biases in CMIP models: the CMIP models exhibit strong double ITCZ biases. How do the model biases influence the robustness of the conclusions in the present study? Especially, the ITCZ calculations do not separate the Northern Hemisphere ITCZ and the Southern Hemisphere ITCZ. Does the "North Group" show stronger ITCZ biases in the Southern Hemisphere? In the other way around, does the "South Group" show less SH ITCZ biases? If the calculations in the present study are separated into SH ITCZ and NH ITCZ, how will this influence the conclusions?

Response: Thank you for this thoughtful comment. This is a good question. We fully acknowledge the presence of pronounced double-ITCZ biases in CMIP models. To assess how

these biases may affect the robustness of our conclusions, we compare the climatological precipitation distributions from the Control simulations of the Northward and Southward Groups with observations using the 2.5° × 2.5° monthly Global Precipitation Climatology Project (GPCP) dataset for 1979-2024 (Extended Data Fig. 13).

This comparison shows that the Northward Group does exhibit a slightly stronger ITCZ bias in the Southern Hemisphere than the Southern Group, consistent with a bit more pronounced double-ITCZ structure, but with a weaker Northern ITCZ bias compared to the Southern Group. Potentially the larger double ITCZ in the Northward Group could be a reason causing a northward ITCZ movement under elevated greenhouse gas forcing, which warrants further details study, but it is beyond the scope of this study. More importantly, although we could not clearly isolate how much this double ITCZ bias has contributed to the ITCZ's response to the increased greenhouse gases, it is clear that the existence of the different levels of this double ITCZ bias in these two groups of models is not the fundamental reason for their different ITCZ movement in responding to the same forcing changes. Therefore, this will not change the conclusions reached here, at least not qualitatively.

Because our focus in this study is on the zonal- and annual-mean annual ITCZ, the existence of the double ITCZ bias will not affect our results since the Northern ITCZ is still the stronger one in all models, and the use of an atmospheric circulation based ITCZ definition (the intersection of the zonal- and annual-mean Hadley Cells) will always identify the movement of the stronger ITCZ branch (for us, it is the northern branch). The Northern and Southern Hemisphere ITCZ branches respond coherently to changes in interhemispheric energy balance controlled by coupled AHT_{eq} and OHT_{eq} adjustments. Therefore, the zonal- and annual-mean ITCZ provides a robust metric for diagnosing the large-scale ITCZ response to CO_2 forcing and AMOC changes, despite the presence of double-ITCZ biases in individual models. In the revised text, the discussion related to the influence of the model ITCZ biases has been added (Lines 506-524).

"*Despite the well-known double-ITCZ biases in CMIP models, our conclusions regarding the large-scale ITCZ migration remain robust. In a comparison with the observational-based precipitation product, the Global Precipitation Climatology Project (GPCP)[68], the Northward Group exhibits a stronger Southern Hemisphere ITCZ bias than that in the Southward Group (Extended Data Fig. 13). This could be a reason for a mean ITCZ location closer to equator in the Northward Group[69] which could have contributed the response of the ITCZ to the rising greenhouse gas forcing. For example, the precipitation reduction in the southern ITCZ zone in the Northward Group (Extended Data Fig. 11b) can be one of the reasons to lead to a northward ITCZ movement because a smaller southern ITCZ bias position makes the northern ITCZ further away from the equator (a northward movement). Therefore, the climatological mean-state biases could have contributed to the ITCZ migration, especially in the Northward Group. Since both groups have the double ITCZ bias, the contribution of this bias to the ITCZ movement should be minor. Thus, the ITCZ migration is primarily controlled by the coupled AHT_{eq} and OHT_{eq} changes. Although separating the ITCZ into Northern and Southern Hemisphere branches can modify the amplitude of regional precipitation responses, it does not change the sign of the hemispheric ITCZ displacement. The ITCZ branches in the Northern and Southern Hemispheres respond coherently to changes in interhemispheric energy*

imbalance, reflecting a unified large-scale adjustment rather than independent regional shifts. Consequently, the zonal-mean ITCZ framework adopted here is appropriate for diagnosing the dominant mechanisms linking AMOC changes and CO_2 forcing to global ITCZ migration, even in the presence of double-ITCZ biases."

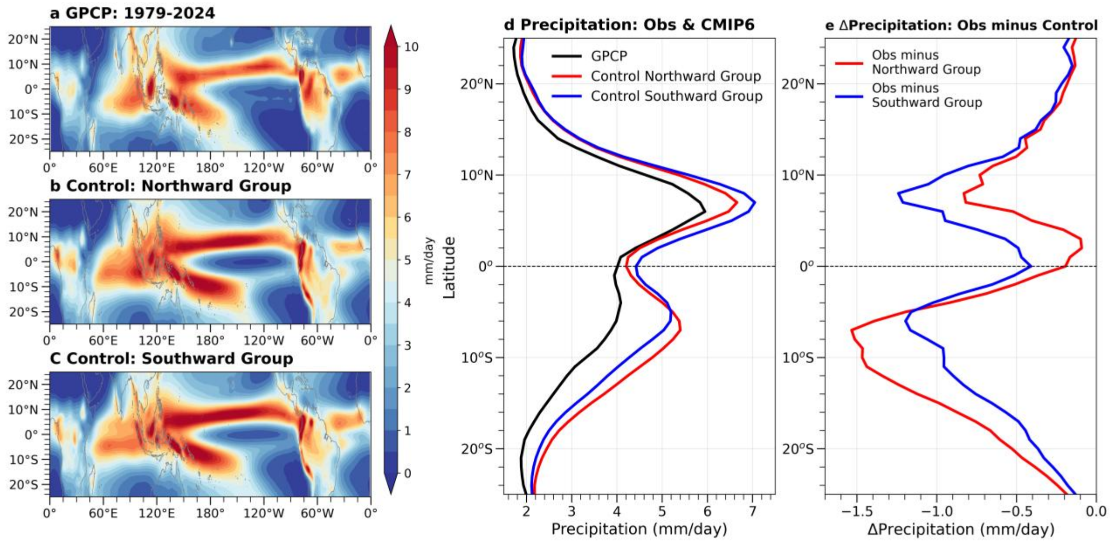

Extended Data Fig. 13 | Climatological precipitation distribution and zonal-mean structure. **a-c**, Climatological precipitation from GPCP over 1979-2024 (**a**), and the ensemble-mean Control precipitation from the Northward (**b**) and Southward (**c**) Groups. **d**, Global zonal-mean precipitation from GPCP and the Control simulations of the *Northward* and *Southward Groups*. **e**, Zonal-mean precipitation biases, defined as the difference between the GPCP climatology and the Control simulations of the *Northward* and *Southward Groups*.

3. Lines 22-24 and 62-63, the major conclusion of the study, as "These findings imply that the observed southward ITCZ migration over recent decades may signal a marked weakening of the AMOC, with far-reaching climate implications." I am not convinced by these conclusions, and I do not see strong evidence to support such conclusions. As most of the analysis in the study, we can see that even with strong atmospheric CO2 forcing, the main drivers of ITCZ are surface temperature difference, Hadley circulation, and atmospheric heat transport. The ocean heat transport and the change in AMOC are not major drivers of the global ITCZ. Within the historical period, the external forcing is much weaker than the CO2 quadrupling. I do not find any evidence to support such a strong statement.

Response: Thank you for this important and constructive comment. We agree that the attribution between the observed ITCZ migration inferred from ERA5 and changes in the AMOC over recent decades involves substantial uncertainty. Although the ITCZ behavior in our study is consistent with a weakened AMOC, we emphasize that this relationship should be regarded as plausible rather than definitive.

Furthermore, we adopted another AMOC proxy (Fig. 5) and cited more references to support the present conclusion that the AMOC weakening may be ongoing over the past decades. Specifically, direct observations of the AMOC are limited to the post-2004 RAPID array, and earlier AMOC change is primarily inferred from proxy records or indirect diagnostics, which

remain imperfectly constrained (Fu et al., 2018). Existing reconstructions provide mixed evidence regarding long-term AMOC changes. Several proxy-based studies suggest a weakening of the AMOC since the late nineteenth or twentieth century (e.g., Rahmstorf et al., 2015; Thornalley et al., 2018), whereas more recent estimates based on air-sea heat fluxes indicate no significant long-term decline since the 1960s (Terhaar et al., 2025). Our results are in line with an earlier study of Bryden et al. (Nature, 2005) who showed an AMOC weakening trend since 1950s based on hydrological cross sections. Moreover, AMOC proxies can be influenced by multiple physical processes beyond overturning strength alone, further complicating attribution. In light of these uncertainties, we have revised the text to explicitly acknowledge that AMOC weakening represents one potential contributing mechanism to the observed ITCZ southward shift. We now more clearly state the limitations of available AMOC reconstructions and frame our interpretation within a probabilistic and hypothesis-consistent context (Lines 25-27, 85-86, 392-398, 431-437, 499-505).

In addition, the primary objective of this study is to investigate ITCZ behavior under controlled forcing using preindustrial control and 1%CO_2 experiments, and to place the ITCZ changes during recent decades (as inferred from ERA5 over 1940-1959, 1975-1994, and 2005-2024) into a physically consistent framework. A key result of our analysis is that, for models with a relatively northern mean ITCZ position, a southward ITCZ shift requires a pronounced weakening of the AMOC, whereas CO_2-induced atmospheric adjustments alone are insufficient to produce such a migration. This conclusion is drawn from the consistency in ITCZ mean state, variability, and migration behavior between reanalysis (ERA5, JRA-55, NCEP, and ERA-20C) and the Southward Group models.

"These findings imply that the observed southward ITCZ migration over recent decades is consistent with a weakened AMOC, within the context of coupled atmosphere-ocean interactions."

"Under this framework, the observed southward ITCZ migration in recent decades is consistent with, but does not uniquely imply, an ongoing weakening of the AMOC."

"Because direct observations of the AMOC are limited to the post-2004 period, the long-term variability of AMOC beyond 20 years relies mostly on AMOC proxies, such as the sea surface temperature (SST) contrast between subpolar North Atlantic and the global mean[37], sea surface salinity[55], surface heat flux[56] etc. These proxies are subject to substantial uncertainty and may reflect multiple physical processes beyond changes in overturning strength alone[30,56-58]. Given that AMOC weakening is one of several contributing mechanisms to the southward ITCZ movement, does the observed ITCZ shift southward imply an overall AMOC decline over the last several decades?"

"Consistent with this interpretation, an independent AMOC proxy from ref 59 also indicates a slight decline in recent decades. As we have shown in the model experiments, significant AMOC weakening leads to a southward ITCZ movement in both CESM2 and EC-Earth3. This long-term overall ITCZ southward movement in the recent 80+ years derived from multiple reanalysis products could be a sign of overall weakening of the AMOC in this period.

Although a more definitive demonstration of this relationship for observations still needs more work, we give the following indirect evidence."

"We further propose that the observed southward ITCZ migration in the reanalysis products could serve as a proxy for long-term AMOC weakening (decadal or longer timescales), supported by the concurrent slowdown in Arctic sea ice decline and intensification of the NAWH. Given the ongoing AMOC weakening and CMIP6 projections[67] since 2005, it is plausible that the ITCZ will continue to shift further southward in the coming decades. If the AMOC would weaken more dramatically, the ITCZ could jump to the equator and greatly alter global precipitation patterns by reorganizing the atmospheric and oceanic circulations, resulting in far reaching impacts on the Earth's climate."

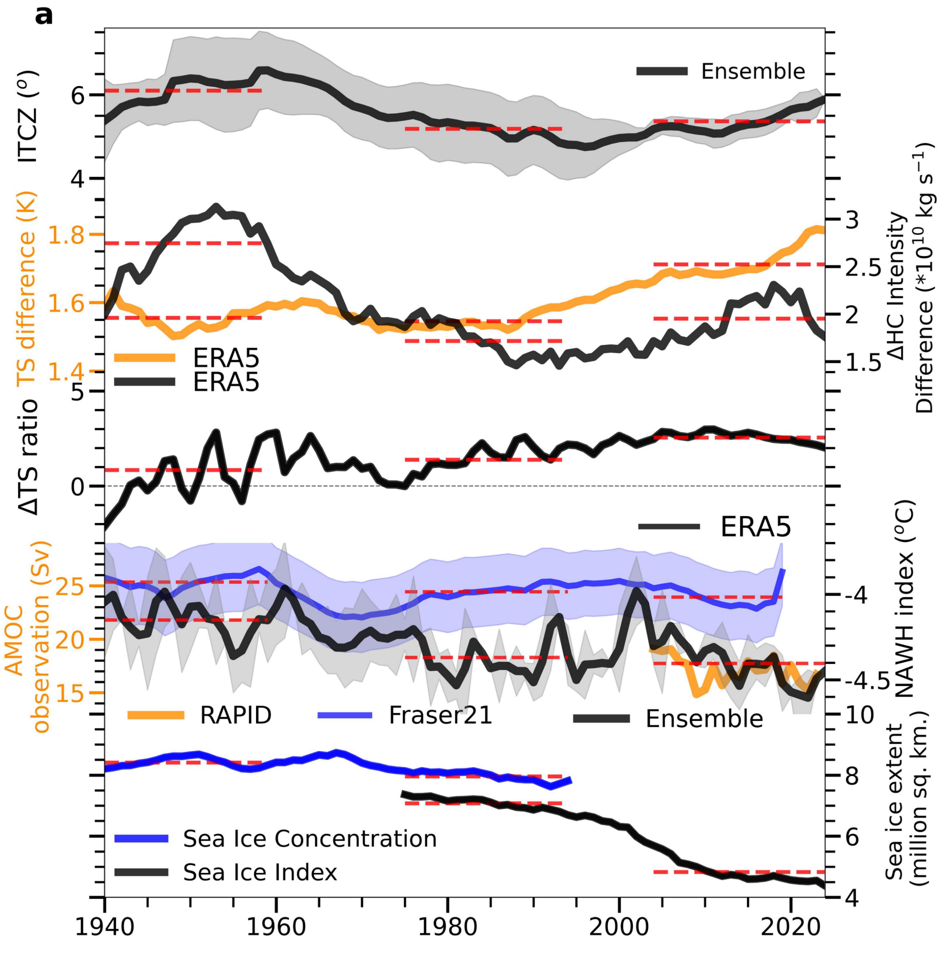

Fig. 5 | ITCZ, TS and HC intensity differences, Arctic amplification ratio, AMOC observation and proxy, NAWH index, and sea ice extent. 11-year running mean of ITCZ location ensemble derived from ERA5, JRA-55, NCEP, and ERA-20C (the first line from top to bottom), TS and HC intensity differences (the second lines), and Arctic amplification ratio (the third line) from ERA5 data. The AMOC observation at 26.5oN, AMOC proxy (Fraser21) from ref 59, and the ensemble of the 11-year running mean NAWH indices from the Extended Reconstructed Sea Surface Temperature (ERSSTv5) and Hadley Centre Sea Ice and Sea Surface Temperature dataset (HadISST; the fourth lines), with shading indicating one standard deviation. The 11-year running mean sea ice extent derived from sea ice

concentration data (the fifth line; orange) and satellite-based observation (the fifth line; black). The red lines represent the averages over the periods 1940-1959, 1975-1994, and 2005-2024.

4. Lines 127-131: Where can I see all the results? Which figures or analyses are showing the results here? When you say “more moderate increases”, what do you mean by that? Compared to what?

Response: Thank you for pointing this out. We agree that the description in Lines 127-131 was insufficiently clear. The results referred to in this paragraph are shown primarily in Fig. 3 and Extended Data Fig. 3. We have now explicitly cited these figures in the revised text.

By “more moderate increases”, we mean that the increases in interhemispheric TS contrast, HC intensity and the southward AHT_{eq} in the Southward Group are smaller than those in the Northward Group. To avoid ambiguity, we have added the corresponding quantitative values in the revised main text to clarify this comparison. Specifically, we have expanded this paragraph to clearly describe how changes in atmospheric and oceanic circulations compete to determine the final ITCZ migration in each group, with quantitative values and explicit figure references added (Lines 198-215):

“To investigate the physical processes governing ITCZ migration under CO_2 quadrupling, we examine changes in both atmospheric and oceanic circulations. In the Northward Group, there is a consistent increase in interhemispheric TS contrast (+0.57 $\pm$0.20°C; Fig. 3a), HC intensity contrast (+2.03 $\pm$ 0.55 $\times$ 10^{10} kg s^{-1}; Fig. 3b), and southward AHT_{eq} (− 0.13 $\pm$ 0.07 PW; Fig. 3c), which support a northward ITCZ migration. Although the global northward OHT_{eq} reduces by 74.01% (or -0.34 $\pm$ 0.09 PW; Extended Data Fig. 4a) which tends to shift the ITCZ southward, the overall northward ITCZ movement in this model group indicates that the oceanic circulation only partially offsets the dominant atmospheric contribution to the northward ITCZ migration in the Northward Group. In contrast, in the Southward Group, the increases in interhemispheric TS (+0.24 $\pm$ 0.26°C; Fig. 3a) and HC intensity contrasts (+0.63 $\pm$ 0.74 $\times$ 10^{10} kg s^{-1}; Fig. 3b) are more moderate (less than 50% of the changes in the Northward Group) which tends to move ITCZ northward. However, the southward AHT_{eq} reduces by +0.10 $\pm$ 0.13 PW; Fig. 3c) which favors a southward ITCZ movement. The inconsistency between TS/HC contrast and southward AHT_{eq} indicates that the southward AHT_{eq} is controlled not only by the changes in atmospheric circulation and temperature, but also the moisture changes. Moreover, the substantially weakened northward OHT_{eq} (by 75.96% or − 0.25 $\pm$ 0.06 PW; Extended Data Fig. 4b) acts to shift the ITCZ southward. Since changes in OHT_{eq} are much larger than AHT_{eq}, OHT_{eq} dominates the ITCZ movement southward in the Southward Group. The enhancement in HC and TS contrasts is a result of a stronger AMOC decline, as illustrated next.”

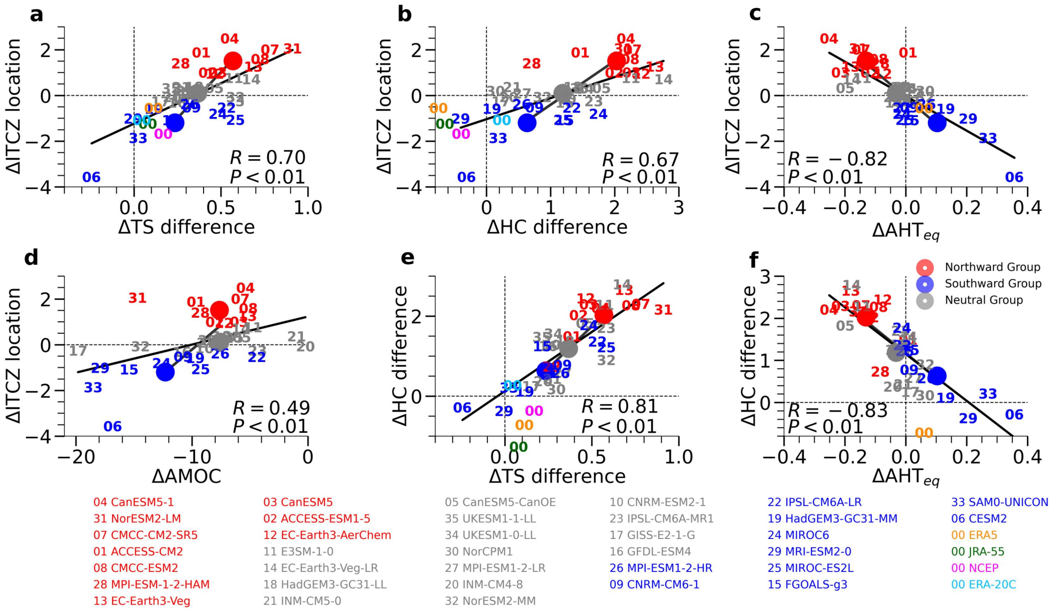

Fig. 3 | Same as Fig. 2, but showing changes between CO_2 quadrupling (mean over years 131-150 of 1%CO_2) and the Control simulation from the Northward, Southward, and Neutral Group ensembles. Changes in ERA5 (orange '00**'), JRA-55 (green '**00**'), NCEP (magenta '**00**'), and ERA-20C (blue '00') reanalyses represent differences between 1995-2024 and 1940-1969, 1994-2023 and 1958-1987, 1994-2023 and 1948-1977, and 1981-2010 and 1940-1969, respectively.

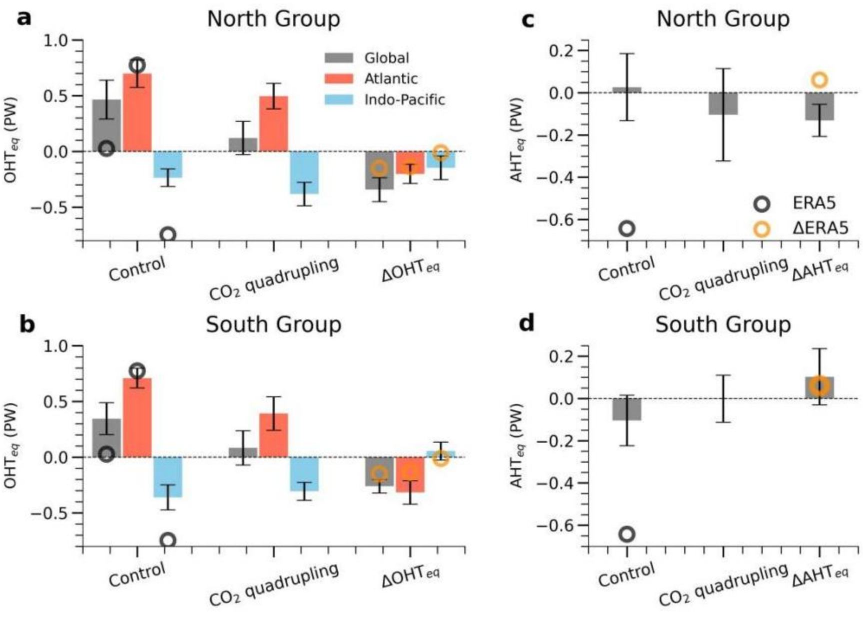

Extended Data Fig. 4 | Northward OHT_{eq} and AHT_{eq}. a, Northward OHT_{eq} for the global (gray), Atlantic (orange), and Indo-Pacific (blue) basins in the Control simulation, CO_2 quadrupling, and their changes (CO_2 quadrupling minus Control), for the *Northward Group*. **b,** Same as **a,** but for *Southward Group*. **c, d,** Same as **a, b,** but for global AHT_{eq}. Error bars indicate one standard deviation for each model group. The black circle represents the observed mean: OHT_{eq} from ORAS5 (1958–2024) or AHT_{eq} from ERA5 (1940–2024). The orange circle shows observed changes, defined as the difference between the last and first

30 years of each respective period. Changes in global northward OHT_{eq} are the -0.34 ± 0.09 PW for the *Northward Group* and –0.25 ± 0.06 PW for the *Southward Group*. Global northward OHT_{eq} in the ORAS5 is 0.03 PW over 1958-2024, 0.11 PW over 1958-1987, and -0.04 PW over 1994-2024. Global northward AHT_{eq} in the ERA5 is –0.64 PW over 1940-2024, -0.67 PW over 1940-1969, and -0.61 PW over 1994-2024. Changes in global AHT_{eq} are -0.13±0.08 PW for the *Northward Group* and +0.10±0.13 PW for the *Southward Group.*

5. Lines 132-134: Does that mean we should see more pronounced ITCZ changes in the Atlantic than the Pacific ITCZ? If the OHT is a dominant driver of ITCZ. Please explain how your results support your description here.

Response: Thank you for this thoughtful comment. To address this question, we explicitly examined basin-scale ITCZ migrations over the Indian, Pacific, and Atlantic Oceans using the same streamfunction-based metric (Extended Data Fig. 12). The results show that, in the Southward Group, the ITCZ migrates southward in both the Atlantic and Pacific basins with a northward movement in the Indian Ocean basin. Moreover, the amplitude of ITCZ movements is largest in the Pacific basin, second in the Atlantic basin. On the other hand, the ITCZ migrates northward in all basins in the Northward Group with the largest movement in the Indian Ocean. Therefore, compounding with the greenhouse gas induced warming, the weakening of AMOC does not lead to a more southward ITCZ movement in the Atlantic basin in comparison with the ITCZ movement in the Pacific basin. This is different from the AMOC weakening alone experiments which show a larger southward ITCZ movement in the Atlantic basin than in the Pacific basin.

Consistent with this pattern, although the global OHT_{eq} in the Southward Group decreases by 0.25 ± 0.06 PW (Extended Data Fig. 4b), the OHT_{eq} reduction is much larger in the Atlantic basin (0.32 ± 0.11 PW) corresponding to a zonal-mean southward displacement of the ITCZ. A regional decomposition further reveals that this reduction in global OHT_{eq} is primarily driven by a pronounced weakening of northward OHT_{eq} in the Atlantic, while changes in the Indo-Pacific basin are comparatively small and partially offset the Atlantic contribution.

These results demonstrate that, when OHT acts as the dominant driver of ITCZ migration, changes in OHT_{eq} provide a physically consistent explanation for the zonal mean ITCZ the Southward Group. In particular, the Atlantic OHT_{eq} reduction plays a leading role in shaping the Atlantic ITCZ shift, whereas OHT_{eq} changes in the Indo-Pacific do not exert a dominant driver of the ITCZ over the Indo-Pacific. This description has been added in the revised manuscript (Lines 482-486).

"For the Southward Group, AMOC weakening directly reduces the northward Atlantic OHT_{eq}, thereby exerting a first-order control on the ITCZ position over the Atlantic basin and the adjacent eastern Pacific. In this regime, the AHT_{eq} associated with CO_2 forcing plays a damping role. Consequently, the combined response of OHT_{eq} and AHT_{eq} favors a southward displacement of the regional ITCZ, and OHT_{eq} is a dominant driver of ITCZ at the global scale and within the Atlantic and Pacific basins."

6. The AMOC change, as shown in Fig. 3 in the South Group, is in line with the No Group. This

raises the question of whether AMOC could be a robust driver of ITCZ.

Response: Thank you for this insightful comment. *We assume that here the reviewer means "the ensemble-mean AMOC changes in the Northward Group are similar to the No Group" since the AMOC change is much larger in the Southward Group*. We agree that the similar magnitude of AMOC weakening between the Northward and Neutral Groups (the phases in the revised text) raises an important question regarding the robustness of AMOC as a driver of ITCZ migration.

Our results suggest that AMOC weakening alone is not sufficient to determine the direction or magnitude of ITCZ migration. Instead, the ITCZ response depends on how OHT_{eq} changes interact with atmospheric circulation responses, and the background mean ITCZ position.

Specifically, in the Southward Group, where the mean ITCZ position is relatively northern, the reduction in northward OHT_{eq} associated with AMOC weakening dominates over the atmospheric contribution, leading to a southward ITCZ migration (Fig. 3 and Extended Data Fig. 4b). In contrast, in the Northward Group, which has a more southern mean ITCZ position, the atmospheric circulation response — including enhanced interhemispheric TS and HC intensity contrasts and strengthened southward AHT_{eq} — overwhelms the opposing effect of the weakened OHT_{eq}, resulting in a net northward ITCZ shift (Fig. 3 and Extended Data Fig. 4a).

For the Neutral Group, both the atmospheric and oceanic circulation responses are intermediate between the Northward and Southward Groups, and their impacts on ITCZ migration largely compensate each other. As a result, the net ITCZ displacement remains small despite a comparable AMOC weakening.

These results indicate that while AMOC weakening plays an important role in modulating ITCZ migration, its impact is strongly conditioned by the atmospheric response and the background ITCZ mean state, rather than acting as a universally robust driver on its own.

7. Lines 189-191 about the tipping point, discuss thereafter: what is your definition of tipping point? Is it an abrupt change, or should it reflect transitions of two stable states? If it is the latter one, are your simulations sufficient to demonstrate stable states, or is what is shown in the present study just transient behavior?

Response: Thank you for raising this important conceptual issue. We agree that the term "tipping point" requires a clear definition and careful interpretation. In the present study, the tipping point refers to a nonlinear change from one stable state to another stable state under gradually increasing forcing. We acknowledge that demonstrating a stable state would require substantially longer integrations. The simulations presented here are therefore not sufficient to conclusively establish the existence of stable ITCZ equilibria, and the possibility that part of the response reflects transient behavior cannot be totally excluded. However, for CESM2 simulations, we do have the ITCZ location stabilized for more than 150 years and could consider this is a quasi-equilibrium state (Fig. 4c).

To avoid ambiguity, we have revised the manuscript to clarify the definition and usage of tipping point, explicitly acknowledging the limitations of the current simulations and emphasizing that the results demonstrate the possible tipping point (Lines 276–278, 356-358, 364-369, 370-378).

"Since the Southward Group, including CESM2, better represents ITCZ migration observed in reanalyses, we use CESM2 as a representative model to explore the complex interplay between a warmer climate and AMOC weakening on ITCZ migration and the potential ITCZ tipping point."

"Following ref 21, "tipping point" refers to the ITCZ crossing a critical threshold from one stable state to another stable state under gradually increasing forcing and the present state doesn't return to the initial state even if the drivers of the change are abated."

"Based on this, the results shown in Figure 4c suggest the existence of three possible quasi-stable ITCZ positions: around 6°N under preindustrial conditions with an active AMOC, near the equator when the AMOC collapses under strong greenhouse gas forcing, and around 6°S when the AMOC collapses due to freshwater forcing alone."

"There are a few unanswered questions regarding the potential ITCZ tipping behavior from the current CESM2 sensitivity experiments. First, whether the above mentioned ITCZ quasi-equilibrium states are true equilibrium states is still not definite, and we cannot totally rule out the possibility that what is shown here could still be a transient behavior of the ITCZ. Second, since the changes of AMOC play a crucial role in determining the ITCZ response to external forcing changes in these experiments, if the ITCZ would return to its preindustrial location after the reversal of the external forcings will depend on whether or not AMOC will recover, and if AMOC recovers, whether this AMOC recovery is linear or nonlinear will determine the linearity of the ITCZ changes in response to the external forcing changes."

8. Lines 284 -285 and lines 286-295: see my review point 3. I do not agree with the statement to use observed southward ITCZ movement as an AMOC change proxy. At least, I don't think the authors show any evidence of that in the current analysis. And in lines 293-295, the Fig. 5 shows a decline in AMOC in 2010. Where do you see AMOC strengthening? The discussions in this paragraph contradict each other. I don't see how you can link AMOC weakening or strengthening to the Arctic sea ice loss.

Response: Thank you for this careful and constructive comment. We agree with the reviewer that the observed southward ITCZ migration should not be interpreted as a direct proxy for AMOC changes, and that such a conclusion was not sufficiently supported in the original manuscript. Following this comment and Comment 3, we have revised the text to modify the statements. Instead, we now frame the relationship more cautiously, emphasizing consistency of the ITCZ migration with AMOC weakening (see revised Lines 25-27, 396-398, and the response to Comment 3).

Regarding Lines 293-295 in the previous version, the original intent was to describe AMOC

strengthening in the early 2000s. To avoid confusion, we have revised this paragraph to clarify the period (revised Lines 430-431).

Finally, we agree that the linkage between AMOC variability and Arctic sea ice loss requires careful qualification. The long-term decline in Arctic sea ice is primarily driven by polar-amplified warming associated with increasing CO_2, while AMOC-related heat transport may modulate the rate of sea ice loss on decadal timescales, rather than acting as a dominant or sole driver (Lines 443-447).

"These findings imply that the observed southward ITCZ migration over recent decades is consistent with a weakened AMOC, within the context of coupled atmosphere-ocean interactions."

"Given that AMOC weakening is one of several contributing mechanisms to the southward ITCZ movement, does the observed ITCZ shift southward imply an overall AMOC decline over the last several decades?"

"Thus, the above changes in NAWH index suggest an overall weakening of AMOC in the last 80 years, with a brief strengthening in the early 2000s."

"While the overall decrease in sea ice extent is commonly attributed to polar-amplified warming in association to the rising atmospheric greenhouse gas concentration, this amplification has been enhanced over the last 60+ years based on reanalysis products which cannot explain the slowdown of the Arctic sea ice loss in recent years. The slowdown of the AMOC can be one of the reasons leading to this slowdown."

References：

1. Fu, Y., Karstensen, J. & Brandt, P. Atlantic Meridional Overturning Circulation at 14.5°N in 1989 and 2013 and 24.5°N in 1992 and 2015: Volume, heat, and freshwater transports. *Ocean Sci.* **14**, 589-616 (2018).
2. Rahmstorf, S. et al. Exceptional twentieth-century slowdown in Atlantic Ocean overturning circulation. *Nat. Clim. Chang* **5**, 475 – 480 (2015).
3. Thornalley, D.J.R. et al. Anomalously weak Labrador Sea convection and Atlantic overturning during the past 150 years. *Nature* **556**, 227-230 (2018).
4. Terhaar, J., Vogt, L. & Foukal, N.P. Atlantic overturning inferred from air-sea heat fluxes indicates no decline since the 1960s. *Nat. Commun.* **16**, 222 (2025).
5. Bryden, H., Longworth, H. & Cunningham, S. Slowing of the Atlantic meridional overturning circulation at 25° N. *Nature* **438**, 655 – 657 (2005).

Reviewer #2

We sincerely thank the reviewer for the thoughtful and constructive comments, which have helped to clarify key methodological and interpretative aspects of the manuscript. In response, we have undertaken substantial revisions to strengthen the robustness of the observational constraints, clarify the level of confidence in attributing ITCZ migration to AMOC variability, and explicitly address limitations associated with the zonal-mean ITCZ framework.

Specifically, we (i) extended the observational analysis beyond ERA5 by incorporating multiple independent reanalysis datasets (JRA-55, NCEP, and ERA-20C) to quantify structural uncertainty and demonstrate the robustness of the diagnosed ITCZ shifts; (ii) revised the interpretation of multidecadal ITCZ–AMOC linkages to explicitly acknowledge uncertainties in AMOC variability and to frame the relationship as plausible but not definitive; and (iii) complemented the zonal-mean ITCZ analysis with basin-scale diagnostics over the Indian, Pacific, and Atlantic Oceans, thereby clarifying regional asymmetries. In addition, minor textual clarifications and new references have been incorporated to improve clarity and completeness.

We believe these revisions address the reviewer's concerns and substantially enhance the physical interpretation and credibility of the study. A detailed point-by-point response is provided below.

Reviewer #2 (Remarks to the Author):

Review of the paper: Intertropical Convergence Zone at Risk of Crossing a Tipping Point Driven by Ocean Circulation Changes in a Warming Climate (Guo et al.)

Summary
This manuscript is a strong and timely contribution to the literature on tropical climate dynamics. It advances understanding in three key ways.
(1) It demonstrates that AMOC weakening is the essential mechanism needed to reconcile observed (ERA5) ITCZ behaviour with CMIP6 model responses. Only models with a more northern ITCZ mean position and a strong AMOC decline reproduce the observed southward migration; warming alone cannot displace the ITCZ farther north once it already resides in a northern state.
(2) It introduces a compelling ITCZ tipping-point framework, showing that the ITCZ can occupy multiple quasi-stable equilibrium positions depending on the state and forcing of the AMOC. This highlights that ITCZ shifts are not indefinitely gradual but may become locked into distinct dynamical regimes.
(3) It proposes observed ITCZ migration as a new independent proxy for long-term AMOC variability, a valuable insight given that direct AMOC observations span only about two decades.

Collectively, these contributions clarify model–observation inconsistencies, deepen theoretical understanding, and point to practical early-warning indicators for large-scale ocean–atmosphere reorganisations.

Major Issues

While the manuscript is strong, several important caveats should be more explicitly acknowledged and addressed:

(1) Dependence on ERA5 for small ITCZ shifts. The central linkage between ITCZ migration and AMOC weakening relies heavily on ERA5's ability to accurately resolve subtle latitudinal displacements. Since these variations are small and reanalysis products contain structural uncertainty, the robustness of the observational constraint has not been fully quantified.

Response: Thank you for this insightful comment. We agree that relying solely on ERA5 may limit the robustness of the results. To further assess this, we have incorporated the monthly, 1.25°×1.25° Japanese 55-year Reanalysis (JRA-55) dataset over 1958-2020 (Kobayashi et al., 2015), the monthly 2.5°×2.5° National Centers for Environmental Prediction (NCEP) over 1948-2023 (Whitaker et al. 2008), and ECMWF Twentieth Century Reanalysis (ERA-20C) over 1940-2010 (Poli et al. 2016) to analyze interhemispheric TS contrast and ITCZ movement. The mean ITCZ positions from JRA-55 (5.89°N), NCEP (5.81°N), and ERA-20C (4.78°N), closely match ERA5 (5.60°N) as well as the results from the Southward Group models (5.11°N; Fig. 1). Moreover, the ITCZ southward shift in JRA-55 (1.29°), NCEP (1.73°), and ERA-20C (1.13°) is consistent with both ERA5 (0.61°) and the Southward Group (1.20°; Fig. 3). These comparisons confirm that the conclusions drawn from ERA5 are robust. In the revised manuscript, additional reanalysis data (JRA-55, NCEP, and ERA-20C) and the corresponding results have been added to strengthen the credibility of our findings (Lines 68-77, 116-122, 129-132, 153-154, 733-737).

"Here we examine the relative contributions of atmospheric and oceanic circulation changes to the annual and zonal mean ITCZ position (hereafter we simply use "ITCZ" to represent the annual and zonal mean ITCZ) and explore the processes driving the ITCZ across potential tipping point by analyzing the fifth generation European Centre for Medium-Range Weather Forecasts (ECMWF) reanalysis (ERA5)[42], the Japanese 55-year Reanalysis (JRA-55)[43], the National Centers for Environmental Prediction (NCEP)[44], the ECMWF Twentieth Century Reanalysis (ERA-20C)[45], model experiments with a warmer background base state (represented here by the idealized 1% CO2 experiments (hereafter simply 1%CO2) from the Coupled Model Intercomparison Project Phase 6 (CMIP6)), and a suite of specially designed sensitivity experiments using the Community Earth System Model version 2 (CESM2; Methods)[46] and the EC-Earth3 model[47]."

"All the reanalyses show a clear southward ITCZ migration by 0.61° (ERA5) 1.29° (JRA55), 1.73° (NCEP), and 1.13° (ERA20C) from the early period (6.06°N over 1940-1969 (ERA5), 6.54°N over 1958-1987 (JRA55), 6.83°N over 1948-1977 (NCEP), and 5.39°N over 1940-1969 (ERA20C)) to recent period (5.45°N over 1995-2024, 5.24°N over 1994-2023, 5.10°N over 19934-2023, and 4.25°N over 1981-2010) , respectively, while CMIP6 models project

inconsistent ITCZ migrations at approximately CO_2 quadrupling in the 1%CO_2 experiment (mean over years 131-150; Fig. 1).”

“Moreover, the standard deviation of the ITCZ position is much larger for Northward Group (1.60 $\pm$ 0.49°) than other groups (1.14 $\pm$ 0.38° for Neutral Group and 0.82 $\pm$ 0.23° for Southward Group), and this standard deviation is 0.86° for ERA5, 0.79° for JRA-55, 1.03° for NCEP, and 0.87° for ERA-20C.”

“Most Southward Group models are consistent with reanalysis, where a positive interhemispheric HC contrast aligns with a southward AHT_{eq} (Fig. 2f).”

“For these reanalysis data, the change in TS difference is quantified as the difference between the last and the first 30-year periods, such as ERA5 (1995-2024 and 1940-1969), JRA-55 (1994-2023 and 1958-1987), NCEP (1994-2023 and 1948-1977), and ERA-20C (1981-2010 and 1940-1969). For the CMIP6 models, the change is defined as the difference between the last 20-year mean of the 1%CO2 experiments and the Control simulation.”

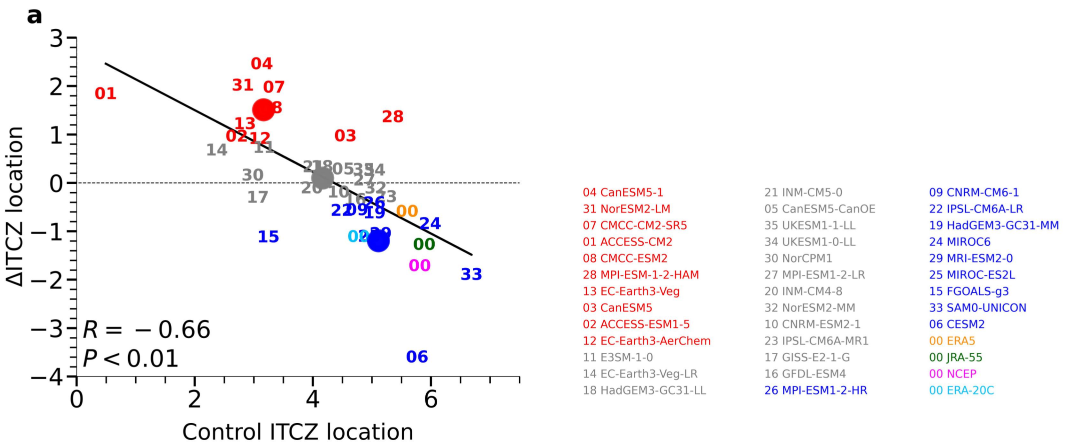

Fig. 1 | Relationships in the ITCZ location and ITCZ migration. a, Relationships across CMIP6 models between climatological mean ITCZ position and ITCZ migration from the preindustrial control (hereafter “Control”) to the late-stage response of the CMIP6 1%CO2 experiment, defined as the mean over years 131-150, when atmospheric CO2 reaches approximately four times its preindustrial level. The historical ITCZ location based on ERA5 (orange ‘**00**’), JRA-55 (green ‘**00**’), NCEP (magenta ‘**00**’), and ERA-20C (blue ‘**00**’) are approximately 5.50°N over 1940-2024, 5.89°N over 1958-2023, 5.81°N over 1948-2023, and 4.78°N over 1940-2010. The ITCZ migration is calculated as the ITCZ difference from the early period (1940-1969, 1958-1987, 1948-1977, and 1940-1969) to the recent period (1995-2024, 1994-2023, 1994-2023, and 1981-2010). The *Northward*, *Southward*, and *Neutral Group* ensembles are shown separately. One standard deviation for each model group is shown.

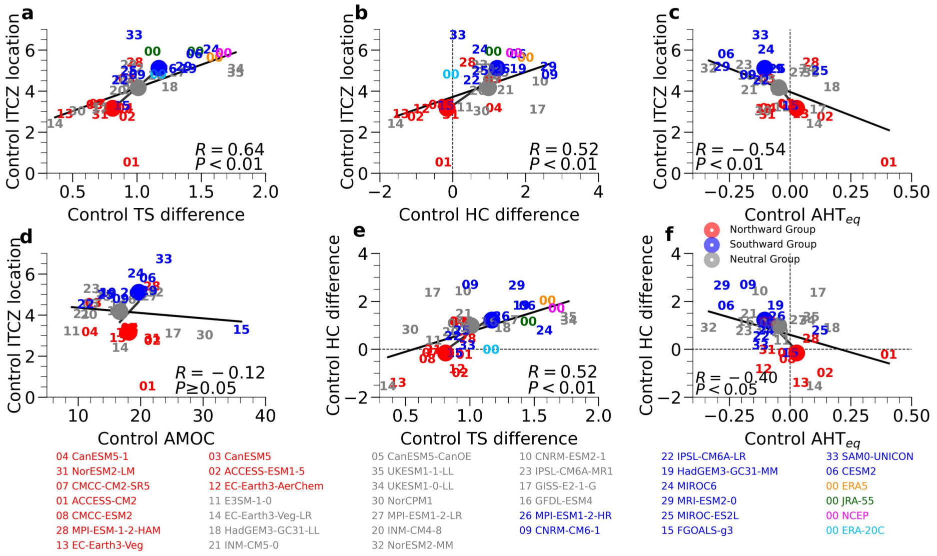

Fig. 2 | Relationships in preindustrial control simulations. a–d, Relationships across CMIP6 models between the mean ITCZ position and: the tropical TS difference between 0°–30°N and 0°–30°S (**a**); hemispheric HC intensity difference (**b**); the northward AHT_{eq} (**c**); and AMOC strength (**d**). **e, f**, Relationships between HC intensity difference and: the TS difference (**e**), and the northward AHT_{eq} (**f**). ERA5 (orange '**00**'), JRA-55 (green '**00**'), NCEP (magenta '**00'**), and ERA-20C (blue '**00'**) reanalysis data represent the average over 1940–2024 1958-2023, 1948-2023, and 1940-2010 respectively. The correlation (*R*) and *P* value of a linear fit (black solid line) are also shown. The *Northward*, *Southward*, and *Neutral Group* ensembles are shown.

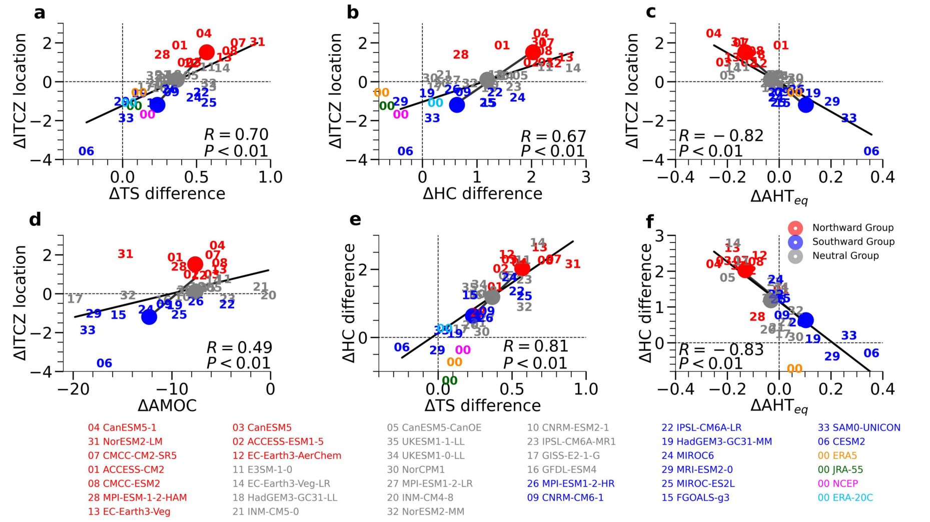

Fig. 3 | Same as **Fig. 2,** but showing changes between CO_2 quadrupling (mean over years 131-150 of 1%CO_2) and the Control simulation from the *Northward*, *Southward*, and *Neutral Group* ensembles. Changes in ERA5 (orange '**00'**), JRA-55 (green '**00'**), NCEP (magenta '**00'**), and ERA-20C (blue '**00'**) reanalyses represent differences between 1995-2024 and 1940-1969, 1994-2023 and 1958-1987, 1994-2023 and 1948-1977, and 1981-2010 and 1940-1969, respectively.

(2) Ambiguity in attributing multidecadal AMOC variability. Although the observed ITCZ behaviour is consistent with AMOC weakening, attribution remains plausible but not definitive. AMOC variations prior to 2004 are poorly constrained, and proxy-based reconstructions are influenced by multiple drivers. The manuscript would benefit from clarifying the level of confidence associated with this inference.

Response: Thank you for this important and thoughtful comment. We agree that attributing multidecadal ITCZ variability to AMOC changes involves substantial uncertainty and should be interpreted with appropriate caution. While the ITCZ behavior in our study is consistent with a weakened AMOC, we emphasize that this relationship should be regarded as plausible rather than definitive. Direct observations of the AMOC are limited to the post-2004 RAPID array, and earlier variability is primarily inferred from proxy records or indirect diagnostics, which remain imperfectly constrained. In the revised manuscript, we adopted another AMOC proxy (Fig. 5) and cited more references to support the present conclusion that the AMOC weakening may be ongoing over the past decades.

Existing reconstructions provide mixed evidence regarding long-term AMOC changes. Several proxy-based studies suggest a weakening of the AMOC since the late nineteenth or early twentieth century (e.g., Rahmstorf et al., 2015; Thornalley et al., 2018), whereas more recent estimates based on air-sea heat fluxes indicate no significant long-term decline since the 1960s (Terhaar et al., 2025). Moreover, AMOC proxies can be influenced by multiple physical processes beyond overturning strength alone, further complicating attribution. In light of these uncertainties, we have revised the text to explicitly acknowledge that AMOC weakening represents one potential contributing mechanism to the observed ITCZ southward shift. We now more clearly state the limitations of available AMOC reconstructions and frame our interpretation within a probabilistic and hypothesis-consistent context (Lines 25-27, 85-86, 392-398, 434-437, 499-505)

"These findings imply that the observed southward ITCZ migration over recent decades is consistent with a weakened AMOC, within the context of coupled atmosphere-ocean interactions."

"Under this framework, the observed southward ITCZ migration in recent decades is consistent with, but does not uniquely imply, an ongoing weakening of the AMOC."

"Because direct observations of the AMOC are limited to the post-2004 period, the long-term variability of AMOC beyond 20 years relies mostly on AMOC proxies, such as the sea surface temperature (SST) contrast between subpolar North Atlantic and the global mean[37], sea surface salinity[55], surface heat flux[56] etc. These proxies are subject to substantial uncertainty and may reflect multiple physical processes beyond changes in overturning strength alone[30,56-58]. Given that AMOC weakening is one of several contributing mechanisms to the southward ITCZ movement, does the observed ITCZ shift southward imply an overall AMOC decline over the last several decades?"

"This long-term overall ITCZ southward movement in the recent 80+ years derived from multiple reanalysis products could be a sign of overall weakening of the AMOC in this period.

Although a more definitive demonstration of this relationship for observations still needs more work, we give the following indirect evidence."

"We further propose that the observed southward ITCZ migration in the reanalysis products could serve as a proxy for long-term AMOC weakening (decadal or longer timescales), supported by the concurrent slowdown in Arctic sea ice decline and intensification of the NAWH. Given the ongoing AMOC weakening and CMIP6 projections[67] since 2005, it is plausible that the ITCZ will continue to shift further southward in the coming decades. If the AMOC would weaken more dramatically, the ITCZ could jump to the equator and greatly alter global precipitation patterns by reorganizing the atmospheric and oceanic circulations, resulting in far reaching impacts on the Earth's climate."

(3) Use of a zonal-mean ITCZ metric. By diagnosing the ITCZ primarily through its zonal-mean latitude, the study may obscure substantial longitudinal asymmetries and regionally important rainfall responses. This limitation is particularly relevant when discussing "tipping points" and quasi-stable states, which may manifest differently across individual ocean basins.

Response: Thank you for this important comment. We agree that diagnosing the ITCZ solely using a zonal-mean metric may obscure longitudinal asymmetries and regionally distinct responses, which is particularly relevant when considering tipping points or quasi-stable states. To address this limitation, we have conducted an additional basin-scale analysis of ITCZ migration across the Indian, Pacific, and Atlantic Oceans using the same streamfunction-based metric, with results shown in Extended Data Fig. 12.

This regional decomposition reveals that, for the Northward Group, although all basins show a northward ITCZ movement, the global ITCZ shift is primarily driven by changes over the Indian Ocean, while Atlantic and Pacific contributions are relatively small. In contrast, for the Southward Group, the global ITCZ migration is dominated by coherent southward shifts over the Pacific and Atlantic Oceans with a northward movement in the Indian Ocean. Although the zonal-mean metric captures the primary global-mean ITCZ response, these basin-scale analyses demonstrate that the ITCZ exhibits substantial regional differences and longitudinal asymmetries. We have therefore added a discussion of the basin-scale ITCZ responses to clarify the limitations of the zonal-mean metric and to place our "tipping points" interpretation in a regional context (Lines 477-498).

"At the regional scale, the contributors to the global ITCZ migration differ markedly between the two groups. For the Southward Group, the global ITCZ shift is dominated by coherent southward displacements over the Pacific and Atlantic Oceans with a northward displacement in the Indian Ocean, whereas for the Northward Group, the global ITCZ migration primarily arises from changes over the Indian Ocean with minor contributions from the Atlantic and Pacific basins (Extended Data Fig. 12). For the Southward Group, AMOC weakening directly reduces the northward Atlantic OHTeq, thereby exerting a first-order control on the ITCZ position over the Atlantic basin and the adjacent eastern Pacific. In this regime, the AHTeq associated with CO2 forcing plays a damping role. Consequently, the

combined response of OHTeq and AHTeq favors a southward displacement of the regional ITCZ, and OHTeq is a dominant driver of ITCZ at the global scale and within the Atlantic and Pacific basins. By contrast, owing to global ocean connectivity, anomalies in Atlantic OHTeq are dynamically linked to the Indo-Pacific circulation, leading to compensatory adjustments in the meridional OHTeq over the Indo-Pacific basin (Extended Data Fig. 4a, b). These oceanic adjustments interact with the interhemispheric TS contrast and induce corresponding changes in AHTeq. The coupled responses of OHTeq and AHTeq together regulate the global zonal-mean ITCZ position and promote a northward migration for the Northward Group. As thus, the zonal-mean ITCZ metric should be interpreted as an integrated indicator of hemispheric-scale energy balance rather than as evidence of uniform behavior across all longitudes. Consequently, the "tipping points" behavior identified in the zonal-mean ITCZ primarily reflects a large-scale response, while regional ITCZ adjustments may exhibit distinct magnitudes, directions, or degrees of stability across individual basins. Recognizing this distinction is essential for interpreting both the physical mechanisms and the broader implications of ITCZ shifts under climate forcing."

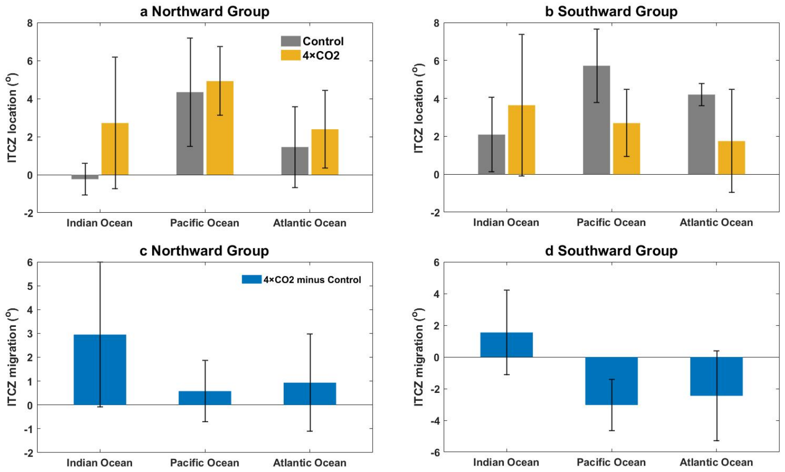

Extended Data Fig. 12 | ITCZ location and migration across ocean basins. a, Ensemble-mean climatological ITCZ position in the preindustrial control (gray) and during years 131-150 of the CMIP6 1%CO2 experiment (yellow), shown separately for the Indian Ocean (20°E-105°E), Pacific Ocean (105°E-85°W) and Atlantic Ocean (85°W-20°E) sections for the Northward Group. Error bars denote one standard deviation across models within each group. **b** Same as **a**, but for the Southward Group. **c-d** Same as **a-b**, but showing ITCZ migration, defined as the change in ITCZ position from the preindustrial climatology to the CO2 quadrupled state.

Minor Comments

(1) The phrase "southward ITCZ migration of –0.61°" is redundant; southward migration already implies a negative sign. Consider rephrasing for clarity.

Response: Thank you for pointing this out. We have revised the sentence to "a southward ITCZ migration of 0.61°".

(2) You may wish to cite the following paper to highlight known challenges in determining

ITCZ position across different diagnostics: Ramotubei, T.S., Landman, W.A., Mateyisi, M.J., Nangombe, S., Beraki, A.F. (2025). Response of the global ITCZ to different ENSO phases and how the ITCZ determined from the maximum precipitation compares with the surface tropical winds convergence. Environmental Development, 55, 101210. This work underscores the sensitivity of ITCZ identification to the chosen metric.

Response: Thank you for this helpful suggestion. We agree that the diagnosed ITCZ position can be sensitive to the choice of metric. We have now cited Ramotubei et al. (2025) and added a brief description acknowledging the challenges in defining the ITCZ across different diagnostics. The added text is (Lines 96-113):

"There are multiply ways to define the ITCZ—precipitation-based methods (e.g., the latitude of maximum precipitation[48], the precipitation centroid[49], the brightness temperature[50]), and the atmospheric circulation based methods (e.g., vertical pressure velocity[51] and the boundary between northern and southern HCs[41]). To assess the sensitivity of the diagnosed ITCZ position to a chosen metric, we compare the atmospheric circulation-based ITCZ location (specifically the ITCZ position is defined as the latitude at which the vertically integrated atmospheric mass streamfunction over 300-700 hPa crosses zero[41]) with the precipitation-based definition (the latitude of maximum precipitation[48]) as an illustrative example. The streamfunction-based ITCZ is generally located farther south than the precipitation-based ITCZ under both preindustrial control and CO_2 quadrupling conditions (Extended Data Fig. 1a-b). However, in response to CO_2 quadrupling in the 1%CO_2 experiment, the streamfunction-based definition exhibits substantial inter-model diversity in its response to CO_2 quadrupling, whereas the precipitation-based definition shows a southward ITCZ movement in all models with different magnitudes. These differences highlight that the choice of ITCZ definition is critical for absolute ITCZ movements, but they might not affect the interpretation of underlying physical processes governing the ITCZ responses to external forcing changes. Because we will use the atmospheric energy constraint theory to explain the physical processes, we therefore adopt an atmospheric circulation-based ITCZ definition in this study[41] as this energy constraint theory provides a close relationship between HCs and AHT_{eq}."

References:

1. Rahmstorf, S. et al. Exceptional twentieth-century slowdown in Atlantic Ocean overturning circulation. *Nat. Clim. Chang* **5**, 475–480 (2015).

2. Thornalley, D.J.R. et al. Anomalously weak Labrador Sea convection and Atlantic overturning during the past 150 years. *Nature* 556, 227-230 (2018).

3. Terhaar, J., Vogt, L. & Foukal, N.P. Atlantic overturning inferred from air-sea heat fluxes indicates no decline since the 1960s. *Nat. Commun.* **16**, 222 (2025).

● **Reviewer #3**

We sincerely thank the reviewer for the thorough, critical, and constructive evaluation of the manuscript. The comments prompted substantial revisions that significantly improved conceptual clarity, physical interpretation, and presentation of the study. In particular, we have carefully reassessed the strength of our conclusions, clarified the definition and limitations of the proposed ITCZ "tipping point," expanded the physical explanation of the atmospheric and oceanic mechanisms governing ITCZ shifts, and improved the transparency of our experimental design and figure presentation.

Specifically, we (i) refined the definition and usage of the "tipping point" concept following the IPCC framework, revised the manuscript title and text to explicitly acknowledge limitations related to reversibility and stability, and avoided overstating the evidence; (ii) strengthened the analysis of ITCZ sensitivity by incorporating additional sensitivity experiments with EC-Earth3, thereby providing a direct contrast between representative models from the Northward and Southward Groups; (iii) clarified the physical origins of atmospheric contributions to ITCZ shifts, including HC intensity, atmospheric heat transport, and TS contrasts, with explicit references to prior work; (iv) improved consistency and clarity in figure presentation, including revising Fig. 1 to explicitly show the relationship between mean ITCZ position and ITCZ migration, harmonizing the presentation of individual-model and group-mean results across figures, and adding a clear roadmap at the end of the Introduction; and (v) clarified methodological choices, including the interpretation of transient versus quasi-equilibrium responses and the role of internal variability versus forced responses in reanalysis-based ITCZ trends.

We believe these revisions directly address the reviewer's concerns regarding overstated conclusions, insufficient physical explanation, and presentation choices, and they substantially enhance the rigor, transparency, and interpretability of the manuscript. Detailed point-by-point responses to each comment are provided below.

Reviewer #3 (Remarks to the Author):

Summary:

The ITCZ, a planetary scale band of precipitation in the deep tropics, controls much of the world's rainfall. It shifts North and South on seasonal and longer timescales and this movement is important to understand in the present and future. The ITCZ is associated with the ascending branch of the Hadley circulation and the atmospheric energy flux equator, leading to tropical control over ITCZ position through the exact position of ascent and extratropical control over ITCZ position through an energy balance perspective. One of the major extratropical influences on ITCZ position is the Atlantic Meridional Overturning Circulation (AMOC), which transports energy to the North in the Atlantic basin, shifting the ITCZ northward.

In the manuscript "Intertropical Convergence Zone at Risk of Crossing a Tipping Point

Driven by Ocean Circulation Changes in a Warming Climate," Guo et al. discuss how the ITCZ will shift in a warming climate, emphasizing the role of ocean circulation, and, in particular, changes in the AMOC. The paper contains several interesting results, including 1. that AMOC weakening controls intermodel differences in ITCZ shifts but not pre-industrial ITCZ position, and 2. that warming alone without a weakening AMOC cannot explain the southward ITCZ shifts under climate change. However, the results are significantly overstated throughout the paper, especially in the discussion and title. Additionally, there are several unclear presentation choices and gaps in physical understanding in the current version which should be fixed if the paper is to be published.

Major comments:

1. Overstated Conclusions: There are several conclusions the authors seem to have which are not convincingly demonstrated. These are problematic to a varying degree, listed below:

(1) Tipping Point: I see little to no evidence of a tipping point in the data displayed. There is certainly not enough evidence for the title of the manuscript. The concept of a tipping point is introduced in the section starting on line 236, but is not defined carefully. Most reasonable definitions of a tipping point refer to an irreversible, sudden, and large change in response to a relatively small change in forcing. The reversibility of the ITCZ shift is not tested, the changes don't seem particularly sudden, and ITCZ changes are not disproportionately large compared to the fractional change in AMOC strength (Fig. 4e). Additionally, there are no two experiments which show the same final ITCZ position after several hundred years, so I don't see how the authors can conclude that there are "three quasi-stable ITCZ positions." To substantiate the claim of a tipping point, the authors need to rigorously define the concept referring to past work and show that their simulations prove the existence of such a change.

Response: Thank you for this insightful comment. We agree that the concept of a "tipping point" needs to be defined carefully. In this study, we use the term to describe a nonlinear transition of the ITCZ between quasi-stable states in response to gradually increasing external forcing, associated with the crossing of a critical threshold. Beyond this threshold, the system exhibits a markedly different mean state and does not readily return to its original configuration when the forcing is reduced. This usage follows the general framework for tipping elements and critical transitions outlined in IPCC (2021).

In our CESM2 sensitivity experiments, the ITCZ position remains largely stable for at least 100 years in each simulation, which we interpret as possible quasi-stable states. However, we acknowledge that, given the limited sensitivity experiments, the final stabilization of these states cannot be fully confirmed, and the reversibility of the shifts has not been tested. To avoid ambiguity, we have revised the manuscript and its title to clarify our use of "tipping point" and explicitly acknowledge the limitations of our simulations. (Lines 276-278, 356-358, 364-378).

"Since the Southward Group, including CESM2, better represents ITCZ migration observed in reanalyses, we use CESM2 as a representative model to explore the complex interplay

between a warmer climate and AMOC weakening on ITCZ migration and the potential ITCZ tipping point.”

“Following ref 21, “tipping point” refers to the ITCZ crossing a critical threshold from one stable state to another stable state under gradually increasing forcing and the present state doesn’t return to the initial state even if the drivers of the change are abated.”

“Based on this, the results shown in Figure 4c suggest the existence of three possible quasi-stable ITCZ positions: around 6°N under preindustrial conditions with an active AMOC, near the equator when the AMOC collapses under strong greenhouse gas forcing, and around 6°S when the AMOC collapses due to freshwater forcing alone. The ITCZ position is thus closely tied to the state of the AMOC and the specific mechanism driving its collapse — whether freshwater or greenhouse gas forcing.

There are a few unanswered questions regarding the potential ITCZ tipping behavior from the current CESM2 sensitivity experiments. First, whether the above mentioned ITCZ quasi-equilibrium states are true equilibrium states is still not definite, and we cannot totally rule out the possibility that what is shown here could still be a transient behavior of the ITCZ. Second, since the changes of AMOC play a crucial role in determining the ITCZ response to external forcing changes in these experiments, if the ITCZ would return to its preindustrial location after the reversal of the external forcings will depend on whether or not AMOC will recover, and if AMOC recovers, whether this AMOC recovery is linear or nonlinear will determine the linearity of the ITCZ changes in response to the external forcing changes.”

(2) North vs. South Group Sensitivity: Lines 305-313 discuss how the mean ITCZ position changes its response to warming. I think these are the most interesting results, but there are two problems. First, most of the paper is in terms of north vs. south group, which refers to changes with warming not mean position (I have a comment on that below). Second, only one model is used for the sensitivity experiments, it is hard to come to these conclusions when only a model from the south group is tested. It would be very helpful to also run experiments with a model from the Northward Group. (This is not as big a problem as the previous comment, but would still improve the paper)

Response: Thank you for this helpful comment. For the first question, we have renamed the “North” and “South” groups as the “Northward” and “Southward” groups throughout the main text and figure captions, including the Extended Data, to more clearly indicate that these labels refer to changes in ITCZ position rather than its mean location (for example, Lines 124-127).

For the second question, we have use EC-Earth3—a model from the Northward Group—to compare with CESM2. This choice is motivated by the fact that three closely related CMIP6 models—EC-Earth3-Veg, EC-Earth3-Veg-LR, and EC-Earth3-Aerchem—exhibit a northward ITCZ migration in response to CO_2 quadrupling. EC-Earth3 was not included in the initial analysis because the data download was unsuccessful at that time. The available experiments from EC-Earth3 are a bit different from those of CESM2 as detailed below:

In addition to the 300-year preindustrial control (Control) simulation, three sensitivity experiments are analyzed: Hosing, fixAMOC, and Abrupt-4xCO_2 using the r3i1p1f1 and r8i1p1f1 ensemble members. In the Hosing experiment, a uniform freshwater forcing of 0.3 Sv is applied to the subpolar North Atlantic plus Arctic region from 50°N to the Bering Strait during 1850–1991. In the Abrupt-4×CO_2 experiment, the atmospheric CO_2 concentration is instantaneously increased to four times its preindustrial level. In the fixAMOC experiment, a positive salinity anomaly equivalent to 0.6 Sv negative freshwater flux is imposed north of 50°N over the Arctic and North Atlantic from 1850 to 2102 under Abrupt-4×CO_2 forcing in order to maintain a nearly constant AMOC strength.

The mean ITCZ position in the EC-Earth3 Control simulation is located at 3.3°N (Extended Data Fig. 10a), which is close to the ensemble-mean ITCZ position of the Northward Group (3.2°N; Fig. 1). Consistent with this classification, EC-Earth3 exhibits a northward ITCZ migration under Abrupt-4×CO_2 forcing, primarily driven by an enhanced interhemispheric HC intensity contrast between the southern and northern branches (Extended Data Fig. 10b), partly offset by the weakening of the AMOC (Extended Data Fig. 10c). When the AMOC strength is constrained in the fixAMOC experiment, the ITCZ migrates farther northward, dominated by the strengthened HC intensity contrast. In contrast, the Hosing experiment produces a southward ITCZ shift, mainly driven by pronounced AMOC weakening, with an additional contribution from the reduced interhemispheric HC contrast. In the revised manuscript, we have added the comparison between EC-Earth3 sensitivity results and CESM2 sensitivity results. This comparison between representative models from the Northward and Southward Groups supports the robustness of our conclusions regarding the sensitivity of ITCZ shifts to mean-state differences (Lines 328-354, 707-718).

"Thus, we classify the models into three groups based on the direction of ITCZ shifts: Northward (red numbers), Southward (blue numbers), and Neutral (gray numbers) Groups where the big solid circles represent the ensemble mean of each group (Fig. 1)."

"For completeness, we also examine a suite of sensitivity experiments —including fixAMOC, Hosing, and Abrupt-4 × CO_2 (Methods) — using EC-Earth3, one of the models in the Northward Group, to isolate the impacts of different forcing mechanisms on ITCZ shifts. EC-Earth3 was not included in the initial analysis owing to temporary data availability issues. In the preindustrial control simulation, EC-Earth3 features a mean ITCZ position at 3.3°N (Extended Data Fig. 10a), consistent with the characteristics of the Northward Group. Under Abrupt-4 × CO_2 forcing, EC-Earth3 exhibits a northward ITCZ migration (to ~4°N, primarily associated with a strengthening of the interhemispheric HC intensity contrast between the southern and northern branches (Extended Data Fig. 10b), while concurrent AMOC weakening partially offsets the northward displacement (Extended Data Fig. 10c). When the AMOC strength is constrained in the fixAMOC experiment, the ITCZ migrates farther northward (to ~6°N), indicating that the enhanced interhemispheric HC contrast exerts a dominant control on the ITCZ position in the absence of AMOC-induced compensation. In contrast, the Hosing experiment produces a southward ITCZ shift, driven mainly by pronounced AMOC weakening, with the associated reduction in interhemispheric HC intensity contrast further reinforcing the southward displacement. Together, these sensitivity

experiments show that generally weakening of the AMOC induces a southward ITCZ movement and the greenhouse gas-induced warming leads to a northward ITCZ movement, a conclusion consistent with those obtained from CESM2 sensitivity experiments. The most noticeable difference between CESM2 and EC-Eaths3 is the relative contribution by these two forcings —AMOC changes dominate the ITCZ movement in CESM2 and greenhouse gas forcing dominates the ITCZ movement in EC-Earth3. As a result, with elevated greenhouse gas and the resulting AMOC weakening, the ITCZ moves northward in EC-Earth3 (also other models in Northward Group), but southward in CESM2 (also models in Southward Group). Although our analysis cannot clearly demonstrate the exact underlying physical mechanism leading to this different ITCZ response, our speculation is that the different mean ITCZ locations in these two groups of models could be the fundamental cause. An ITCZ much closer to equator or further away from the equator may make it more or less sensitive to the atmospheric (or oceanic) circulation changes, resulting in a different ITCZ movement."

"To examine the dependence of ITCZ migration on its mean position and to provide a comparison with the representative model from the Southward Group, EC-Earth3 is included in this study. This choice is motivated by the fact that three closely related CMIP6 models — EC-Earth3-Veg, EC-Earth3-Veg-LR, and EC-Earth3-Aerchem — exhibit a northward ITCZ migration under CO_2 quadrupling. EC-Earth3 was initially not included due to temporary data unavailability. In addition to the 300-year preindustrial control (Control) simulation, three sensitivity experiments are performed: fixAMOC, Hosing, and Abrupt-4 × CO_2 using the r3i1p1f1 and r8i1p1f1 ensemble members. In the fixAMOC experiment, a salinity anomaly of 0.6 Sv is imposed north of 50°N over the Arctic and North Atlantic from 1850 to 2102 under Abrupt-4 × CO_2 forcing to maintain a nearly constant AMOC strength. In the Hosing experiment, a uniform freshwater forcing of 0.3 Sv is applied to the polar region from 50°N to the Bering Strait during 1850-1991. In the Abrupt-4 × CO_2 experiment, atmospheric CO_2 is instantaneously increased to four times its preindustrial level."

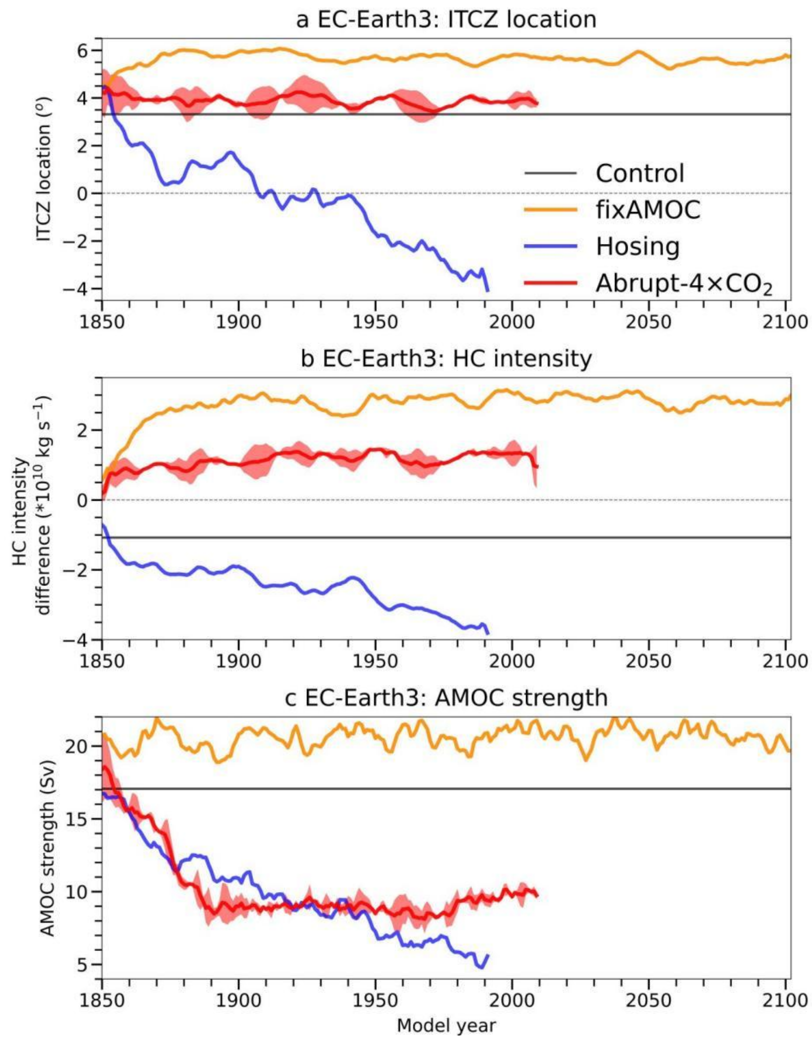

Extended Data Fig. 10 | ITCZ, HC intensity difference, and AMOC intensity. a, The 11-year running mean ITCZ location (**a**), HC intensity difference (**b**), AMOC intensity (**c**) from the EC-Earth3 simulations, including the 300-year preindustrial control mean (gray), *fixAMOC* (orange), *Hosing* (blue), and *Abrupt-4×CO₂* experiments. The Abrupt-4×CO_2 curve shows the ensemble mean of the r3i1p1f1 and r8i1p1f1 members, with shading indicating one standard deviation.

2. Lack of Physical Understanding: There are several aspects of the paper that are interesting but not explained physically.

(1) I believe the main result of the paper is that there is little intermodel correlation between AMOC strength and ITCZ position in the control climate (Fig. 2d), but there is a relationship between change in AMOC strength and change in ITCZ position (Fig. 3d). Why is this?

Response: Thank you for this insightful comment. In the control climate, the intermodel correlation between AMOC strength and ITCZ position is weak when considering all models together (Fig. 2d). This is primarily because the mean-state ITCZ position and AMOC strength are influenced by a range of model-dependent processes and structural biases that are not

uniquely linked to each other, including differences in atmospheric convection, cloud radiative effects, and ocean mixing parameterizations. Consequently, large model-to-model differences in the simulated mean climate obscure a robust relationship between AMOC strength and ITCZ position that emerges only within subsets of models in the control state. Notably, a clearer positive correlation is evident within the Southward Group (excluding one outlying model) with a correlation coefficient of 0.82, which agrees with the conventional knowledge: a stronger AMOC warms the Northern Hemisphere more and leads to a further northward ITCZ mean location (Extended Data Fig. 2b). However, although the relationship between ITCZ location and AMOC strength in the Northward and Neutral Groups is less clear, one still can see an anticorrelation between AMOC strength and ITCZ location in these two group of models—stronger AMOC relates to a further southward ITCZ location which opposites our conventional understanding and further suggests that the biases in representing ITCZ and AMOC could be much larger in these two group of models than in the Southward Group (Extended Data Fig. 2). The fundamental underlying physical processes causing these biases need to be studied further, but is beyond the scope of this study (it is our intention to study this further in the future as this is an important issue to build a more credible model.).

By contrast, the relationship between changes in AMOC strength and changes in ITCZ position under CO2 forcing (Fig. 3d and Extended Data Fig. 2d-f) is much more robust. The forced response emphasizes anomalies relative to each model's own control climate, thereby reducing the influence of mean-state biases and highlighting the dynamically consistent response to external forcing. Under increased CO_2, AMOC weakening emerges as a common response across models, and the associated hemispheric energy imbalance leads to a coherent ITCZ shift, particularly within the Southward Group and across the full model ensemble.

We have clarified this point in the revised manuscript to explicitly distinguish between mean-state intermodel variability and forced responses, and to clarify why AMOC-ITCZ coupling is more evident in the latter case (Lines 158-165, 177-187).

"Additionally, the ITCZ mean position shows no clear correlation with AMOC mean strength (Fig. 2d). But if only the Southward Group is considered, a stronger AMOC is related to a more northward ITCZ (Extended Data Fig. 2b), consistent with the notion that a stronger AMOC transports more heat from Southern to Northern Hemisphere which increases the interhemispheric temperature contrast. On the other hand, in the Northward Group, a stronger AMOC is related to a more southward ITCZ location although this relationship is not statistically significant (Extended Data Fig. 2a). Therefore, this analysis further suggests that the southward group can represent the ITCZ and its associated processes better."

"This means that a larger weakening of the AMOC is related to a more southward ITCZ movement. However, Extended Data Fig. 2d and 2e show that this relationship is true only for the Southward Group with an opposite relationship for the Northward Group—larger AMOC weakening is associated to a more northward ITCZ movement which suggests that changes in AMOC play a very minor role in determining the ITCZ movement in response to the

greenhouse gas forcing, and the atmospheric processes play a more dominant role. Therefore, this result demonstrates that the relationship between AMOC and ITCZ location does not depend on the background mean states. For models with a stronger AMOC-ITCZ relationship in their preindustrial mean state, AMOC also plays an important role in determining the ITCZ's transient response. For models with weak or no AMOC-ITCZ relationships in their preindustrial mean state, AMOC plays only a minor role in ITCZ's transient behavior."

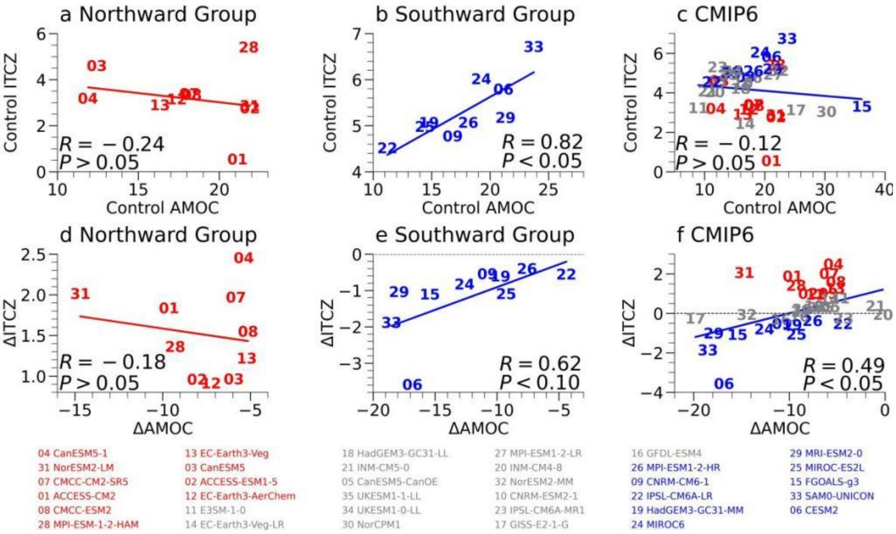

Extended Data Fig. 2 | Relationships between the AMOC and the ITCZ across CMIP6 models. a-c, Intermodel relationships between the climatological mean AMOC strength and ITCZ position for the Northward Group (**a**), Southward Group (**b**), and all CMIP6 models (**c**). **d-f**, Same as **a-c**, but showing the relationship between AMOC weakening and ITCZ migration between CO_2 quadrupling (mean over years 131-150 of the 1%CO_2 experiment) and the preindustrial control simulation. For each panel, the Pearson correlation coefficient (***R***) and the ***P*** value from a linear regression (black solid line) are shown. The FGOALS-g3 model (label 15) is excluded as an outlier.

(2) A lot of the paper is about a contrast between the ocean mechanism for ITCZ shifts (i.e., AMOC changes) and the atmospheric mechanisms (Hadley cell changes, AHT changes, and surface temperature changes). This is a very interesting concept, but the cause of the atmospheric mechanisms is not adequately discussed. Even just referring previous work would be an improvement, the reader is currently given no information about what controls the Hadley cell difference or surface temperature differences.

Response: Thank you for this insightful comment. We agree that the physical origins of the atmospheric mechanisms contributing to ITCZ shifts were not sufficiently described in the original manuscript. To address this, we have expanded the Introduction to explicitly describe the processes that control Hadley circulation intensity or interhemispheric surface temperature differences, with references to previous work (Lines 38-54):

"AHT_{eq} is mainly controlled by the interhemispheric asymmetry of the HC and the inter-tropical surface temperature (TS) contrast in association to the extratropical forcing

and feedback[9]. The HC contrast is strongly governed by the interhemispheric sea surface temperature (SST) difference, and any extratropical forcing or asymmetric climate background that alters this SST contrast tends to shift the ITCZ toward the warmer hemisphere via the adjustment of the HC intensity contrast and the AHT_{eq} *anomaly to balance the global energy budget[7,10-11]. In contrast,* $Qnet_{eq}$ *is influenced by tropical forcing, such as El Niño[1,10,12]. Moreover,* AHT_{eq} *has been identified as the predominant driver in displacing the energy flux equator and ITCZ under a quasi-equilibrium condition[4,6]. The* AHT_{eq} *is aligned with the direction of flow in the upper branch of the HC at the equator and is primarily controlled by the intensity contrast between its Southern and Northern Hemisphere branches. Therefore, the annual mean Southern Hemisphere HC is stronger and extends across the equator into the Northern Hemisphere which leads to a southward flow and AHTeq, and the ITCZ is positioned in the Northern Hemisphere. Changes in HC intensity occur predominantly in the Northern Hemisphere rather than the Southern Hemisphere in a warmer base state[13]. Several mechanisms have been proposed to explain variations in Northern Hemisphere HC strength, including changes in moisture and thermodynamic balances (Held and Soden 2006), the direct radiative effects of* CO_2 *on temperature[15-16], and modifications of meridional temperature gradients[12,17-18].*"

3. Unclear Presentation Choices: There are a few aspects of the presentation for which I don't understand the choices. These should be changed or explained.

(1) Why are all the models shown in Fig. 2 but only group averages in Fig. 3? The relevant data is somewhat shown in extended Fig. 1 and 2, but why wouldn't the two figures in the main text parallel each other?

Response: Thank you for this thoughtful comment. We agree that consistency between Figs. 2 and 3 is important. In the revised manuscript, we have ensured that individual-model results and group-mean behavior are presented in a parallel and complementary manner.

Specifically, Fig. 2 shows the relationships across all individual models in the piControl simulations, overlaid with group-mean values, to illustrate the full inter-model spread and the robustness of the relationships in the control climate. The corresponding results under CO_2 quadrupling are shown using the same format in Extended Data Fig. 3. Figure 3 focuses on the CO_2-induced changes, defined as the difference between the CO_2-quadrupling and piControl simulations. For clarity, this figure emphasizes the group-mean responses of the Northward, Southward, and Neutral Groups, which facilitates a direct comparison of the contrasting group behaviors while avoiding visual overcrowding. Together, Figs. 2 and 3 and Extended Data Fig. 3 now provide a consistent and parallel presentation: individual-model relationships and group means are shown for both the control mean and CO_2-quadrupling climate conditions.

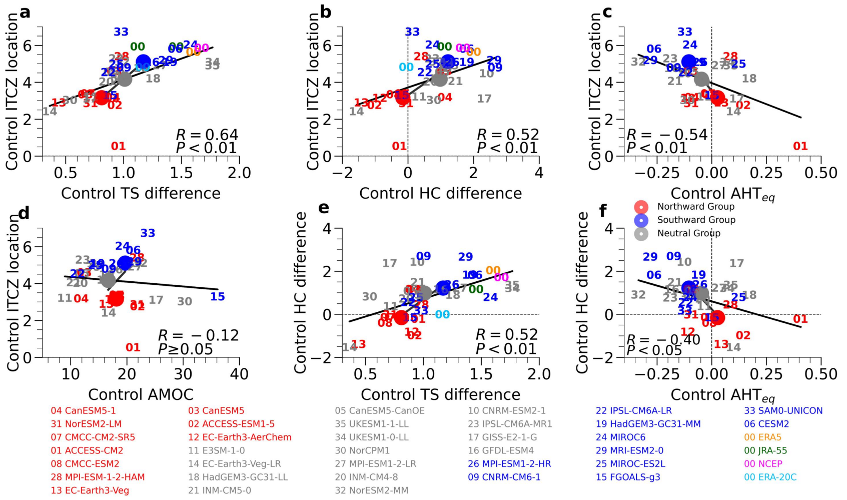

Fig. 2 | Relationships in preindustrial control simulations. a-d, Relationships across CMIP6 models between the mean ITCZ position and: the tropical TS difference between 0°-30°N and 0°-30°S (**a**); hemispheric HC intensity difference (**b**); the northward AHT_{eq} (**c**); and AMOC strength (**d**). **e, f**, Relationships between HC intensity difference and: the TS difference (**e**), and the northward AHT_{eq} (**f**). ERA5 (orange ‘**00**’), JRA-55 (green ‘**00**’), NCEP (magenta ‘**00**’), and ERA-20C (blue ‘**00**’) reanalysis data represent the average over 1940–2024 1958-2023, 1948-2023, and 1940-2010 respectively. The correlation (*R*) and *P* value of a linear fit (black solid line) are also shown. The *Northward*, *Southward*, and *Neutral Group* ensembles are shown.

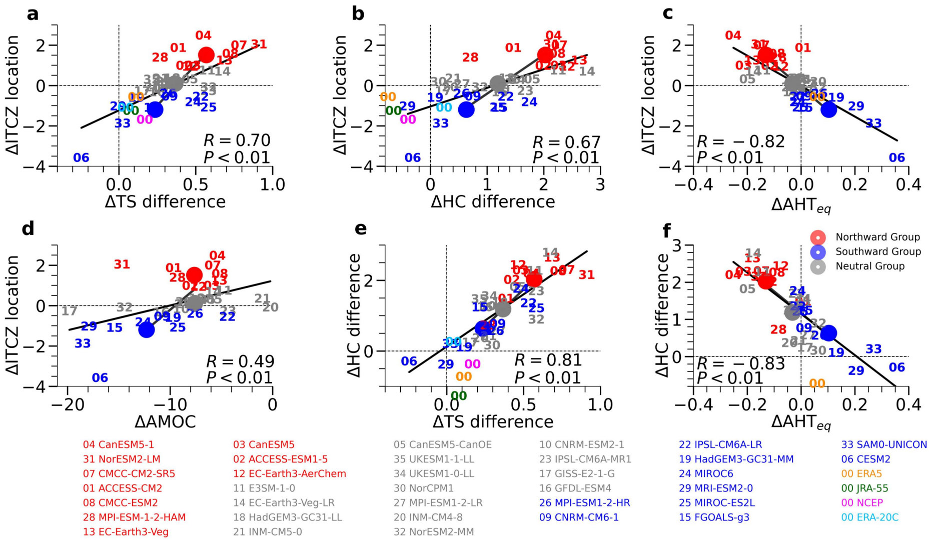

Fig. 3 | Same as **Fig. 2,** but showing changes between CO_2 quadrupling (mean over years 131-150 of 1%CO_2) and the Control simulation from the *Northward*, *Southward*, and *Neutral Group* ensembles. Change in ERA5 (orange ‘**00**’), JRA-55 (green ‘**00**’), NCEP (magenta ‘**00**’), and ERA-20C (blue ‘**00**’) reanalyses represents the differences between 1995-2024 and 1940-1969, between 1994-2023 and 1958-1987, between 1994-2023 and 1948-1977, between 1981-2010 and 1940-1969, respectively.

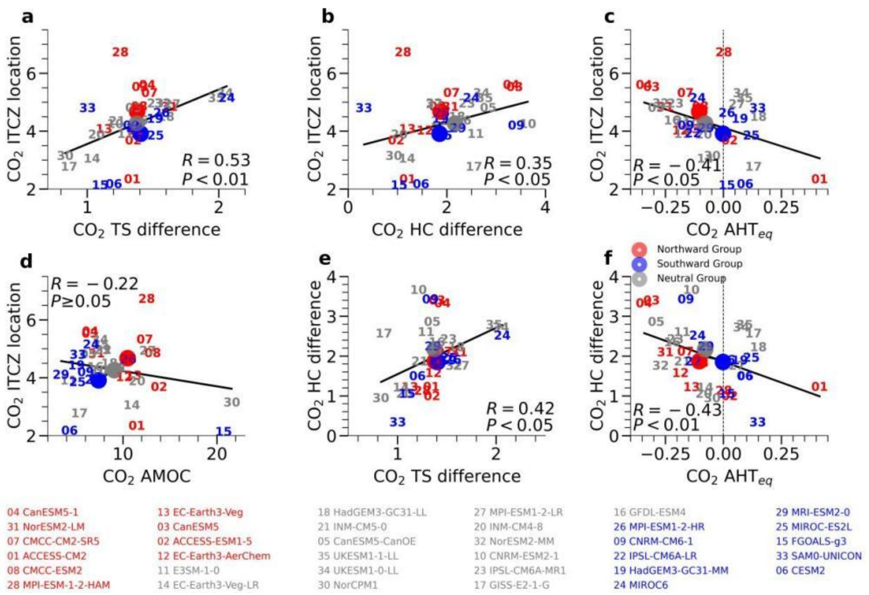

Extended Data Fig. 3 | Relationships under CO_2 quadrupling conditions (mean over years 131-150). a-d, Relationships across CMIP6 models between ITCZ location and: the tropical TS difference between 0°–30°N and 0°–30°S (**a**), hemispheric HC intensity difference (**b**), the northward AHT_{eq} (**c**), and AMOC strength (**d**). **e, f,** Relationships between HC intensity difference and: TS difference (**e**) and northward AHT_{eq} (**f**). The correlation coefficient (*R*) and *P* value for a linear fit (black solid line) are shown. The *Northward*, *Southward*, and *Neutral Group* ensembles are shown.

(2) Fig. 1 as a scatterplot would be nice, is there a correlation between ITCZ position and the change in ITCZ position?

Response: Thank you for this helpful suggestion. Following this comment, we have revised Fig. 1 to explicitly present the relationship between the climatological mean ITCZ position and the CO_2-induced ITCZ migration using a scatterplot.

As shown in the updated Fig. 1, there is a statistically significant negative correlation across CMIP6 models (R=−0.66, P<0.01) between the mean ITCZ position in the control climate and the subsequent change in ITCZ position under CO_2 forcing. This indicates that models with a more northern climatological ITCZ tend to exhibit a southward ITCZ shift, whereas models with a more southern mean ITCZ position tend to shift northward. The group-mean values for the Northward, Southward, and Neutral Groups further highlight this systematic dependence of the ITCZ response on its mean-state position. In addition, all reanalysis datasets (ERA5, JRA-55, NCEP, and ERA-20C) show a relatively northern mean ITCZ position together with a southward shift, lending further support to the Southward Group and the main conclusion of the study.

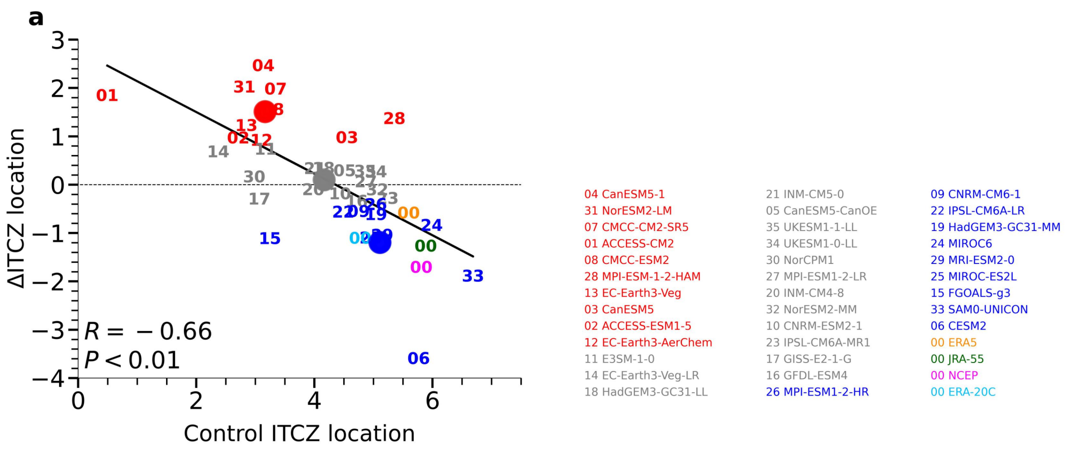

Fig. 1 | Relationships in the ITCZ location and ITCZ migration. a, Relationships across CMIP6 models between climatological mean ITCZ position and ITCZ migration from the preindustrial control (hereafter "Control") to the late-stage response of the CMIP6 1%CO_2 experiment, defined as the mean over years 131-150, when atmospheric CO_2 reaches approximately four times its preindustrial level. The historical ITCZ location based on ERA5 (orange '**00**'), JRA-55 (green '**00**'), NCEP (magenta '**00**'), and ERA-20C (blue '**00**') are approximately 5.50^{o}N over 1940-2024, 5.89^{o}N over 1958-2023, 5.81^{o}N over 1948-2023, and 4.78^{o}N over 1940-2010. The ITCZ migration is calculated as the ITCZ difference from the early period (1940-1969, 1958-1987, 1948-1977, and 1940-1969) to the recent period (1995-2024, 1994-2023, 1994-2023, and 1981-2010). The *Northward*, *Southward*, and *Neutral Group* ensembles are shown separately. One standard deviation for each model group is shown.

(3) An outline of the paper at the end of the introduction would be good to include. On first reading, the progression of results was not clear to me so knowing what is coming would help the reader.

Response: Thank you for this helpful suggestion. We agree that providing a clearer roadmap would improve the readability of the manuscript. Accordingly, we have added a brief outline at the end of the Introduction to summarize the organization of the paper and clarify the progression of the results (Lines 89-94):

"*The remainder of the paper is organized as follows. We first present the observed and simulated ITCZ responses to increasing CO2 forcing. We then examine the underlying dynamical mechanisms driving the ITCZ shifts, with a focus on the relative roles of atmospheric processes and AMOC changes and assess the conditions under which a possible tipping point may emerge. Finally, we place these results in the context of associated AMOC variations and discuss their broader implications.*"

4. Methodological Questions:

(1) Transient vs. Long-term simulated responses/different warming scenarios: The paper uses a mix of different warming scenarios. The introduction mentions 1% increase, then Fig. 1-3 are about years 131-150 after a quadrupling of CO_2, and Fig. 4 is about the 1%

experiments. It is fine to use a combination, but it should be made more explicit what is the purpose of each experiment and if the authors are considering each result to represent a steady-state warmed climate or a transient state.

Response: Thank you for raising this important point. We apologize for the confusion caused by our earlier description of the experiments. In this study, we use a single warming scenario, namely the CMIP6 1%CO_2 (1pctCO_2) experiment.

Figs. 1, 3 show the climate response to the last 20-year of the 1%CO_2 experiment, calculated as the difference between the 131-150-year mean—when atmospheric CO_2 reaches approximately four times its preindustrial concentration—and the piControl simulation. We use this 20-year mean to approximate the quasi-equilibrium response under ~4×CO_2 forcing. In contrast, Fig. 4 presents the full 150-year time series from the 1%CO_2 experiment, highlighting the transient evolution of the ITCZ and associated circulation changes during the gradual CO_2 increase.

We have revised the Introduction, results, and figure captions to explicitly clarify that all results are derived from the 1%CO_2 experiment, and to clearly distinguish between the quasi-equilibrium response and the transient evolution (Lines 77-80, 120-122, 136-139, 167-171). We believe these revisions resolve the confusion regarding the purpose and interpretation of each figure.

"Throughout this study, we use the CMIP6 1%CO2 experiment to approximate the climate response under a CO2 quadrupling condition by using the mean state over years 131-150 of the 1%CO2 simulation when atmospheric CO2 reaches approximately four times of its preindustrial level."

"... while CMIP6 models project inconsistent ITCZ migrations at approximately CO2 quadrupling in the 1%CO2 experiment (mean over years 131-150; Fig. 1)."

*"**a**, Relationships across CMIP6 models between climatological mean ITCZ position and ITCZ migration from the preindustrial control (hereafter "Control") to the late-stage response of the CMIP6 1%CO2 experiment, defined as the mean over years 131-150, when atmospheric CO2 reaches approximately four times its preindustrial level."*

"However, the correlations between ITCZ movement and changes in interhemispheric TS contrast, HC intensity contrast and AHT_{eq} in response to CO_2 quadrupling in the 1%CO_2 experiment become substantially stronger ($|R| \geq 0.67$; Fig. 3a-c), as do the linkages between changes in HC intensity and TS contrasts or AHT_{eq} ($|R| \geq 0.81$; Fig. 3e-f)."

(2) Forced response vs. internal variability in ERA5: The paper talks at length about the observed southward shift in the ITCZ (starting in the introduction and throughout the paper). However, the top of Fig. 5 tells a more complicated and interesting story: that the ITCZ shifted south from 1940-1980 but then North from 1990-2020. How much of this is a forced response vs. internal variability? Reality is only one realization, so how can we tell? The proposed associations with other quantities in Fig. 5 seem to be based on correlations

between three data points, which is not enough to convincingly show that one quantity is correlated with another, let alone establish a causal link as the authors frequently imply. Similarly, the sentence "If this assessment of AMOC variation is true, the observed southward ITCZ movement can be considered as another AMOC change proxy" seems very strange to me, as the figure just above shows that AMOC and ITCZ position are not always correlated (e.g., between the second red lines and third red lines they change in opposite directions).

Response: Thank you for this insightful comment. We acknowledge that the multidecadal variations of the ITCZ in ERA5 — such as the pronounced southward shift from the 1940s-1960s to the 1970s-1990s, followed by a modest northward shift from the 1990s-2020s — are largely dominated by internal variability rather than external forcing. During this period, atmospheric CO_2 concentrations increased only gradually in the mid-20th century, accelerated slightly before the 21st century, and rose sharply over the past decade (IPCC 2021). While internal variability dominates the observed ITCZ fluctuations, the influence of external forcing on ITCZ migration becomes increasingly important under stronger CO_2 increases. In this study, we use the relatively small ITCZ movement under past moderate CO_2 rise to provide context for potential responses under larger future CO_2 forcing. These points have been clarified in the revised text (Lines 401-407).

To assess robustness, we also examined ITCZ positions using three additional reanalysis datasets: JRA-55, NCEP, and ERA-20C. The mean ITCZ positions from JRA-55 (5.89°N), NCEP (5.81°N), and ERA-20C (4.78°N), closely match ERA5 (5.60°N) as well as the results from the Southward Group models (5.11°N; Fig. 1). Moreover, the ITCZ southward shift in JRA-55 (1.29°), NCEP (1.73°), and ERA-20C (1.13°) is consistent with both ERA5 (0.61°) and the Southward Group (1.20°; Fig. 3). These comparisons confirm the robustness of the ERA5 and the Southward Group models. Furthermore, a robust relationship between AMOC strength and ITCZ position in the control state ($R=0.82$, $P<0.05$) and their changes under CO_2 forcing ($R=0.62$, $P<0.10$) for the Southward Group (FGOALS-g3 model is excluded as an outlier). Taken together, these results indicate a close statistical and dynamical association between ITCZ migration and AMOC weakening. Nonetheless, we agree that the observed southward ITCZ migration should not be interpreted as a direct proxy for AMOC changes. In the revised manuscript, we adopted another AMOC proxy (Fig. 5) and cited more references to support the present conclusion that the AMOC weakening may be ongoing over the past decades. Furthermore, we highlight that AMOC proxies can be influenced by multiple physical processes beyond overturning strength alone, further complicating attribution. In light of these uncertainties, we have revised the text to explicitly acknowledge that AMOC weakening represents one potential contributing mechanism to the observed ITCZ southward shift (Lines 25-27, 85-86, 392-398, 431-437, 499-505).

"This low-frequency ITCZ variation can be induced by decadal timescale internal variability alone or a combination of internal variability and external forcing. During this period, the atmospheric CO2 concentration increases by about 25% (from ~300 to 422 ppmv) which is much less than in the 1%CO2 experiments, suggesting the interdecadal ITCZ variations are primarily modulated by internal variability. Nevertheless, the magnitude and structure of the

ITCZ response under past moderate CO2 rise provide useful context for assessing potential ITCZ responses under stronger future CO2 forcing."

"These findings imply that the observed southward ITCZ migration over recent decades is consistent with a weakened AMOC, within the context of coupled atmosphere-ocean interactions."

"Under this framework, the observed southward ITCZ migration in recent decades is consistent with, but does not uniquely imply, an ongoing weakening of the AMOC."

"Because direct observations of the AMOC are limited to the post-2004 period, the long-term variability of AMOC beyond 20 years relies mostly on AMOC proxies, such as the SST contrast between subpolar North Atlantic and the global mean[37], sea surface salinity[55], surface heat flux[56] etc. These proxies are subject to substantial uncertainty and may reflect multiple physical processes beyond changes in overturning strength alone[30,56-58]. Given that AMOC weakening is one of several contributing mechanisms to the southward ITCZ movement, does the observed ITCZ shift southward imply an overall AMOC decline over the last several decades?"

"Consistent with this interpretation, an independent AMOC proxy from ref 59 also indicates a slight decline in recent decades. As we have shown in the model experiments, significant AMOC weakening leads to a southward ITCZ movement in both CESM2 and EC-Earth3. This long-term overall ITCZ southward movement in the recent 80+ years derived from multiple reanalysis products could be a sign of overall weakening of the AMOC in this period. Although a more definitive demonstration of this relationship for observations still needs more work, we give the following indirect evidence."

"We further propose that the observed southward ITCZ migration in the reanalysis products could serve as a proxy for long-term AMOC weakening (decadal or longer timescales), supported by the concurrent slowdown in Arctic sea ice decline and intensification of the NAWH. Given the ongoing AMOC weakening and CMIP6 projections[67] since 2005, it is plausible that the ITCZ will continue to shift further southward in the coming decades. If the AMOC would weaken more dramatically, the ITCZ could jump to the equator and greatly alter global precipitation patterns by reorganizing the atmospheric and oceanic circulations, resulting in far reaching impacts on the Earth's climate."

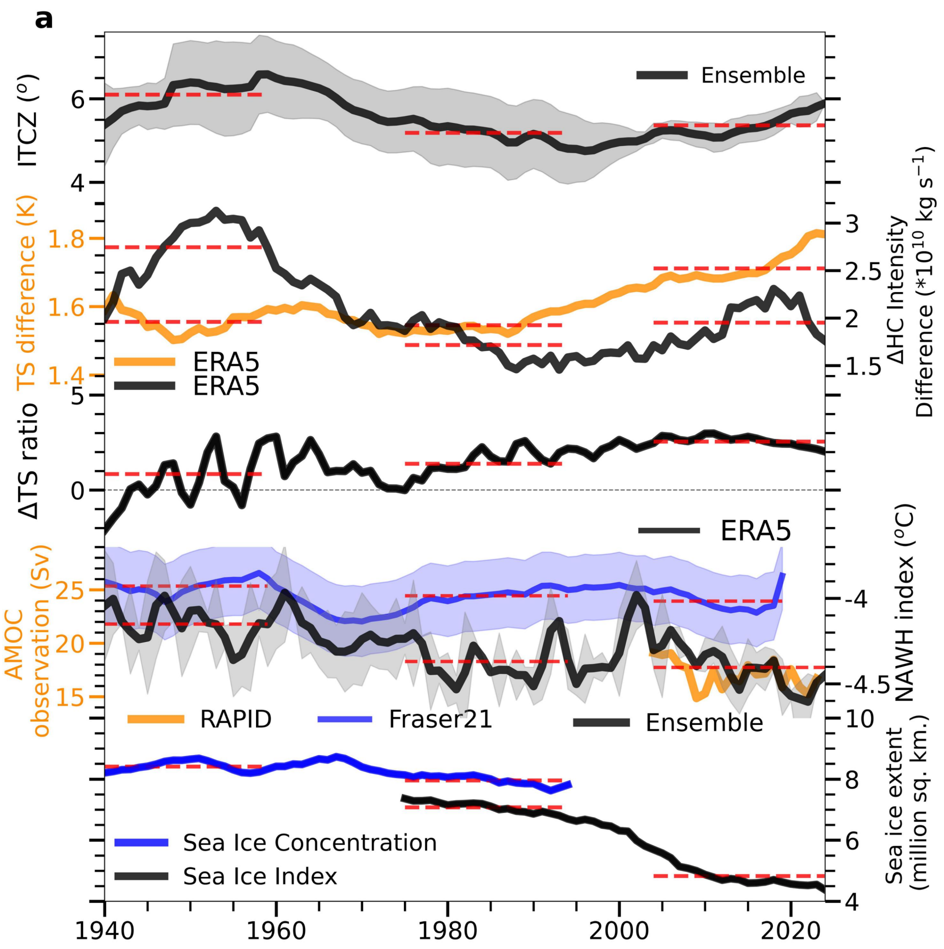

Fig. 5 | ITCZ, TS and HC intensity differences, Arctic amplification ratio, AMOC observation and proxy, NAWH index, and sea ice extent. 11-year running mean of ITCZ location ensemble derived from ERA5, JRA-55, NCEP, and ERA-20C (the first line from top to bottom), TS and HC intensity differences (the second lines), and Arctic amplification ratio (the third line) from ERA5 data. The AMOC observation at 26.5oN, AMOC proxy (Fraser21) from ref 59, and the ensemble of the 11-year running mean NAWH indices from the Extended Reconstructed Sea Surface Temperature (ERSSTv5) and Hadley Centre Sea Ice and Sea Surface Temperature dataset (HadISST; the fourth lines), with shading indicating one standard deviation. The 11-year running mean sea ice extent derived from sea ice concentration data (the fifth line; orange) and satellite-based observation (the fifth line; black). The red lines represent the averages over the periods 1940-1959, 1975-1994, and 2005-2024.

Other comments:

1. “North” and “South” group labels sound like they are referring to mean position. When reading the paper it is clear that they refer to changes in ITCZ position, but it is worth thinking about labels that would be clearer. Maybe “Northward” and “Southward” would be better.

Response: Thank you for this insightful comment. In response to this suggestion, we have

renamed the "North" and “South” groups as the “Northward” and “Southward” groups throughout the main text and figure captions, including the Extended Data, to more clearly indicate that these labels refer to changes in ITCZ position rather than its mean location. For example (for example, Lines 124-127),

“Thus, we classify the models into three groups based on the direction of ITCZ shifts: Northward (red numbers), Southward (blue numbers), and Neutral (gray numbers) Groups where the big solid circles represent the ensemble mean of each group (Fig. 1).”

2. The AMOC changes significantly in the “fixed AMOC” experiments. I understand the point is to keep the ITCZ in the same place, which it does successfully, but it might make sense to rename them because Fig. 4e clearly shows the AMOC changing.

Response: Thank you for this helpful comment. We agree that the original term “fixed AMOC” was misleading, as the AMOC is not held constant in these experiments, as illustrated in Fig. 4e. The intention of these experiments is instead to constrain the ITCZ position under conditions of relatively weaker or stronger AMOC.

To avoid confusion, we have renamed these experiments throughout the manuscript as 1%CO_2_fxITCZWK and 1%CO_2_fxITCZST, respectively, to more accurately reflect their experimental design (Lines 278-283, 297-298, 309-313).

“In addition to the Control simulation, we conducted five additional experiments, including Thermal/CO_2 with 150-year 1%CO_2 and 300-year extension at fixed CO_2 quadrupling, Haline/Hosing with 150-year ramping up hosing and 300-year extension with fixed hosing, ThermalHaline representing a combination of both as well as 1%CO_2_fxITCZWK and 1%CO_2_fxITCZST with 150-year 1%CO_2 under artificially constrained relatively weak and strong AMOC states, respectively (Methods).”

“The 1%CO_2_fxITCZWK and 1%CO_2_fxITCZST are designed to test whether moderate AMOC weakening or strengthening can influence ITCZ migration.”

“In the 1%CO_2_fxITCZWK and 1%CO_2_fxITCZST simulations, more rapid sea ice decline due to the sustained AMOC in the first half of the experiments with moderate weakening or strengthening in the second half of the experiments drives much stronger TS contrast across all latitude bands and a much stronger polar amplification.”

3. The word variability is used on line 91 (and possibly other places), how is this defined?

Response: Thank you for pointing this out. We agree that the term “variability” was used too loosely in the original manuscript and could lead to ambiguity. We have now clarified its definition at each occurrence in the revised text.

Specifically, on Lines 129-132, “variability” refers to the standard deviation of the diagnosed ITCZ position, which is now explicitly stated in the text. On Lines 425-427, “variability” denotes decadal-to-multidecadal fluctuations in AMOC strength, rather than short-term variability or a linear trend. On Lines 429-430, “variability” refers to the variations of the SST

difference between the subpolar North Atlantic (45°-65°N, 50°-20°W) and the broader extratropical North Atlantic (30°-65°N, 70°-10°W).

To avoid further confusion, we have revised the text to explicitly define "variability" at each location and, where appropriate, replaced the term with more specific statistical descriptors (Lines 129-132, 425-427, 429-430).

"Moreover, the standard deviation of the ITCZ position is much larger for Northward Group (1.60 $\pm$ 0.49°) than other groups (1.14 $\pm$ 0.38° for Neutral Group and 0.82 $\pm$ 0.23° for Southward Group), and this standard deviation is 0.86° for ERA5, 0.79° for JRA-55, 1.03° for NCEP, and 0.87° for ERA-20C."

"A key indicator of AMOC variation is the North Atlantic warming hole (NAWH)[30,37]. A more negative NAWH index (Methods)[60] corresponds to a cooler subpolar North Atlantic and a weaker AMOC[30,37,61]."

"Although multiple drivers could influence NAWH variations[62], AMOC remains the most significant driver[30-31,33,38,63]."

Minor comments:

1. The captions are indistinguishable from the main text. This will obviously be changed if the article is published, but it would be easier to review if the captions were slightly smaller or otherwise set apart.

Response: Thank you for this comment. We agree that the figure captions will be more clearly distinguished from the main text in the final published version following the journal's formatting. We have also ensured that the captions are clearly separated from the main text in the revised manuscript to facilitate the review process.

2. Line 251: "mean longitudinal distribution of precipitation" -- is this meant to say the zonal mean? Or precipitation as a function of latitude?

Response: Thank you for pointing this out. Here, we intended to refer to the zonal-mean precipitation, expressed as a function of latitude. To avoid ambiguity, we have revised the sentence accordingly (Lines 386-387).

"Compared to the Northward Group, the global zonal-mean precipitation, especially a notable precipitation reduction near 9°N in CESM2 and the Southward Group more closely resembles ERA5."

- **<u>Reviewer #1</u>**

Reviewer #1 (Remarks to the Author):

The authors’ extensive new analyses satisfactorily address my prior concerns and provide compelling evidence for the robustness of the main conclusions. The manuscript has been substantially strengthened through revision. I have no further comments and recommend acceptance in its current form.

Response: We sincerely thank the reviewer for the positive feedback and for the helpful comments during the revision process, which have significantly improved the quality of our manuscript. We are pleased that the reviewer is satisfied with our new analyses and our responses to the previous concerns.

- ## Reviewer #2

Reviewer #2 (Remarks to the Author):

The authors have made a substantial effort to address the reviewer comments, and the manuscript has improved significantly in clarity, structure, and physical interpretation.
Importantly, my primary concern regarding the strength of the AMOC-related conclusions has been adequately addressed. The revised manuscript now presents a more balanced and appropriately cautious interpretation, clearly distinguishing between consistency and causality.
The additional analyses on basin-scale ITCZ behaviour and the expanded discussion of mechanisms further strengthen the study.
Some minor concerns remain regarding the treatment of model biases (e.g., double-ITCZ) and the extent to which their influence has been fully quantified. However, these do not undermine the main conclusions.

Overall, I consider the manuscript suitable for publication after minor revision.

Response: We thank reviewer #2 for this insightful comment. To more rigorously quantify the influence of double-ITCZ biases, we have decomposed the zonal-mean precipitation bias into antisymmetric and symmetric components following Ren and Zhou (2024). The root mean square error (RMSE) of the antisymmetric bias, which directly reflects the double-ITCZ bias, is 0.293 mm day^{-1} for the Northward Group—approximately three times larger than that of the Southward Group (0.097 mm day^{-1}). In contrast, the symmetric bias RMSE is nearly identical between the two groups (0.705 vs. 0.697 mm day^{-1}), confirming that the key difference lies specifically in the double-ITCZ component. While this stronger antisymmetric bias in the Northward Group may contribute to its more equatorward mean ITCZ position, the similar symmetric bias structure across both groups supports our conclusion that the sign of ITCZ migration is primarily controlled by coupled AHT_{eq} and OHT_{eq} changes rather than model biases. We have added this quantitative analysis to the revised manuscript (Lines 468-481).

"To quantify this difference, we decompose the zonal-mean precipitation bias into antisymmetric and symmetric components following ref 71. The antisymmetric bias—which directly reflects the interhemispheric precipitation asymmetry and is most relevant to double-ITCZ bias—has an root mean square error (RMSE) of 0.293 mm day^{-1} for the Northward Group, approximately three times larger than that of the Southward Group (0.097 mm day^{-1}). In contrast, the symmetric bias RMSE is nearly identical between the two groups (0.705 mm day^{-1} for Northward Group vs. 0.697 mm day^{-1} for Southward Group), confirming that the key difference lies in the antisymmetric (double-ITCZ) component rather than the overall precipitation magnitude. The stronger antisymmetric bias in the Northward Group, particularly over 5°S—15°S where the mean bias reaches -1.34 mm day^{-1} compared to -0.96 mm day^{-1} in the Southward Group, could contribute to a more equatorward mean ITCZ position[72], which may in turn influence the ITCZ response to rising greenhouse gas forcing. Nevertheless, since both groups share a similar symmetric bias structure, the contribution of the double-ITCZ bias to the sign of ITCZ migration is likely minor."

● Reviewer #3

We thank Reviewer #3 for the thorough and constructive comments, which have significantly improved the clarity, rigor, and consistency of our manuscript. Below we provide a point-by-point response to each comment.

First, regarding overstated conclusions, we have moderated our language throughout the manuscript in three key areas: (1) we clarified that the three quasi-stable ITCZ positions identified in Fig. 4c are specific to our forcing scenarios and acknowledged that other quasi-stable states may exist under different conditions; (2) we extensively revised our interpretation of the historical period by explicitly acknowledging that the relative contributions of internal variability, aerosol forcing, and CO_2 forcing cannot be definitively separated from the observational record, and we now treat historical observations as qualitative context only; and (3) we removed the statement proposing ITCZ migration as a proxy for AMOC weakening. We also revised the statement regarding OHT_{eq} to acknowledge that it can act as an active forcing under non-equilibrium conditions such as AMOC weakening, rather than being a simple response to AHT_{eq}.

We hope these revisions can directly address the reviewer's concerns regarding overstated conclusions in certain parts of the manuscript, insufficient physical explanation, and presentation choices. We also hope that these revisions have substantially enhanced the rigor, transparency, and interpretability of the manuscript. Below is the detailed point-by-point responses to each comment.

Reviewer #3 (Remarks to the Author):

Summary:

The new version of this paper is much improved from the previous and it is well on its way to being publishable. However, I still think that there are overstated conclusions, though these are now scattered throughout the text rather than in the title or abstract, and that this version has many typos that need to be fixed. What I believe is the manuscript's main contributions, that 1. ITCZ position and ITCZ shifts under warming are strongly anti-correlated, and 2. That the former is generally correlated with atmospheric mechanisms while the latter is correlated with AMOC changes, are scientifically interesting and important. The text should be written such that the focus is on these conclusions.

Major comments:

1. Overstated conclusions -- Rather than focusing on the interesting and supported conclusions mentioned above, the manuscript often makes somewhat unrelated strong statements which are not supported by the evidence:

(1) Line 365 states that Figure 4c "suggest[s] the existence of three possible quasi-stable

ITCZ positions." I believe what the authors are saying is that the states reached by year 400 of the simulations are quasi-stable. This conclusion seems reasonable to me, and it could be said that "these three ITCZ positions are quasi-stable." However, by stating there are three possible positions, the authors seem to be implying that any other ITCZ positions, e.g. ones between -2 and -6, would not be stable. I do not believe there is evidence of this. If the AMOC strength increased slightly, would the ITCZ move slightly north? Are the authors trying to say that these three ITCZ positions are special in some way? Why would this be?

Response: We thank the reviewer for this insightful comment. We agree that our original phrasing "three possible quasi-stable ITCZ positions" could be misinterpreted as implying that ITCZ positions between these three states (e.g., between -2° and -6°) would be unstable, which is beyond what our simulations can demonstrate. This result might be model dependent and this is true for our model simulation after year 400 as mentioned by reviewer #3. However, even so, we could not totally rule the possibility that if the forcing condition changes, the quasi-equilibrium states will be the same for CESM2. We have further clarified this in our revision.

Our simulations only examine three specific forcing scenarios, and the three quasi-stable positions identified reflect the outcomes of these particular experiments rather than an exhaustive characterization of all possible quasi-stable ITCZ states. We have therefore modified this sentence and added a clarification that these three positions are the quasi-stable states obtained under our specific forcing scenarios. We also added a sentence acknowledging that other quasi-stable ITCZ positions might exist under different forcing conditions, such as varying freshwater flux strength over the subpolar North Atlantic, and noted that this remains to be explored in future work (Lines 319-326).

"Based on this, the results shown in Fig. 4c suggest the existence of three quasi-stable ITCZ positions in CESM2 after year 400 under the three specific forcing scenarios examined in this study: around 6°N under preindustrial conditions with an active AMOC, near the equator when the AMOC collapses under strong greenhouse gas forcing, and around 6°S when the AMOC collapses due to freshwater forcing alone. It is worth noting that other quasi-stable ITCZ positions might exist under different forcing conditions and different AMOC stabilization levels associated with the respective forcing conditions. Moreover, the exact quasi-equilibrium position may also be model dependent which remains to be explored in future work."

(2) Internal variability vs. forced response in the historical period

- Lines 404-406 states that because 25% is less than the CO_2 increase in the forced experiments, ITCZ variations "are primarily modulated by internal variability." Just because CO_2 changes are smaller than that of an artificial experiment does not mean that observed variations are modulated by internal variability, as there is still some forcing. We do not know whether the historical ITCZ movement is variability or forced

without more evidence.

Response: Thank you for this helpful comment. We agree that a smaller CO_2 increase relative to our idealized experiments does not necessarily imply that the observed ITCZ variations are primarily driven by internal variability, as external forcings could still play important roles. We have therefore revised the text to remove the claim that internal variability is the primary driver and instead acknowledge that the relative contributions of internal variability and forced response during the historical period remain uncertain (Lines 359-371).

"During this period, the atmospheric CO_2 concentration increases by about 41% (from ~300 to 422 ppmv) but is still significantly less than the CO_2 increase in the period analyzed here (around 4 times of preindustrial level) in the 1%CO_2 experiments. This suggests that although the trend of observed ITCZ movement could be induced by rising greenhouse gas forcing, the observed interdecadal ITCZ variations must be a combination of the external forcings and the internal variability. However, our current analysis cannot disentangle the exact percentage contribution of the different external forcings and internal variability on the observed ITCZ variability and trend in recent 80+ years remain uncertain which warrants further investigation. For example, aerosol forcing is confirmed to be an important driver for the southward migration of the tropical precipitation in the late 20th century[59,60], which probably corresponds to ITCZ change from the 1940-60s period to the 1990s (Fig. 5). Nevertheless, despite the uncertainty in separating forced response from internal variability during the historical period, the observed ITCZ variations under moderate CO_2 rise may still offer a qualitative context for understanding potential ITCZ responses under stronger future CO_2 forcing."

- The following sentences, despite the above statement, act as if Lines 404-406 are exactly wrong and treat the historical period as a forced response. This is also not necessarily true, as we do not know to what extent ITCZ movement is variability or forced.

Response: We thank the reviewer for identifying this inconsistency. We acknowledge that our subsequent sentence contradicted our earlier statement by treating the historical ITCZ variations as a purely forced response.

You are correct that we cannot determine the extent to which the historical ITCZ movement reflects forced response versus internal variability based on our current analysis. We have therefore revised both statements to maintain consistency. Specifically, we no longer claim that internal variability is the primary driver of historical ITCZ variations, nor do we treat the historical period as a clean forced response. Instead, we acknowledge the uncertainty explicitly and reframe the historical period as providing only a qualitative reference context, rather than a definitive constraint on model evaluation under future CO_2 forcing. We have revised our argument and make it more consistent (Lines 359-371).

"During this period, the atmospheric CO_2 concentration increases by about 41% (from ~300

to 422 ppmv) but is still significantly less than the CO_2 increase in the period analyzed here (around 4 times of preindustrial level) in the 1%CO_2 experiments. This suggests that although the trend of observed ITCZ movement could be induced by rising greenhouse gas forcing, the observed interdecadal ITCZ variations must be a combination of the external forcings and the internal variability. However, our current analysis cannot disentangle the exact percentage contribution of the different external forcings and internal variability on the observed ITCZ variability and trend in recent 80+ years remain uncertain which warrants further investigation. For example, aerosol forcing is confirmed to be an important driver for the southward migration of the tropical precipitation in the late 20th century[59,60], which probably corresponds to ITCZ change from the 1940-60s period to the 1990s (Fig. 5). Nevertheless, despite the uncertainty in separating forced response from internal variability during the historical period, the observed ITCZ variations under moderate CO_2 rise may still offer a qualitative context for understanding potential ITCZ responses under stronger future CO_2 forcing."

- The historical period is likely strongly forced by aerosols as well as CO_2, this is not addressed and probably invalidates several of the paper's Statements.

Response: We thank the reviewer for raising this important point. We agree that aerosol forcing is a significant driver of historical ITCZ variations and should not be overlooked. Specially, aerosol forcing is proven to be the primary driver of the southward migration of the tropical precipitation in the late 20th century (Hwang et al. 2013; Allent et al. 2015). This implies that the southward ITCZ movement of more than 1° latitude from the 1940-60s period to the 1990s is probably primarily driven by the aerosol forcing (Fig. 5). We emphasize that our primary conclusions are based on the model experiments, which explicitly isolate the CO_2-forced response. The historical observations are retained as qualitative context only, with the explicit caveat that aerosol forcing, CO_2 forcing, and internal variability are not separable in the observational record. So our interpretation of the historical period is limited.

We have revised the relevant sections to explicitly acknowledge the role of aerosol forcing in historical ITCZ variations, and to clarify that the historical period cannot be straightforwardly used as an analog for CO_2-forced ITCZ changes. We now treat the historical observations as providing only qualitative context, with the caveat that aerosol forcing, CO_2 forcing, and internal variability are not separable in our current analysis (Lines 366-368, 388-392, 406-409).

"For example, aerosol forcing is confirmed to be an important driver for the southward migration of the tropical precipitation in the late 20th century[59,60], which probably corresponds to ITCZ change from the 1940-60s period to the 1990s (Fig. 5)."

"This long-term overall ITCZ southward movement in the recent 80+ years derived from multiple reanalysis products is consistent with an overall weakening of the AMOC in this period, although aerosol forcing has also been identified as a significant contributor to the

southward tropical precipitation shift during the late 20th century[59,60], and the relative contributions of AMOC weakening, aerosol forcing, and internal variability cannot be definitively separated from the historical record alone."

"Thus, the overall southward ITCZ movement shown in the reanalysis products is broadly consistent with a slowdown of the AMOC in the last few decades, though we acknowledge that aerosol forcing and internal variability likely also contribute, and a definitive attribution remains beyond the scope of this study."

- Lines 408-413 states that a lack of perfect correlation between TS contrast and ITCZ position imply a role for the ocean. I do not believe this is true given the possible role of internal variability and aerosols. It would have to be established that the correlation between TS contrast and ITCZ is perfect in the absence of ocean forcing to support this statement.

Response: We thank the reviewer for this important critique. We agree that the lack of perfect correlation between TS contrast and ITCZ position does not necessarily imply a role for the ocean, as internal variability and aerosol forcing could equally explain this discrepancy. Establishing that the correlation would be perfect in the absence of ocean forcing would indeed require a dedicated set of model experiments that isolate each forcing factor, which is beyond the scope of the current study.

We have therefore revised the text to remove the causal implication and replace it with a more cautious statement. Rather than claiming that the imperfect correlation "implies a role from the ocean," we now state that the result is "consistent with a potential role of oceanic forcing," while explicitly acknowledging that internal variability and aerosol forcing may also contribute to the observed discrepancy in the revised text (Lines 372-378).

"Fig. 5 also shows that the HC intensity contrast experiences similar changes as the ITCZ, but the TS contrast was nearly unchanged from the 1940s to 1990s and increased in recent 20+ years, which aligns with the polar amplification measured by the ratio of polar to global mean temperatures (ΔTS ratio). This imperfect relationship between historical interdecadal ITCZ variations and TS contrast changes may reflect the complicated interactions among different external forcings and the internal variabilities in controlling this relationship. One of these processes which includes both externally forced response and internal variability is AMOC as suggested below."

(3) Lines 499-500 still seem to say that we should use ITCZ migration as a proxy for AMOC weakening. Proxies are useful when the two quantities are well correlated across a long time period or with respect to the change in question. This is simply not the case for AMOC strength and the ITCZ, either in observations (where the three time periods do not even have

the same relative sign in the two quantities) or in simulations (where a completely collapsed AMOC in CESM2 can lead to three different ITCZ positions). This should be removed or better supported.

Response: Thank you for this helpful comment. We agree that proposing ITCZ migration as a proxy for AMOC weakening is not well supported, and in fact contradicts our own results. As the reviewer correctly points out, our CESM2 simulations show that a completely collapsed AMOC can lead to three different ITCZ positions depending on the forcing mechanism, and the observed relationship between AMOC strength and ITCZ position is not consistent across the three time periods shown in Fig. 5. These results clearly demonstrate that AMOC strength and ITCZ position do not have a stable, well-correlated relationship, which is a fundamental requirement for a reliable proxy.

We have therefore removed the proxy statement and replaced it with a more cautious interpretation. We now state that the concurrent southward ITCZ migration, slowdown in Arctic sea ice decline, and intensification of the NAWH are consistent with ongoing AMOC weakening, without claiming that ITCZ migration can serve as a proxy (Lines 455-459).

"While our results suggest a physical linkage between AMOC weakening and southward ITCZ migration under specific forcing conditions, the relationship between AMOC strength and ITCZ position is not sufficiently consistent across different forcing scenarios and time periods to serve as a reliable proxy. The concurrent southward ITCZ migration, slowdown in Arctic sea ice decline, and intensification of the NAWH are nonetheless consistent with ongoing AMOC weakening on decadal timescales."

2. Abundance of typos -- There are enough typos throughout the paper for it to be distracting. I'm sure I missed some (especially later on in the paper), but here are the ones I found:

(1) Line 30 -- "It is closely linked to Hadley Cell (HC)" -- should be "the Hadley Cells";

(2) Line 56 -- "Equilibrate conditions" instead of "Equilibrium conditions";

(3) Line 62 -- "Continuously warming world" not "Continuous warming world";

(4) Line 96 -- "are multiply ways" should be "multiple ways;

(5) Line 120 -- "19934" as a year;

(6) Line 150 -- "Such as" should be "As such";

(7) Line 168-171 -- refers to "the correlations" between changes in variables "becoming stronger". These are correlations between changes in variables, so I assume they are not becoming stronger, they are just strong.

Response: Thank you for this insightful comment. These typos and others have been modified throughout the paper.

Other comments:

1. Line 18 is hard to read, how about just saying "Earth system model simulations" without mentioning the "warmer background base state at this point"?

Response: Thank you for this insightful comment. The phrase "with a warmer background base state" makes Line 18 harder to read. We have removed this phrase and now simply refer to "Earth system model simulations," with the details of the model configuration described in the Methods section (Lines 17-21).

"Using reanalysis data, Earth system model simulations, and targeted sensitivity experiments, we assess risks of the ITCZ crossing any thresholds and find only about one-third of CMIP6 models with a more northern ITCZ mean position —closer to the observed climatological state —simulate a southward ITCZ shift consistent with recent observations."

2. The abstract is focused on models with a more northern ITCZ mean position -- it needs to be explained at least briefly in the abstract that this is because those are closer to reality.

Response: We thank the reviewer for this suggestion. We have added a brief clarification in the abstract to explain that models with a more northern ITCZ mean position are selected because they are closer to the observed climatological state (Lines 17-21).

"Using reanalysis data, Earth system model simulations, and targeted sensitivity experiments, we assess risks of the ITCZ crossing any thresholds and find only about one-third of CMIP6 models with a more northern ITCZ mean position —closer to the observed climatological state —simulate a southward ITCZ shift consistent with recent observations."

3. Line 55-56 currently says "Based on this theory, changes in oceanic meridional heat transport across the equator are a simple response to the changes in AHT_eq as they covary under equilibrate conditions and are mechanically coupled over low latitudes":

I would disagree with this statement. The energy flux theory for ITCZ position leaves open the possibility of OHT as a forcing which can move the ITCZ, and many papers have explored the impact of such OHT.

Response: Thank you for pointing this out. We agree that our original phrasing "OHT_{eq} is a simple response to AHT_{eq}" was overly restrictive. As the reviewer correctly points out, the theory leaves open the possibility of OHT acting as an active forcing that can drive ITCZ displacement, and numerous studies have demonstrated this role—particularly in the context of AMOC variability altering northward oceanic heat transport.

We acknowledge that our original statement also contradicts the central argument of our paper, which demonstrates that AMOC weakening—through its effect on OHT_{eq}—drives southward ITCZ migration. We have therefore revised the text to clarify that OHT_{eq} and AHT_{eq} covary under equilibrium conditions and are mechanically coupled, while also acknowledging that OHT_{eq} can act as an active forcing under non-equilibrium conditions such as AMOC weakening (Lines 55-60).

"Based on this theory, changes in AHT_{eq} and oceanic meridional heat transport across the equator (OHT_{eq}) together determine the position of the energy flux equator and thus the ITCZ. While AHT_{eq} is the dominant driver under quasi-equilibrium atmospheric conditions, OHT_{eq} can also act as an active forcing—for example, AMOC weakening reduces northward OHT_{eq}, requiring a compensating reduction in southward AHT_{eq} and thus a southward ITCZ displacement[7,8]. Under equilibrium conditions, OHT_{eq} and AHT_{eq} covary[19,20] and are mechanically coupled over low latitudes[21-24]."

4. The phrase "Warmer background base state" can be confusing because it is combini n "base state," which sounds like the control, and "warmer." I think it would be clearer to just say "warmer climate" when possible.

Response: Thank you for pointing this out. We agree that "warmer background base state" could be ambiguous. In the revised manuscript, we have replaced it with "warmer climate" throughout the text (Lines 77-81).

"... the ECMWF Twentieth Century Reanalysis (ERA-20C)[45], model experiments with a warmer climate (represented here by the idealized 1% CO_2 experiments (hereafter simply 1%CO_2) from the Coupled Model Intercomparison Project Phase 6 (CMIP6)), and a suite of specially designed sensitivity experiments using the Community Earth System Model version 2 (CESM2; Methods)[46] and the EC-Earth3 model[47]."

5. Lines 96-113 is essentially a caveat and would be better if it were much shorter, in the methods section, or in an appendix. Having it at the beginning of a section which sounds like a results section distracts from the results.

Response: We thank the reviewer for this suggestion. In the revised manuscript, we have condensed this passage in the main text to briefly justify our choice of ITCZ definition, and moved the detailed comparison between the streamfunction-based and precipitation-based ITCZ metrics to the Methods section (Lines 98-105).

"There are multiple ways to define the ITCZ—precipitation-based methods (e.g., the latitude of maximum precipitation[48], the precipitation centroid[49], the brightness temperature[50]), and the atmospheric circulation based methods (e.g., vertical pressure velocity[51], and the boundary between northern and southern HCs[41]). Although the choice of ITCZ definition can

affect the absolute ITCZ position and its response to external forcing (Supplementary Fig. 1), it may not affect the interpretation of the underlying physical processes. Because we will use the atmospheric energy constraint theory to explain the physical processes, we therefore adopt an atmospheric circulation-based ITCZ definition in this study[41] as this energy constraint theory provides a close relationship between HCs and AHT_{eq}.*"*

6. Line 129 should clarify these are standard deviations with respect to time, rather than across models.

Response: We thank the reviewer for this clarification. We have added "temporal" to specify that the standard deviation refers to variability with respect to time, not across models (Lines 121-124).

"Moreover, the temporal standard deviation of the ITCZ position is much larger for Northward Group (1.60±0.49°) than other groups (1.14±0.38° for Neutral Group and 0.82±0.23° for Southward Group), and this temporal standard deviation is 0.86° for ERA5, 0.79° for JRA-55, 1.03° for NCEP, and 0.87° for ERA-20C."

7. In scatter plots which show all models, the mean dots could be more prominent. I would recommend either fading out the individual models somewhat or putting an outline (black circle) on the ensemble mean points. Reanalysis products should probably be represented by points, not numbers.

Response: We thank the reviewer for these suggestions. In the revised figures, we have added a black outline to the ensemble mean points to make them more prominent. We have also replaced the numerical labels for reanalysis products with filled circles, with the reanalysis datasets identified in the legend instead. These changes have been applied consistently across all relevant figures.

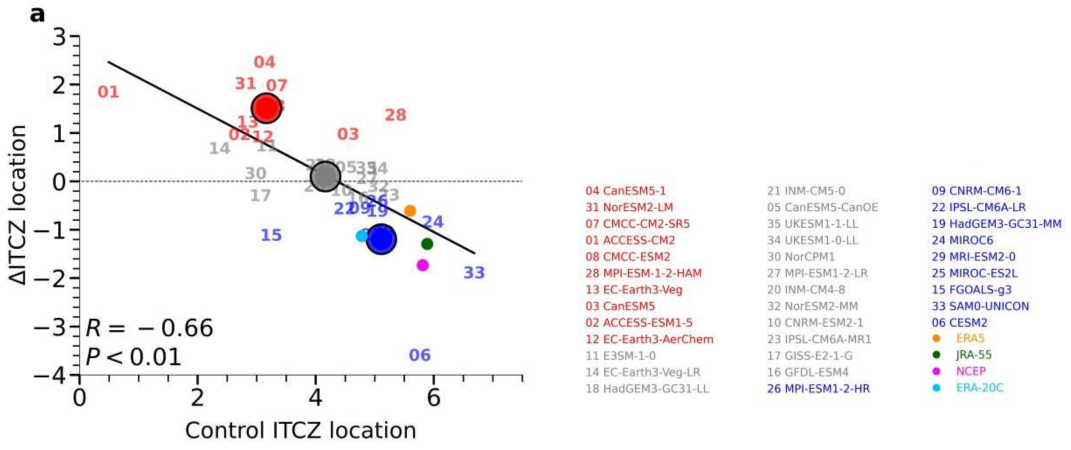

Fig. 1 | Relationships in the ITCZ location and ITCZ migration. a, Relationships across CMIP6

models between climatological mean ITCZ position and ITCZ migration from the preindustrial control (hereafter "Control") to the late-stage response of the CMIP6 1%CO_2 experiment, defined as the mean over years 131-150, when atmospheric CO_2 reaches approximately four times its preindustrial level. The historical ITCZ location based on ERA5 (orange dot), JRA-55 (green dot), NCEP (magenta dot), and ERA-20C (cyan dot) are approximately 5.50°N over 1940-2024, 5.89°N over 1958-2023, 5.81°N over 1948-2023, and 4.78°N over 1940-2010. The ITCZ migration is calculated as the ITCZ difference from the early period (1940-1969, 1958-1987, 1948-1977, and 1940-1969) to the recent period (1995-2024, 1994-2023, 1994-2023, and 1981-2010). The Northward, Southward, and Neutral Group ensembles are shown separately. One standard deviation for each model group is shown.

8. Does it make physical sense to choose the "most consistent and realistic" models (132-134) based on changes with respect to warming rather than mean position and standard deviation?

Response: Thank you for this helpful comment. It is not straightforward to directly compare the historical ITCZ migration with the 1%CO_2 response, as the drivers of the observed historical migration remain uncertain (as acknowledged in our response to above comment regarding internal variability vs. forced response). Nevertheless, we note that the Southward Group is consistent with observations in terms of both mean ITCZ position and variability, and the sign of its CO_2-forced ITCZ response (southward) is also consistent with the observed historical migration direction. We therefore retain "migration" in this statement but have revised the text to explicitly acknowledge the uncertainty in attributing the historical migration, avoiding any implicit claim of mechanistic equivalence across different forcing magnitudes (Lines 124-128).

"Overall, the Southward Group best captures the observed ITCZ mean state and variability. The southward ITCZ migration observed in reanalysis is also consistent with the southward response of the Southward Group in response to CO_2 quadrupling, although the drivers of the historical ITCZ southward migration may differ from those driving the CO_2-forced response."

9. Line 147 -- "Control" hasn't been defined in the text yet, only in the caption.

Response: We thank the reviewer for catching this. We have defined "Control" at its first occurrence in the main text (Lines 130-131).

"We first analyze the equilibrium-climatological mean ITCZ location in preindustrial control (hereafter "Control"; Fig. 2)."

10. Is the initial northward shift of the ITCZ in the southward group statistically significant?

Response: We thank the reviewer for this question. We performed a one-sample t-test to assess whether the initial northward ITCZ shift is significantly different from zero. The northward shift during the first 20 years is not statistically significant in either group (Northward Group: +0.27 $\pm$ 0.49°, p = 0.116; Southward Group: +0.09 $\pm$ 0.21°, p = 0.207), consistent with our use of the term "slight northward displacement" in the text. We have added this clarification to the revised manuscript (Lines 208-211).

"During the first 20 years of the 150-year 1%CO_2 experiment, both the Northward and Southward Groups simulate a slight northward ITCZ displacement (Fig. 4a), following previous findings[4], though this initial shift is not statistically significant in either group."

11. Line 330-331 -- It is not necessary to mention temporary data availability issues.

Response: We thank the reviewer for this suggestion. We have removed this sentence from the revised manuscript.

12. In extended Data Fig. 2b, the caption should say the correlation coefficient with model 15 included. It is not an outlier for the changes, only for the control.

Response: We thank the reviewer for this careful observation. We agree that FGOALS-g3 (model 15) is an outlier only for the climatological mean AMOC and ITCZ position (panels b-c), but not for the changes (panels e-f). We have revised the caption to clarify this.

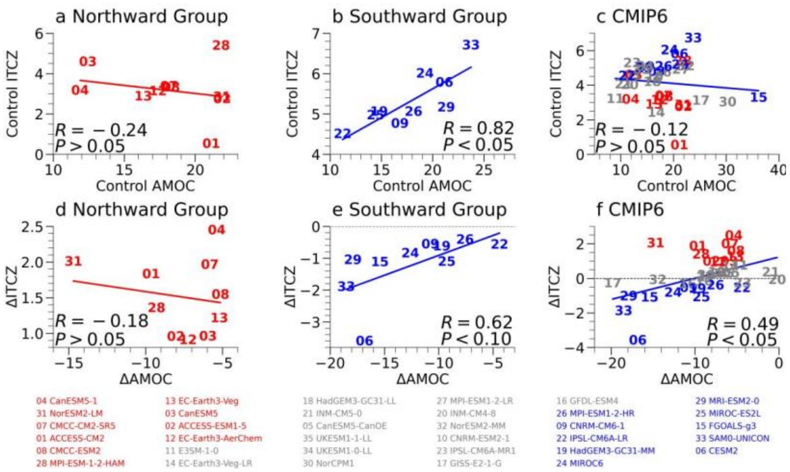

Supplementary Fig. 2 | Relationships between the AMOC and the ITCZ across CMIP6 models. a-c, Intermodel relationships between the climatological mean AMOC strength and ITCZ position for the Northward Group (**a**), Southward Group (**b**), and all CMIP6 models (**c**). **d-f**, Same as **a-c**, but showing the relationship between AMOC weakening and ITCZ migration between CO_2 quadrupling (mean over years 131-150 of the 1%CO_2 experiment) and the preindustrial control simulation. For each panel, the Pearson correlation coefficient (**R**) and the **P** value from a linear

regression (black solid line) are shown. The FGOALS-g3 model (label 15) is excluded as an outlier in panels **b-c** (climatological mean AMOC and ITCZ position), but is included in panels **e-f** (AMOC weakening and ITCZ migration).

Review of “Intertropical Convergence Zone at Risk of Crossing a Tipping Point Driven by Ocean Circulation Changes in a Warming Climate”

The ITCZ, a planetary scale band of precipitation in the deep tropics, controls much of the world’s rainfall. It shifts North and South on seasonal and longer timescales and this movement is important to understand in the present and future. The ITCZ is associated with the ascending branch of the Hadley circulation and the atmospheric energy flux equator, leading to tropical control over ITCZ position through the exact position of ascent and extratropical control over ITCZ position through an energy balance perspective. One of the major extratropical influences on ITCZ position is the Atlantic Meridional Overturning Circulation (AMOC), which transports energy to the North in the Atlantic basin, shifting the ITCZ northward.

In the manuscript “Intertropical Convergence Zone at Risk of Crossing a Tipping Point Driven by Ocean Circulation Changes in a Warming Climate,” Guo et al. discuss how the ITCZ will shift in a warming climate, emphasizing the role of ocean circulation, and, in particular, changes in the AMOC. The paper contains several interesting results, including 1. that AMOC weakening controls intermodel differences in ITCZ shifts but not pre-industrial ITCZ position, and 2. that warming alone without a weakening AMOC cannot explain the southward ITCZ shifts under climate change. However, the results are significantly overstated throughout the paper, especially in the discussion and title. Additionally, there are several unclear presentation choices and gaps in physical understanding in the current version which should be fixed if the paper is to be published.

Major Comments

- **Overstated Conclusions:**There are several conclusions the authors seem to have which are not convincingly demonstrated. These are problematic to a varying degree, listed below:
  - **Tipping Point:** I see little to no evidence of a tipping point in the data displayed. There is certainly not enough evidence for the title of the manuscript. The concept of a tipping point is introduced in the section starting on line 236, but is not defined carefully. Most reasonable definitions of a tipping point refer to an irreversible, sudden, and large change in response to a relatively small change in forcing. The reversibility of the ITCZ shift is not tested, the changes don’t seem particularly sudden, and ITCZ changes are not disproportionately large compared to the fractional change in AMOC strength (Fig. 4e). Additionally, there are no two experiments which show the same final ITCZ position after several hundred years, so I don’t see how the authors can conclude that there are “three quasi-stable ITCZ positions.” To substantiate the claim of a tipping point, the

authors need to rigorously define the concept referring to past work and show that their simulations prove the existence of such a change.

- **North vs. South Group Sensitivity:** Lines 305-313 discuss how the mean ITCZ position changes its response to warming. I think these are the most interesting results, but there are two problems. First, most of the paper is in terms of north vs. south group, which refers to changes with warming not mean position (I have a comment on that below). Second, only one model is used for the sensitivity experiments, it is hard to come to these conclusions when only a model from the south group is tested. It would be very helpful to also run experiments with a model from the north group. (This is not as big a problem as the previous comment, but would still improve the paper)

- **Lack of Physical Understanding:** There are several aspects of the paper that are interesting but not explained physically
  - I believe the main result of the paper is that there is little intermodel correlation between AMOC strength and ITCZ position in the control climate (Fig. 2d), but there is a relationship between change in AMOC strength and change in ITCZ position (Fig. 3d). Why is this?
  - A lot of the paper is about a contrast between the ocean mechanism for ITCZ shifts (i.e., AMOC changes) and the atmospheric mechanisms (Hadley cell changes, AHT changes, and surface temperature changes). This is a very interesting concept, but the cause of the atmospheric mechanisms is not adequately discussed. Even just referring previous work would be an improvement, the reader is currently given no information about what controls the Hadley cell difference or surface temperature differences
- **Unclear Presentation Choices:** There are a few aspects of the presentation for which I don't understand the choices. These should be changed or explained
  - Why are all the models shown in Fig. 2 but only group averages in Fig. 3? The relevant data is somewhat shown in extended Fig. 1 and 2, but why wouldn't the two figures in the main text parallel each other?
  - Fig. 1 as a scatterplot would be nice, is there a correlation between ITCZ position and the change in ITCZ position?
  - An outline of the paper at the end of the introduction would be good to include. On first reading, the progression of results was not clear to me so knowing what is coming would help the reader
- **Methodological Questions:**
  - Transient vs. Long-term simulated responses/different warming scenarios: The paper uses a mix of different warming scenarios. The introduction mentions 1% increase, then Fig. 1-3 are about years 131-150 after a quadrupling of CO_2, and Fig. 4 is about the 1% experiments. It is fine to use a combination, but it should be made more explicit what is the purpose of each experiment and if the authors are considering each result to represent a steady-state warmed climate or a transient state
  - Forced response vs. internal variability in ERA5: The paper talks at length about the observed southward shift in the ITCZ (starting in the introduction and

throughout the paper). However, the top of Fig. 5 tells a more complicated and interesting story: that the ITCZ shifted south from 1940-1980 but then North from 1990-2020. How much of this is a forced response vs. internal variability? Reality is only one realization, so how can we tell? The proposed associations with other quantities in Fig. 5 seem to be based on correlations between three data points, which is not enough to convincingly show that one quantity is correlated with another, let alone establish a causal link as the authors frequently imply. Similarly, the sentence "If this assessment of AMOC variation is true, the observed southward ITCZ movement can be considered as another AMOC change proxy" seems very strange to me, as the figure just above shows that AMOC and ITCZ position are not always correlated (e.g., between the second red lines and third red lines they change in opposite directions).

Other Comments

- "North" and "South" group labels sound like they are referring to mean position. When reading the paper it is clear that they refer to changes in ITCZ position, but it is worth thinking about labels that would be clearer. Maybe "Northward" and "Southward" would be better
- The AMOC changes significantly in the "fixed AMOC" experiments. I understand the point is to keep the ITCZ in the same place, which it does successfully, but it might make sense to rename them because Fig. 4e clearly shows the AMOC changing
- The word variability is used on line 91 (and possibly other places), how is this defined?

Minor Comments

- The captions are indistinguishable from the main text. This will obviously be changed if the article is published, but it would be easier to review if the captions were slightly smaller or otherwise set apart
- Line 251: "mean longitudinal distribution of precipitation" -- is this meant to say the zonal mean? Or precipitation as a function of latitude?

Second Review of “Migration of Intertropical Convergence Zone Driven by Ocean Circulation Changes”

The new version of this paper is much improved from the previous and it is well on its way to being publishable. However, I still think that there are overstated conclusions, though these are now scattered throughout the text rather than in the title or abstract, and that this version has many typos that need to be fixed. What I believe is the manuscript's main contributions, that 1. ITCZ position and ITCZ shifts under warming are strongly anti-correlated, and 2. That the former is generally correlated with atmospheric mechanisms while the latter is correlated with AMOC changes, are scientifically interesting and important. The text should be written such that the focus is on these conclusions.

Major Comments

- **Overstated conclusions** -- Rather than focusing on the interesting and supported conclusions mentioned above, the manuscript often makes somewhat unrelated strong statements which are not supported by the evidence:
  - Line 365 states that Figure 4c “suggest[s] the existence of three possible quasi-stable ITCZ positions.” I believe what the authors are saying is that the states reached by year 400 of the simulations are quasi-stable. This conclusion seems reasonable to me, and it could be said that “these three ITCZ positions are quasi-stable.” However, by stating there are three possible positions, the authors seem to be implying that any other ITCZ positions, e.g. ones between -2 and -6, would not be stable. I do not believe there is evidence of this. If the AMOC strength increased slightly, would the ITCZ move slightly north? Are the authors trying to say that these three ITCZ positions are special in some way? Why would this be?
  - Internal variability vs. forced response in the historical period
    - Lines 404-406 states that because 25% is less than the CO_2 increase in the forced experiments, ITCZ variations “are primarily modulated by internal variability.” Just because CO_2 changes are smaller than that of an artificial experiment does not mean that observed variations are modulated by internal variability, as there is still some forcing. We do not know whether the historical ITCZ movement is variability or forced without more evidence
    - The following sentences, despite the above statement, act as if lines 404-406 are exactly wrong and treat the historical period as a forced response. This is also not necessarily true, as we do not know to what extent ITCZ movement is variability or forced

        - The historical period is likely strongly forced by aerosols as well as CO_2, this is not addressed and probably invalidates several of the paper's statements
        - Lines 408-413 states that a lack of perfect correlation between TS contrast and ITCZ position imply a role for the ocean. I do not believe this is true given the possible role of internal variability and aerosols. It would have to be established that the correlation between TS contrast and ITCZ is perfect in the absence of ocean forcing to support this statement.
    - Lines 499-500 still seem to say that we should use ITCZ migration as a proxy for AMOC weakening. Proxies are useful when the two quantities are well correlated across a long time period or with respect to the change in question. This is simply not the case for AMOC strength and the ITCZ, either in observations (where the three time periods do not even have the same relative sign in the two quantities) or in simulations (where a completely collapsed AMOC in CESM2 can lead to three different ITCZ positions). This should be removed or better supported
- **Abundance of typos --** There are enough typos throughout the paper for it to be distracting. I'm sure I missed some (especially later on in the paper), but here are the ones I found:
    - Line 30 -- "It is closely linked to Hadley Cell (HC)" -- should be "the Hadley Cells"
    - Line 56 -- "Equilibrate conditions" instead of "Equilibrium conditions"
    - Line 62 -- "Continuously warming world" not "Continuous warming world"
    - Line 96 -- "are multiply ways" should be "multiple ways"
    - Line 120 -- "19934" as a year
    - Line 150 -- "Such as" should be "As such"
    - Line 168-171 -- refers to "the correlations" between changes in variables "becoming stronger". These are correlations between changes in variables, so I assume they are not becoming stronger, they are just strong.

Other Comments

- Line 18 is hard to read, how about just saying "Earth system model simulations" without mentioning the "warmer background base state at this point"?
- The abstract is focused on models with a more northern ITCZ mean position -- it needs to be explained at least briefly in the abstract that this is because those are closer to reality
- Line 55-56 currently says "Based on this theory, changes in oceanic meridional heat transport across the equator are a simple response to the changes in AHT_eq as they covary under equilibrate conditions and are mechanically coupled over low latitudes"
    - I would disagree with this statement. The energy flux theory for ITCZ position leaves open the possibility of OHT as a forcing which can move the ITCZ, and many papers have explored the impact of such OHT
- The phrase "Warmer background base state" can be confusing because it is combining "base state," which sounds like the control, and "warmer." I think it would be clearer to just say "warmer climate" when possible

- Lines 96-113 is essentially a caveat and would be better if it were much shorter, in the methods section, or in an appendix. Having it at the beginning of a section which sounds like a results section distracts from the results
- Line 129 should clarify these are standard deviations with respect to time, rather than across models
- In scatter plots which show all models, the mean dots could be more prominent. I would recommend either fading out the individual models somewhat or putting an outline (black circle) on the ensemble mean points. Reanalysis products should probably be represented by points, not numbers
- Does it make physical sense to choose the “most consistent and realistic” models (132-134) based on changes with respect to warming rather than mean position and standard deviation?
- Line 147 -- “Control” hasn’t been defined in the text yet, only in the caption
- Is the initial northward shift of the ITCZ in the southward group statistically significant?
- Line 330-331 -- It is not necessary to mention temporary data availability issues
- In extended Data Fig. 2b, the caption should say the correlation coefficient with model 15 included. It is not an outlier for the changes, only for the control
